# Study on the Design, Synthesis, Bioactivity and Translocation of the Conjugates of Phenazine-1-carboxylic Acid and *N*-Phenyl Alanine Ester

**DOI:** 10.3390/molecules29081780

**Published:** 2024-04-14

**Authors:** Yiran Wu, Guoqing Mao, Gaoshan Xing, Yao Tian, Yong Hu, Changzhou Liao, Li Li, Xiang Zhu, Junkai Li

**Affiliations:** 1 Engineering Research Center of Ecology and Agricultural Use of Wetland, Ministry of Education, Hubei Key Laboratory of Waterlogging Disaster and Agricultural Use of Wetland, College of Agriculture, Yangtze University, Jingzhou 434025, China; wu18230085332@163.com (Y.W.); maoguoqing1688@163.com (G.M.); xinggaoshan6@163.com (G.X.); yaotien@163.com (Y.T.); yong251li@163.com (Y.H.); liaochangzhou58@163.com (C.L.); bohecha901@sina.com (L.L.); 2 Institute of Pesticides, Yangtze University, Jingmi Road 88, Jingzhou 434025, China; 3 National Key Laboratory of Green Pesticide, Key Laboratory of Green Pesticide and Agricultural Bioengineering, Ministry of Education, Guizhou University, Guiyang 550025, China

**Keywords:** phenazine-1-carboxylic acid, Metalaxyl, phloem mobility, fungicide, castor bean system, tobacco

## Abstract

The natural pesticide phenazine-1-carboxylic acid (PCA) is known to lack phloem mobility, whereas Metalaxyl is a representative phloem systemic fungicide. In order to endow PCA with phloem mobility and also enhance its antifungal activity, thirty-two phenazine-1-carboxylic acid-*N*-phenylalanine esters conjugates were designed and synthesized by conjugating PCA with the active structure *N*-acylalanine methyl ester of Metalaxyl. All target compounds were characterized by ^1^H NMR, ^13^C NMR and HRMS. The antifungal evaluation results revealed that several target compounds exhibited moderate to potent antifungal activities against *Sclerotinia sclerotiorum*, *Bipolaris sorokiniana*, *Phytophthora parasitica*, *Phytophthora citrophthora*. In particular, compound **F7** displayed excellent antifungal activity against *S. sclerotiorum* with an EC_50_ value of 6.57 µg/mL, which was superior to that of Metalaxyl. Phloem mobility study in castor bean system indicated good phloem mobility for the target compounds **F1**–**F16**. Particularly, compound **F2** exhibited excellent phloem mobility; the content of compound **F2** in the phloem sap of castor bean was 19.12 μmol/L, which was six times higher than Metalaxyl (3.56 μmol/L). The phloem mobility tests under different pH culture solutions verified the phloem translocation of compounds related to the “ion trap” effect. The distribution of the compound **F2** in tobacco plants further suggested its ambimobility in the phloem, exhibiting directional accumulation towards the apical growth point and the root. These results provide valuable insights for developing phloem mobility fungicides mediated by exogenous compounds.

## 1. Introduction

Crop disease is one of the natural disasters which seriously endangers agricultural production and global food security. Currently, using chemical fungicides to control crop diseases is still considered the most economical and effective method [1,2]. Among them, some crop root and vascular diseases are difficult to control through spraying because most systemic fungicides only acropetally translocate in the xylem. Therefore, many fungicides generally need to be applied by seed mixing and root irrigation [3,4,5]. However, seed coating and root irrigation treatments are accompanied by high cost, intensive labor and significant environmental pollution due to the complex nature of soil factors [6,7,8,9]. Only a small number of systemic fungicides exhibit phloem mobility in a plant, which can move basipetally to the plant vascular bundles and root parts via foliage spraying [10,11], such as Metalaxyl. But Metalaxyl has such serious resistance problems that it cannot meet the actual production requirements [12,13,14]. Therefore, developing phloem systemic fungicides to control root and vascular diseases of crops is of great significance.

**Figure 1:**
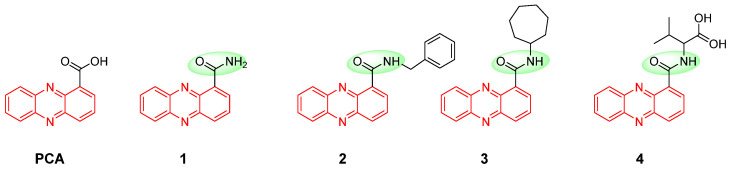
Phenazine-1-carboxylic acid and its derivatives.

Natural source pesticides with natural products (NPs) as active ingredients have many advantages, such as diverse modes of action, low toxicity and easy degradation [15,16]. Therefore, NPs are widely used as lead compounds in the development of new green pesticides [17,18]. Phenazine-1-carboxylic acid (PCA) is a significant natural product widely existing in the microbial metabolites of *Pseudomonads* (M18) and *Streptomycetes* which was registered as a fungicide against rice sheath blight in China. By reviewing the literature, we systematically compared previous studies on the structural modifications of the carboxyl site of PCA and found that its amide derivatives exhibited potent antifungal activities and phloem mobilities. For example, derivative **1** (Figure 1) possessed more significant antifungal activity against *Gaeumannomyces graminis* compared with PCA [19]. Notably, derivatives **2** and **3** (Figure 1) exhibited excellent antifungal activities against *Rhizoctonia solani*, with EC_50_ values of 0.008 and 0.003 μmol/L, respectively, which were superior to that of PCA (0.068 μmol/L) [20]. The PCA amino acid derivative **4** (Figure 1) not only had phloem mobility but also had more significant antifungal activity than PCA against tobacco *Rhizoctonia solani* in vivo [21]. At the same time, we also confirmed that its systemic mobility in plant phloem was an active transport process involving the *RcAAP*1 amino acid transporter carrier [22]. The above results demonstrated that PCA amide is a promising antifungal skeleton for discovering new fungicides.

Metalaxyl, a benzenamide fungicide, is widely used in the control of oomycetes. Its main active structure is *N*-acylalanine ester [23,24]. It is a representative phloem mobility fungicide. Because of its excellent phloem mobility property and effective fungicidal activity, it has been widely spread and used since the 1970s. However, the long-term overuse of Metalaxyl led to the rapid development of plant fungal resistance [25]. Currently, it is mainly used in combination with other fungicides.

**Figure 2:**
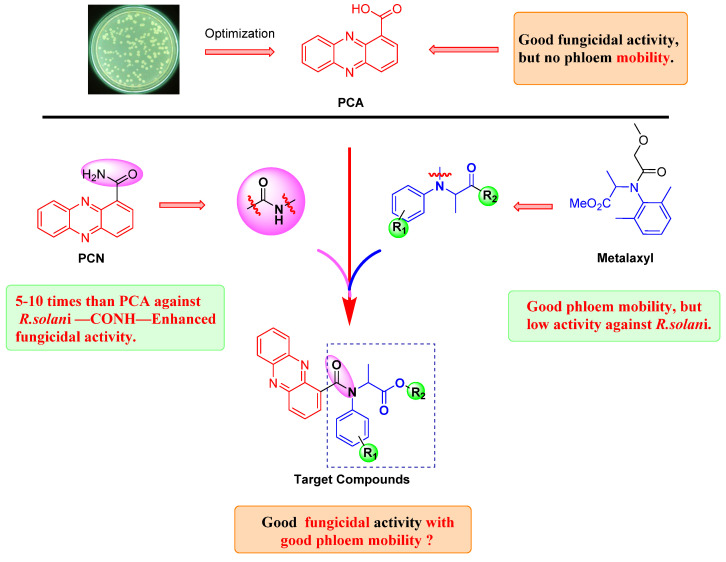
Design strategy of the target compounds.

On the premise of the above observation, in order to obtain higher antifungal activity and excellent phloem mobility of PCA derivatives, the target compounds were designed and synthesized by conjugating PCA with an active structure fragment of Metalaxyl based on the principle of active substructure splicing. The design strategy of the target compounds is shown in Figure 2. All the target compounds were screened for their antifungal activities against eight phytopathogenic fungi and evaluated for phloem mobility studies using the “castor seedling system” and “tobacco system”. Finally, the phloem mobility and distribution of compound **F2** in tobacco seedlings were further discussed.

## 2. Result and Discussion

### 2.1. Chemistry

**Scheme 1:**
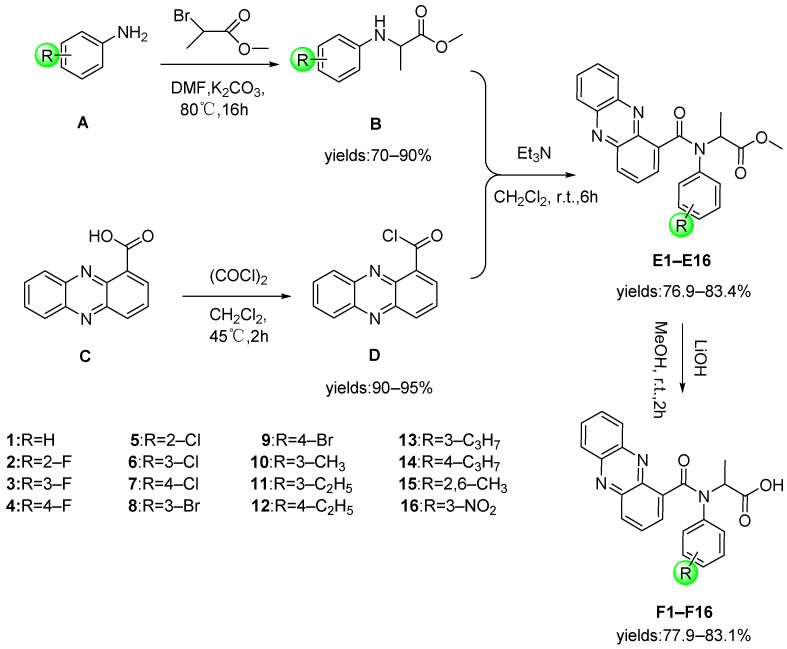
Synthetic routes of the target compounds.

The synthesis routes of target compounds **E1**–**E16** and **F1**–**F16** are outlined in Scheme 1. In brief, intermediate **B** was prepared by using methyl 2-bromopropionate ((*R*, *S*)-isomers) and the corresponding substituted aniline as the starting material [26]. Subsequently, intermediate **D** was obtained by the reaction of PCA and oxalyl chloride in CH_2_Cl_2_ [27]. Then, corresponding intermediate **B** was reacted with intermediate **D** in CH_2_Cl_2_ solution to yield the target compound **E** via an *N*-acylation of amines reaction [28]. Finally, the target compound **E** was hydrolyzed to obtain target compound **F** [29]. The structures of all the target compounds were characterized by ^1^H NMR, ^13^C NMR and HRMS. All corresponding signals of protons and carbons were recorded in the ^1^H and ^13^C NMR spectra. The HNMR spectra revealed the presence of isomers in the compound, and the amide bond exhibited a certain rigidity as a planar structure, resulting in NMR spectral characteristics similar to cis-trans isomers for the CH (CH_3_) group connected to the N atom. The CH-hydrogen signal and the CH_3_-hydrogen signal in the NCH (CH_3_) CO structural fragment both displayed two distinct peaks, respectively, with their total area consistent with the number of hydrogens present. Through the analysis of coupling constants, it was determined that these two peaks were not caused by spin–spin coupling splitting but rather resulted from different isomers. Additionally, since reactant B itself was a racemate, the target compound also consisted of a mixture of various isomers. In this study, no separation of isomers was performed; instead, they were directly utilized for bioactivity assays and translocation tests.

The spectral data of the target compounds were provided in the Supplementary Materials.

### 2.2. Antifungal Activity 

The preliminary antifungal activities of target compounds **E1**–**E16** and **F1**–**F16** against eight phytopathogenic fungi. (*S. sclerotiorum*, *B. sorokiniana*, *P. parasitica*, *P. citrophthora*, *A. solani*, *P. aphanidermatum*, *R. solani*, *P. infestans.*) were determined at a concentration of 50 µg/mL. The results in Table 1 indicated that most of the tested compounds exhibited remarkable antifungal activities against the eight phytopathogenic fungi compared with Metalaxyl. Meanwhile, most of the **F** series compounds demonstrated more excellent antifungal activities compared with **E** series compounds. Among them, compounds **F6**–**F14** possessed excellent antifungal activities against *S. sclerotiorum* with inhibition rates of more than 80%, which were higher than that of the commercial fungicide Metalaxyl (15.58%). The inhibition rates of compounds **F2** and **F11** against *P. citrophthora* and *A. solani* were more than 65%. And the inhibition rates of compounds **F2**, **F9** and **F16** against *B. sorokiniana* were 68.98%, 62.71% and 60.40%, respectively. Furthermore, compounds **F11** and **F14** exhibited excellent antifungal activities against *P. citrophthora* with inhibition rates of more than 50%. Unfortunately, **E** series compounds displayed poor antifungal activities against eight phytopathogenic fungi. Among them, only compound **E13** possessed moderate antifungal activities against *S. sclerotiorum*, *B. sorokiniana*, *P. parasitica* and *P. aphanidermatum*.

Compounds with activities of more than 80% were further tested for EC_50_ values to evaluate their excellent antifungal activities more accurately. The results are displayed in Table 2. Satisfactorily, the EC_50_ values of all tested compounds ranged from 6.75 µg/mL to 20.43 µg/mL. Particularly, compound **F7** possessed the most potent antifungal activity with the EC_50_ value of 6.57 µg/mL (16.22 μmol/L) against *S. sclerotiorum*, which was equivalent to that of the commercial fungicide PCA (17.28 μmol/L) at the Molarity.

### 2.3. Preliminary Analysis of Structure–Activity Relationship (SAR) 

The preliminary SAR results were deduced from the inhibitory activity data of the antifungal activities shown in Table 1 and Table 2. The results indicated that the type and position of substituents on the benzene ring had an impact on antifungal activities. Briefly, introducing an electron-donating group at the para-position could endow the final structure with better antifungal activity. For example, the antifungal activity of compound **F11** (R = 3-C_2_H_5_) was superior to that of compound **F6** (R = 3-Cl). In addition, introducing the same substituents at different positions of the benzene ring, the para-position was of great benefit for improving antifungal activity. For instance, the EC_50_ values of compounds **F7** (R = 4-Cl), **F5** (R = 2-Cl) and **F6** (R = 3-Cl) against *S. sclerotiorum* were 6.57 µg/mL, 14.57 µg/mL, 18.11 µg/mL, respectively. Moreover, by introducing the para-alkyl group of the benzene ring, the antifungal activity of the compound against *S. sclerotiorum* increased with the number of carbon atoms.

### 2.4. Phloem Mobility

**Figure 3:**
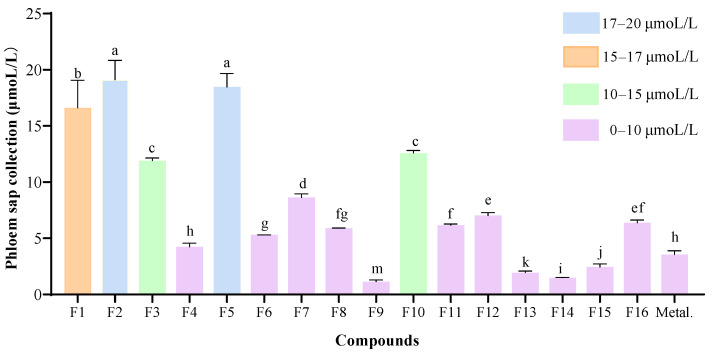
Content of target compounds **F1**–**F16** phloem sap of castor bean. Notes: those different letters are significantly different at the 5% level (LSD method).

To evaluate the phloem mobility of the target compounds, we used the castor bean system as a biological model, which was widely employed recently to study phloem uptake and translocation of xenobiotics. The linear equations of test compounds are shown in Table 3. The content of target compounds **F1**–**F16** phloem sap of castor bean was shown in Figure 3. The results indicated that PCA and **E**-series compounds did not exhibit phloem mobility. All **E**-series compounds were hydrolyzed to obtain **F**-series compounds. Interestingly, **F**-series compounds displayed excellent phloem mobility. Among them the phloem mobility concentrations of compounds **F10** and **F11** were five to six times higher than Metalaxyl. The results revealed that the pH difference between the inside and outside of the phloem cell membrane dictated the phloem mobility of xenobiotics. Specifically, the external pH was weak acidic, enabling weakly acidic F-series compounds to enter the phloem through diffusion. However, the alkaline nature of the phloem environment prevented the diffusion of ionic forms of F-series compounds out of the plasma membrane, resulting in a degree of phloem formation and accumulation with limited conductivity. On the other hand, the E series compounds are esters, which are more stable in acidic environments and cannot enter the plasma membrane by diffusion, so they have no phloem mobility. These findings underscore the significance of carboxylic acid group ionization in determining the phloem mobility of compound pairs.

**Figure 4:**
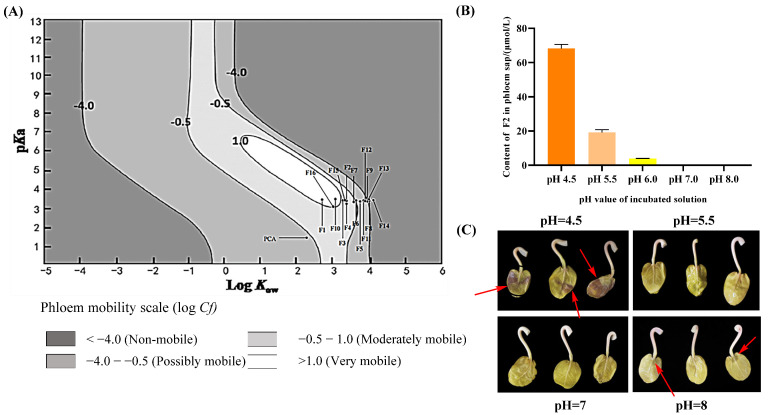
(**A**) Prediction of phloem mobility of compounds **F1**–**F16** and **PCA** using the Kleier map (log C*f* as a function of p*K*a and Log *K*ow). (**B**) Phloem transport of **F2** in the caster bean model at different external pH values. (**C**) Leaf states in the caster bean model at different external pH values (Arrows indicate the site of leaf damage).

To further investigate the possible mobility mechanism of the target compound **F**, compound **F2** with the best phloem mobility was further assayed under a buffer solution with different pH conditions (Figure 4B). With the increasing pH of the buffer solution, the detected concentration of compound **F2** gradually decreased. When pH = 4.5, the phloem mobility concentration of **F2** was 68.16 μmol/L; When pH = 5.5, the phloem mobility concentration of **F2** was 19.12 μmol/L. When pH = 7 and 8, the phloem mobility concentration of **F2** approached zero. The results displayed that the change in pH had a significant effect on the phloem mobility of compounds. However, it can be clearly seen from Figure 4C that excessive acid or alkalinity could cause damage to plant tissues and affect leaf absorption. The proper weak acid condition was more conducive to the mobility of compounds in the phloem. With pH increasing from 4.5 to 8, the detected concentration of compound **F2** gradually reduced to zero. Compound **F2** was ionized during the increase in pH; its hydrophilicity was increased, leading to the weakened phloem mobility. The results showed the introduction of the carboxylic acid groups could change both inside and outside phloem mobility under different pH conditions. And the results indicated that the “ion trap” effect played an important role in the phloem transport of compounds.

### 2.5. Predicting Phloem Mobility and Possible Mobility Mechanism of F Series Compounds 

The Kleier model is widely employed to predict whether the compounds possess phloem mobility based on their physical–chemical properties (log*Kow* and p*Ka*, respectively) [30,31,32]. Therefore, the log*Kow* and p*Ka* of tested compounds **F1**–**F16** (Table 4) were introduced into the Kleier model to predict their phloem mobility. The predicted results in Figure 4A indicated that other compounds and PCA all possessed phloem mobility except for compounds **F8**, **F13** and **F14**. Among them, compounds **F1**, **F10** and **F16** theoretically belonged to the range of “Very mobile”; compounds **F2**, **F3**, **F4**, **F7** and **F15** belonged to the range of “Moderately mobile”; compounds **F5**, **F6**, **F9**, **F11**, **F12** belonged to the range of “Possibly mobile”. However, the phloem mobility test results on the castor beans system showed that other test compounds all exhibited phloem mobility except PCA. That was inconsistent with the Kleier model, which was mainly used to explain the free diffusion characteristics of substances. Thus, it could be speculated that some of the target compounds did not completely conform to the passive transport-free diffusion mechanism but may also involve active transport mechanisms. However, whether the tested compounds involved active transport on castor phloem requires further investigation. 

### 2.6. The Phloem Mobility of Compound ***F2*** Up-Taken through Tobacco Seedlings

**Figure 5:**
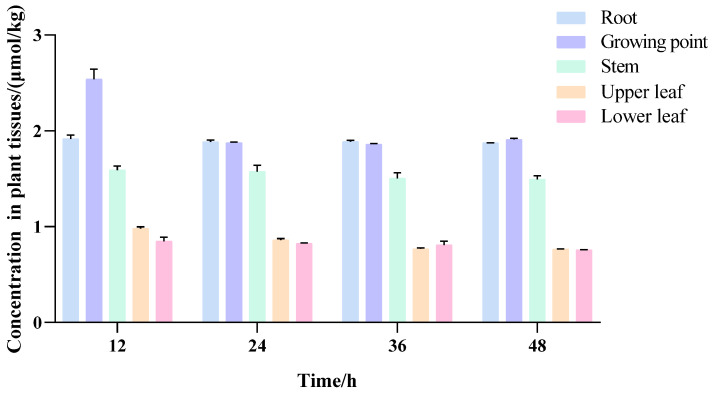
Translocation of compound **F2** in different parts of tobacco seedlings.

The compound **F2** of optimal mobility was used to verify the mobility distribution of the compounds in tobacco plants. In order to verify the feasibility of this method, the root, stem and leaf of blank samples were, respectively, added to three concentrations of compound solution. Then, the samples were dealt with using the preliminary extraction and purification displayed in Section 3.6 methods. The detection method was the same as in Section 3.5.2. The results provided in Table 5 showed that the method could completely satisfy the recovery requirements of extraction and separation of samples. Subsequently, the growing point, upper leaves, lower leaves, stems and roots were cut at different times after treatment and extracted and detected according to the method in Section 3.6. The results at different times (Figure 5) showed that compound **F2** accumulated in roots, growth points, stems and lower and upper leaves. Interestingly, compound **F2** could rapidly distribute to the whole tobacco plant within a short time and reach the maximum accumulation. Among them, compound **F2** had the highest accumulation in root and growth points with concentrations of 1.92 μmol/kg and 2.54 μmol/kg at 12 h, respectively. Secondly, the concentration of accumulations in the stem and the lower and upper leaves were 1.59 μmol/kg, 0.85 μmol/kg, 0.98 μmol/kg, respectively. The results suggested that **F2** could ambimobile through the phloem and xylem in tobacco seedlings and accumulate selectively in the apical meristem and roots. The possible reason for this is that plant roots and apical meristem are the most vigorous parts of plant growth, and the compound **F2** can accumulate in the vigorous parts of plant growth under the action of transpiration and nutrient flow after entering the vascular bundle. This result will provide a new direction for the development of novel pesticides to address the problems of the difficulty of controlling root and vascular diseases through foliar spray. 

## 3. Materials and Methods

### 3.1. Chemicals and Instruments

Phenazine-1-carboxylic acid (PCA) and Metalaxyl were provided by the College of Agriculture, Yangtze University. All chemicals and solvents were commercially available without further purification. All target compounds were further purified by column chromatography using silica gel (200–300 mesh, Liang Chen Gui Yuan Co., Ltd., Luan, China). Thin layer chromatography (TLC) analysis was performed on silica gel GF254 (Qingdao Hai Yang Chemical Co., Ltd., Qingdao, China). ^1^H NMR and ^13^C NMR spectra were recorded on an AVANCE DPX400 nuclear magnetic resonance spectrometer (Bruker Spectroscopy, France, Germany) using trimethylchlorosilane (TMS) and DMSO-*d*_6_ or CDCl_3_ as the internal standard and the solvent, respectively. High-resolution mass spectra (HRMS) data were acquired on Thermo Scientific Q Exactive (Thermo Fisher Scientific, Waltham, MA, USA). The melting points of all target compounds were confirmed by using a WRR melting point apparatus (Shanghai Jingke Industrial Co., Ltd., Shanghai, China) and are uncorrected.

### 3.2. Test Fungus and Plant Materials

Eight phytopathogenic fungi were used in the experiment, including *Sclerotinia sclerotiorum* (*S. sclerotiorum*), *Bipolaris sorokiniana* (*B. sorokiniana*), *Phytophthora parasitica* (*P. parasitica*), *Phytophthora citrophthora* (*P. citrophthora*), *Alternaria solani* (*A. solani*), *Pythium aphanidermatum* (*P. aphanidermatum*), *Rhizoctonia solani* (*R. solani*), *Phytophthora infestans* (*P. infestans*) were provided by the Plant Pathology Laboratory, Yangtze University. The castor seeds were provided by Shandong Zibo Academy of Agricultural Sciences. Castor bean seedlings were cultured for 6 days at 28 °C in an artificial climate chamber. Seeds of tobacco cv. Yunyan 87 were obtained from the Plant Pathology Laboratory, Yangtze University

### 3.3. Synthesis

#### 3.3.1. General Procedure for the Preparation of **B**

In a 250 mL round-bottom flask, the aniline **A** (20 mmol) and potassium carbonate (20 mmol) were dissolved in dry *N*, *N*-dimethylformamide (DMF, 15 mL). Methyl-2-bromopropionate (25 mmol) was added dropwise and stirred at 80 ℃ for 16 h. After the completion of reaction (monitored by TLC), the solvent was quenched by adding water (100 mL) and extracted with ethyl acetate (3 × 100 mL). Subsequently, the organic layer was dried with anhydrous magnesium sulfate. The residue was purified by silica gel column chromatography (petroleum ether/EtOAc = 10:1, *v*/*v*) to obtain intermediates **B** [26].

#### 3.3.2. General Procedure for the Preparation of Intermediate **D**

In a 250 mL round-bottomed flask, oxalyl chloride (4 mL) and DMF (0.1 mmol) were added dropwise to a solution of compound **C** (10 mmol) in dry dichloromethane (CH_2_Cl_2_, 15 mL). The reaction mixture was stirred and refluxed for 2 h. After the reaction was completed (monitored by TLC), the reaction solution was concentrated to obtain the crude intermediate **D** [27].

#### 3.3.3. General Procedure for the Preparation of Target Compounds **E**

In a 250 mL round-bottomed flask, intermediate **B** (10 mmol) was added to a stirred solution of crude intermediate **D** (10 mmol) and triethylamine (20 mmol) in CH_2_Cl_2_ (10 mL). The mixture was maintained at room temperature until intermediate **B** was completely reacted (monitored by TLC). Then, the solvent was removed by vacuum. The residue was purified by column chromatography on silica gel and eluted with a mixture of petroleum ether and EtOAc (PE-EtOAc), *v*/*v* = 5:1) to afford the target compound **E** [28]. The spectral data of target compounds **E1−E16** were provided in the Supplementary Materials.

#### 3.3.4. General Procedure for the Preparation of Target Compound **F**

In a 100 mL round-bottomed flask, lithium hydroxide (7.5 mmol) was added to a solution of compound **E** (2.5 mmol) in water (10 mL) and methanol (10 mL). The reaction was maintained for 2 h until the reaction was completed (monitored by TLC). Then, the pH of the reaction mixture was adjusted to 2 with hydrochloric acid until solid product was precipitated. Eventually, the mixture was filtered and washed with water to give the target compound **F** [29]. The spectral data of target compounds **F1**–**F16** were available in the Supplementary Materials.

### 3.4. Antifungal Activity Assay

The antifungal activities of all the target compounds against *S. sclerotiorum*, *B. sorokiniana*, *P. parasitica*, *P. phthora*, *A. solani*, *P. aphanidermatum*, *R. solani*, *P. infestans* were screened using the mycelium growth rate test [33]. Firstly, each test compound was dissolved in 0.5% dimethylsulfoxide (DMSO) containing 0.1% Tween 80 and diluted to the corresponding concentration with sterile water. Secondly, each prepared solution was added to sterile molten potato dextrose agar (PDA) medium to acquire a final tested concentration (50 μg/mL). Then, the PDA was decanted into 70 mm sterilized petri plates with 15 mL per plate. Thirdly, the mycelial disks (diameter = 7 mm) cut from subcultured PDA dishes were inoculated in the PDA medium. The inoculated PDA dishes were incubated at 26 ± 2 ℃. PDA mediums containing 0.5% DMSO containing 0.1% Tween 80 were used as a blank control, and the commercial fungicides PCA and Metalaxyl were employed as positive controls. Each sample was measured in triplicate. After the mycelium diameter of the blank control reached 55 mm–60 mm, the mycelium diameters (mm) of each sample were exactly measured by the cross-bracketing method. The growth inhibition rates were calculated according to the following Formula (1).

Inhibition rate (%) = [(C − T)/(C − 7 mm)] × 100(1)

where C and T represent the diameters of fungal growth on untreated PDA and treated PDA, respectively, and 7 mm is the diameter of mycelial disks. The growth inhibition rates and the standard errors were calculated by Microsoft Excel 2016 (Version 16.0.17029.20028) software.

According to the above-mentioned procedures and the screening results, target compounds with inhibition rates higher than 80% were further assayed for their median effective concentration (EC_50_) values. PDA mediums containing 80, 50, 40, 25, 12.5, 6.25 and 3.125 µg/mL of the tested compound were prepared, and their antifungal activities were evaluated by accurately measuring the inhibition rate against the corresponding fungi. The log dose-response curves were used for the determination of the EC_50_ values by using Data Processing System (DPS) (Version 20.05) software.

### 3.5. Phloem Sap Collection and Analysis

#### 3.5.1. Phloem Sap Collection

The phloem mobility of target compounds was evaluated by castor seedling system according to our previously reported procedures [34]. Primarily, the castor cotyledons were washed. Then, they were incubated in buffered solution (pH = 5.5) containing Na_2_HPO_4_ (200 mmol/L, 5 mL), NaH_2_PO_4_ (200 mmol/L, 92 mL) and 200 µmol/L of every target compound for 6 h. At the same time, the roots were cultivated in deionized water containing 0.5 mmol/L calcium chloride for 2 h. The hypocotyl was severed at the hook of seedlings after 2 h, and the roots were abandoned. Finally, phloem sap between 2 and 4 h was collected for detection. 

To further discuss the possible phloem mobility mechanism of the target compounds **F**, the castor cotyledons were immersed in solution containing 200 µmol/L of every target compound with different pH values (pH = 4.5, 5.5, 6.0, 7.0 and 8.0) to collect and determine the phloem sap according to the above method [35]. All the experiments were carried out in triplicate.

#### 3.5.2. Analytical Methods

The phloem sap was diluted with pure water (phloem sap/pure water = 1:4, *v*/*v*). The solution was filtered with a 0.22 µm aqueous phase filter before being analyzed by Thermo UltiMate3000 TSQ-Guantis (HPLC-MS) instrument. An agilent C18 reversed-phase column (5 µm, 150 × 4.6 mm inner diameter) was used for separations at 40 °C. The mobile phase consisted of methanol and water (70:30, *v*/*v*) at a flow rate of 0.3 mL/min, and the injection volume was 2 μL. An external calibration method was used to quantify the title compounds. A series of standard solutions of test compounds (5, 10, 20, 40 and 50 μmol/L) for linearities were prepared in methanol.

### 3.6. Mobile Distribution of Compound ***F2*** in Tobacco

#### 3.6.1. Uptake and Translocation of Compound **F2** by Tobacco Seedlings 

The translocation distribution of compound **F2** was evaluated by tobacco seedlings system, which were reported in our previous procedures [21]. Tobacco seedlings were employed to investigate the uptake and translocation at the 10–12 leaf stage. Firstly, the seedlings were divided into six parts: (i) apical growth point, (ii) stem, (iii) upper leaves, (iv) mature leaves, (v) lower leaves and (vi) roots. The phloem mobility of **F2** was executed by applying its solution to mature leaves. Secondly, compound **F2** was dissolved in 2 mL DMSO and diluted with aqueous 1% Tween 80 to a concentration of 400 μmol/L. Thirdly, the solution was evenly smeared on both sides of the mature tobacco leaves with a brush. Finally, fresh weight (2–5 g) of apical leaves, stem, mature leaves, lower leaves and roots were harvested from different plants at 12, 24, 36 and 48 h after treatment, and these samples were stored at −20 °C until further extraction and analysis. Each treatment was carried out in triplicate.

#### 3.6.2. Extraction and Analysis of Compounds **F2**

The collected samples (apical growth point, 3 g; stem, 3 g; lower leaves, 5 g; upper leaves, 5 g; and roots, 2 g) from various parts of tobacco seedlings were washed and triturated. Firstly, the triturated samples were ultrasonically extracted for 10 min with methanol (100 mL). After filtration, the residue was extracted three times with methanol (40 mL, 30 mL, 30 mL), and all methanol phases were merged and concentrated to dry [21]. Then, each sample was transferred to a 50 mL centrifuge tube, and the volume was constant to 10 mL. Sodium chloride (1 g) and magnesium sulfate (4 g) were added, vortexed for 1 min, 5000 rpm/min, then centrifuged for 5 min. The supernatant (4 mL) was taken into a 10 mL centrifuge tube, 200 mg C18 added, vortexed for 1 min, 5000 rpm/min and centrifuged for 5 min [36]. Finally, the supernatant was extracted and then tested.

#### 3.6.3. Spike Recovery

Different tobacco tissues with corresponding weights were weighed (apical growth point, 3 g; stem, 3 g; leaves, 5 g and roots, 2 g). The standard solution of 400, 100 and 40 μmol/L of **F2** was added to the tissue of each site, respectively. Each treatment was repeated three times. Then, the samples were dealt with the preliminary extraction and purification by Section 3.6.2 methods. The detection method was the same as in Section 3.5.2. The recovery was calculated according to the following Formula (2).

Recovery (%) = C2/C1 × 100%(2)

where C1 represents average spiked sample concentrations of compound (μmol/L) and C2 represents the real sample average concentrations of compound. The average recoveries were calculated by Microsoft Excel 2016 software and Data Processing System (DPS) (Version 20.05) software.

## 4. Conclusions

In conclusion, thirty-two PCA-*N*-phenylalanine (ester) conjugates were designed and synthesized by the substructure splicing method. All target compounds were structurally characterized by ^1^H NMR, ^13^C NMR and HRMS. The antifungal results indicated that **F**-series compounds exhibited favorable activities against *S. sclerotiorum*. Among them, compound **F7** possessed excellent antifungal activity against *S. sclerotiorum* with an EC_50_ value of 6.57 μg/mL (16.22 μmol/L), which was superior to that of Metalaxyl. The phloem mobilities of all target compounds were further assayed in the castor bean system. The **F**-series compounds exhibited excellent phloem mobility. Particularly, compound **F2** showed the best phloem mobility with an average concentration of 19.12 μmol/L in plant phloem, which was more significant than the systemic fungicide Metalaxyl (3.56 μmol/L). Combined with the Kleier model and phloem mobility of compound **F2** at different pH values, the results deduced that the phloem mobility of compound **F** was related to the “ion trap” effect. Subsequently, the distribution of compound **F2** in tobacco plants was further evaluated using the tobacco system. The results indicated that compound **F2** was rapidly accumulated to the root, stem, leaves and apical growth point of tobacco seedlings and reached maximum accumulation within a short time. The results confirmed that compound **F2** had excellent ambimobile of compound **F2** in the phloem and xylem.

The research and development of phloem mobility fungicides is of great significance for effectively controlling plant root diseases and vascular diseases through spraying. In this study, 16 new compounds with phloem mobility were synthesized by conjugating the active substructure of the fungicide Metalaxyl with the natural fungicide PCA. However, the translocation test results of these compounds showed significant deviations from the predictions of the Kleier model, indicating the possibility of active transport. Further in-depth research on the rules of phloem translocation of the foliar-applied xenobiotic, such as pesticides, will be an important task in the future.

## Figures and Tables

**Table 1:** Antifungal activities of the target compounds **E1**–**E16** and **F1**–**F16** against eight fungi at 50 µg/mL.

Compd. ^b^	Average Inhibition Rate ± SD (%) (*n* = 3)							
	*S. S* ^a^	*B. s*	*P. p*	*P. c*	*A. s*	*P. a*	*R. s*	*P. i*
**E1**	<10	<10	13.36 ± 2.13	<10	<10	16.39 ± 1.27	<10	<10
**E2**	21.38 ± 3.13	14.06 ± 0.08	<10	<10	22.98 ± 2.76	<10	<10	10.26 ± 2.16
**E3**	17.76 ± 2.88	10.63 ± 1.03	<10	<10	19.09 ± 1.90	<10	<10	<10
**E4**	<10	10.00 ± 0.49	19.80 ± 1.26	<10	12.30 ± 3.07	<10	<10	<10
**E5**	19.74 ± 4.54	<10	<10	<10	21.68 ± 4.28	<10	<10	12.91 ± 1.85
**E6**	<10	<10	<10	<10	<10	<10	<10	<10
**E7**	<10	<10	<10	<10	<10	11.11 ± 0.48	<10	<10
**E8**	22.70 ± 3.62	14.38 ± 1.37	<10	23.44 ± 1.69	15.53 ± 4.77	<10	10.20 ± 5.49	<10
**E9**	<10	<10	10.74 ± 3.11	<10	15.86 ± 3.49	<10	<10	<10
**E10**	29.35 ± 2.80	<10	22.15 ± 0.82	<10	19.49 ± 1.09	22.78 ± 1.92	<10	<10
**E11**	38.16 ± 1.63	45.00 ± 0.74	41.61 ± 0.80	29.69 ± 1.28	44.66 ± 1.43	50.90 ± 4.04	32.86 ± 2.28	24.5 ± 1.79
**E12**	16.45 ± 4.33	12.19 ± 0.07	22.15 ± 0.68	<10	16.18 ± 1.99	25.00 ± 1.40	10.20 ± 1.53	<10
**E13**	57.89 ± 1.17	49.38 ± 0.71	59.06 ± 1.19	42.81 ± 0.31	38.51 ± 2.15	57.83 ± 2.16	40.23 ± 1.53	26.16 ± 0.77
**E14**	55.59 ± 0.82	33.13 ± 0.48	22.15 ± 3.34	17.19 ± 4.57	23.95 ± 3.89	27.41 ± 4.74	23.23 ± 1.73	<10
**E15**	<10	<10	<10	12.32 ± 8.89	12.64 ± 1.65	13.33 ± 1.67	<10	<10
**E16**	<10	10.31 ± 0.89	<10	<10	19.09 ± 3.36	<10	<10	<10
**F1**	33.70 ± 3.56	11.22 ± 0.48	33.33 ± 2.22	<10	<10	<10	53.14 ± 2.70	22.15 ± 2.49
**F2**	44.93 ± 1.33	68.98 ± 0.90	26.74 ± 1.79	10.15 ± 0.20	66.08 ± 0.58	54.29 ± 0	29.04 ± 5.35	19.54 ± 1.41
**F3**	46.01 ± 0.39	<10	20.83 ± 1.36	22.46 ± 2.41	<10	17.73 ± 0.50	20.13 ± 1.88	14.66 ± 0.70
**F4**	78.62 ± 1.57	<10	47.22 ± 0.40	28.31 ± 1.63	<10	13.30 ± 1.26	20.79 ± 0.90	26.38 ± 1.88
**F5**	81.16 ± 0.53	<10	40.28 ± 2.44	16.92 ± 1.61	<10	13.85 ± 0.58	31.35 ± 1.21	41.04 ± 1.05
**F6**	81.52 ± 1.17	11.88 ± 0.87	27.78 ± 0.95	<10	<10	13.57 ± 1.50	32.67 ± 0.87	28.99 ± 0.79
**F7**	81.52 ± 1.03	<10	49.31 ± 2.05	12.31 ± 3.29	<10	20.22 ± 1.00	22.44 ± 1.84	27.69 ± 1.42
**F8**	78.99 ± 0.38	10.89 ± 1.87	25.00 ± 1.16	<10	<10	<10	30.36 ± 2.31	29.64 ± 2.19
**F9**	81.52 ± 0.89	62.71 ± 0.31	47.22 ± 1.18	13.23 ± 1.68	47.95 ± 0.29	26.32 ± 0.76	26.40 ± 2.27	34.85 ± 0.68
**F10**	71.74 ± 0.50	11.22 ± 1.40	43.75 ± 1.15	11.08 ± 2.25	<10	17.45 ± 0.76	42.90 ± 2.37	25.73 ± 1.36
**F11**	81.16 ± 0.39	46.20 ± 0.78	35.07 ± 0.26	66.77 ± 0.35	24.85 ± 0.29	22.16 ± 0.58	42.57 ± 1.37	30.62 ± 1.73
**F12**	70.29 ± 0.40	13.86 ± 0.85	43.40 ± 1.50	11.38 ± 1.75	<10	<10	24.75 ± 1.52	57.65 ± 0.80
**F13**	80.80 ± 0.78	16.17 ± 0.44	26.39 ± 1.51	20.31 ± 2.07	<10	14.13 ± 1.04	36.63 ± 0.63	39.41 ± 0.33
**F14**	83.70 ± 0.27	15.18 ± 1.36	37.15 ± 0.26	51.38 ± 2.80	15.20 ± 1.15	21.33 ± 0.76	8.58 ± 6.36	63.19 ± 0.20
**F15**	35.51 ± 1.16	<10	37.50 ± 1.24	10.46 ± 0.74	11.70 ± 0.29	14.13 ± 0.58	43.23 ± 5.87	19.54 ± 1.17
**F16**	19.57 ± 1.16	60.40 ± 1.38	<10	<10	29.24 ± 0.29	23.55 ± 0.50	19.47 ± 0.42	12.38 ± 1.09
**PCA ^c^**	98.55 ± 0.51	100.00 ± 0	85.76 ± 0.65	68.00 ± 1.06	75.73 ± 0.29	73.13 ± 0.29	93.07 ± 1.66	89.25 ± 0.98
**Metal. ^d^**	15.58 ± 1.80	<10	80.56 ± 0.71	<10	24.56 ± 0	16.62 ± 0.29	56.11 ± 1.23	<10

^a^ *S. s*: *S. sclerotiorum*, *B. s*: *B. sorokiniana*, *P. p*: *P. parasitica*, *P. c*: *P. citrophthora*, *A. s*: *A. solani*, *P. a*: *P. aphanidermatum*, *R. s*: *R. solani*, *P. i*: *P. infestans*. ^b^ Compd.: compound. ^c^ PCA: Phenazine-1-carboxylic acid. ^d^ Metal.: Metalaxyl.

**Table 2:** EC_50_ values of target compounds against *S. sclerotiorum*.

Compd.	EC_50_/(μg/mL)	EC_50_/(μmol/L)	Regression Equation	95% Confidence Interval/(μg/mL)	Correlation Coefficient (R^2^)
**F5**	14.57	35.98	y = 3.2851 + 1.4740x	13.1904–16.0933	0.9961
**F6**	18.11	44.72	y = 2.6425 + 1.8741x	15.7107–20.8773	0.9924
**F7**	6.57	16.22	y = 4.0718 + 1.1352x	5.5046–7.8456	0.9939
**F9**	9.92	22.09	y = 3.5751 + 1.4296x	9.1232–10.7955	0.9972
**F11**	20.43	51.20	y = 2.5061 + 1.9032x	16.6768–25.0367	0.9742
**F13**	16.97	41.09	y = 3.0535 + 1.5830x	15.5039–18.5692	0.9965
**F14**	8.02	19.42	y = 3.8083 + 1.3183x	6.8062–9.4396	0.9939
**PCA**	3.87	17.28	y = 4.1864 + 1.3840x	3.218–4.6567	0.9901

Commercial fungicide Phenazine-1-carboxylic acid (PCA).

**Table 3:** Linear equations and correlation coefficients of the HPLC-MS method for quantification of the test compounds.

Compd.	Regression Equation	Correlation Coefficient (R^2^)
**F1**	y = 11034099x − 10368260	0.9927
**F2**	y = 10613651x − 650875	0.9990
**F3**	y = 1202838x − 5885	0.9997
**F4**	y = 2065740x − 44534	0.9962
**F5**	y = 2234165x − 1020903	0.9992
**F6**	y = 6126714x − 6610569	0.9972
**F7**	y = 6695972x − 3185573	0.9952
**F8**	y = 5314495x − 2033452	0.9994
**F9**	y = 1173264x − 85687	0.9980
**F10**	y = 7134918x − 6908171	0.9983
**F11**	y = 12128866x + 5301627	0.9998
**F12**	y = 6795334x − 1939715	0.9991
**F13**	y = 11573595x − 985307	1.0000
**F14**	y = 2148578x − 669546	0.9992
**F15**	y = 14370508x + 103540	0.9998
**F16**	y = 3231821x − 2254916	0.9991
**Metal.**	y = 922409x + 211247	0.9995
**PCA**	y = 27002x − 2443	0.9971

Commercial fungicides Phenazine-1-carboxylic acid (PCA) and Metalaxyl (Metal.).

**Table 4:** Predicted physicochemical properties of compounds **F1**–**F16**, **PCA** and **Metal**.

Compd.	Molecular Weight (g/mol)	Log*Kow*	p*Ka*
**F1**	371.40	2.74	3.447
**F2**	389.39	3.38	3.322
**F3**	389.39	3.30	3.374
**F4**	389.39	3.38	3.239
**F5**	405.84	3.70	3.299
**F6**	405.84	3.67	3.365
**F7**	405.84	3.64	3.285
**F8**	450.29	3.99	3.363
**F9**	450.29	3.97	3.303
**F10**	385.42	3.13	3.481
**F11**	399.45	3.82	3.461
**F12**	399.45	3.87	3.415
**F13**	413.48	3.98	3.442
**F14**	413.48	4.05	3.408
**F15**	399.45	3.37	3.396
**F16**	415.39	3.09	3.156
**PCA**	224.22	2.34	1.59
**Metal.**	279.34	--	--

Commercial fungicides Phenazine-1-carboxylic acid (PCA) and Metalaxyl (Metal.). Notes: The “Log*K*ow” was calculated by the ALOGPS 2.1 program; the “p*K*a” was calculated by ChemDraw 21.0.0.

**Table 5:** The recoveries of the tested compound **F2** in plant samples of tobacco seedlings.

Detected Organs of Seeding	Added Content (μmol/L)	Detected Content (μmol/L)	Average Recoveries (%)	Coefficient of Variation (%)
Foliage	400	301.21	75.30 ± 1.54	2.05
	100	87.65	87.65 ± 3.62	3.85
	40	42.40	106.00 ± 2.26	2.13
Stem	400	335.75	83.94 ± 4.26	5.07
	100	104.16	104.16 ± 2.59	2.49
	40	43.93	109.83 ± 1.39	1.27
Root	400	309.31	77.33 ± 1.44	1.86
	100	70.67	70.67 ± 5.10	7.22
	40	29.22	73.04 ± 3.27	4.48

Note: Each treatment had three replicates (Mean ± SD). The method recovery rate should range from approximately 70 to 110% (the pesticide registration rule of residual test (China)).

## Data Availability

Data are contained within the article and Supplementary Materials.

## Supplementary Materials

The following supporting information can be downloaded at: https://www.mdpi.com/article/10.3390/molecules29081780/s1, The structural characterization data of target compounds **F1**–**F16**, **E1**–**E16**.
