# Supplementary material for: Study on the Design, Synthesis, Bioactivity and Translocation of the Conjugates of Phenazine-1-carboxylic Acid and N-Phenyl Alanine Ester
Source: Molecules. 2024 Apr 14;29(8):1780. doi: 10.3390/molecules29081780 (PMC11051964; doi:10.3390/molecules29081780)
Supplement: Supplementary file 1 [file molecules-29-01780-s001.zip › molecules-2917221-supplementary.pdf]

Supporting Information

Study on the Design, Synthesis, Bioactivity and Translocation of the Conjugates of Phenazine-1-carboxylic Acid and *N*-Phenyl Alanine Ester

Yiran Wu [1,2], Guoqing Mao [1,2], Gaoshan Xing [1,2], Yao Tian [1,2], Yong Hu [1,2], Changzhou Liao [1,2], Li Li [1,2], Xiang Zhu [1,2,3,*] and Junkai Li [1,2,*]

[1] Engineering Research Center of Ecology and Agricultural Use of Wetland, Ministry of Education, Hubei Key Laboratory of Waterlogging Disaster and Agricultural Use of Wetland, College of Agriculture, Yangtze University, Jingzhou 434025, China; wu18230085332@163.com (Y.W.); maoguoqing1688@163.com (G.M.); xinggaoshan6@163.com (G.X.); yaotien@163.com (Y.T.); yong251li@163.com (Y.H.); liaochangzhou58@163.com (C.L.); bohecha901@sina.com (L.L.)

[2] Institute of Pesticides, Yangtze University, Jingmi Road 88, Jingzhou 434025, China

[3] National Key Laboratory of Green Pesticide, Key Laboratory of Green Pesticide and Agricultural Bioengineering, Ministry of Education, Guizhou University, Guiyang 550025, China

* Correspondence: xiangzhu1992@yangtzeu.edu.cn (X.Z.); junkaili@sina.com (J.L.)

Table of Contents

1.The structure and data of target compounds **(F1-F16)** **2**

2.The structure and data of target compounds **(E1-E16)** **34**

Compound **F1**,

N-(phenazine-1-carbonyl)-*N*-phenylalanine

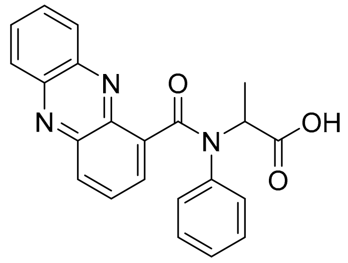

Yellow solid, yield 80.1%, m.p. 130.9-132.1°C; ^{1}H NMR (400 MHz, DMSO-d_6) δ 12.93 (s, 1H), 8.34 (dd, *J* = 14.2, 7.2 Hz, 1H), 8.22 (d, *J* = 8.4 Hz, 1H), 8.15 – 7.92 (m, 3H), 7.86 – 7.62 (m, 2H), 7.22 (d, *J* = 6.8 Hz, 2H), 7.05 – 6.70 (m, 3H), 5.15 (1H, two isomers), 1.47 (3H, two isomers). ^{13}C NMR (101 MHz, DMSO-d_6) *δ* 173.57, 168.03, 143.19, 142.80, 142.24, 139.90, 137.60, 132.00, 130.53 (2C), 130.26 (2C), 129.96 (2C), 129.73, 128.60, 128.00, 127.35, 124.10 (2C), 66.83, 15.88. HRMS (ESI): calcd for $C_{23}H_{17}N_3O_3$ {[M+H]$^+$}, 372.1343; found, 372.1345.

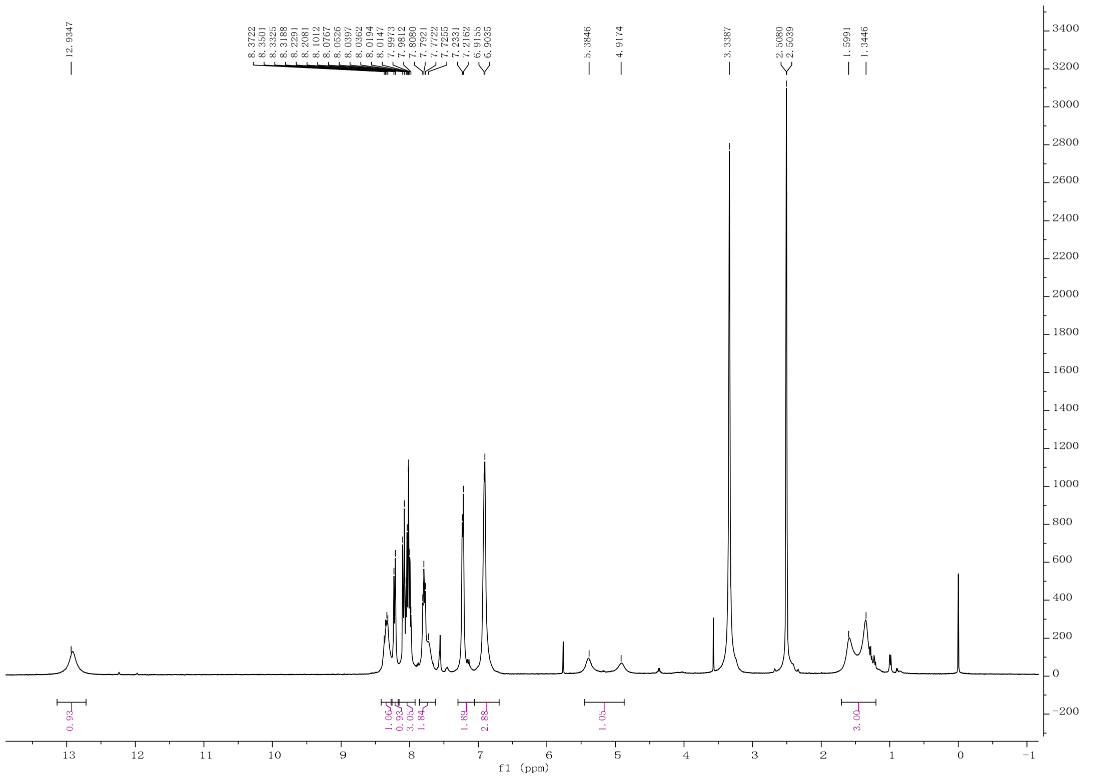

The ^{1}H NMR spectrogram of compound **F1**

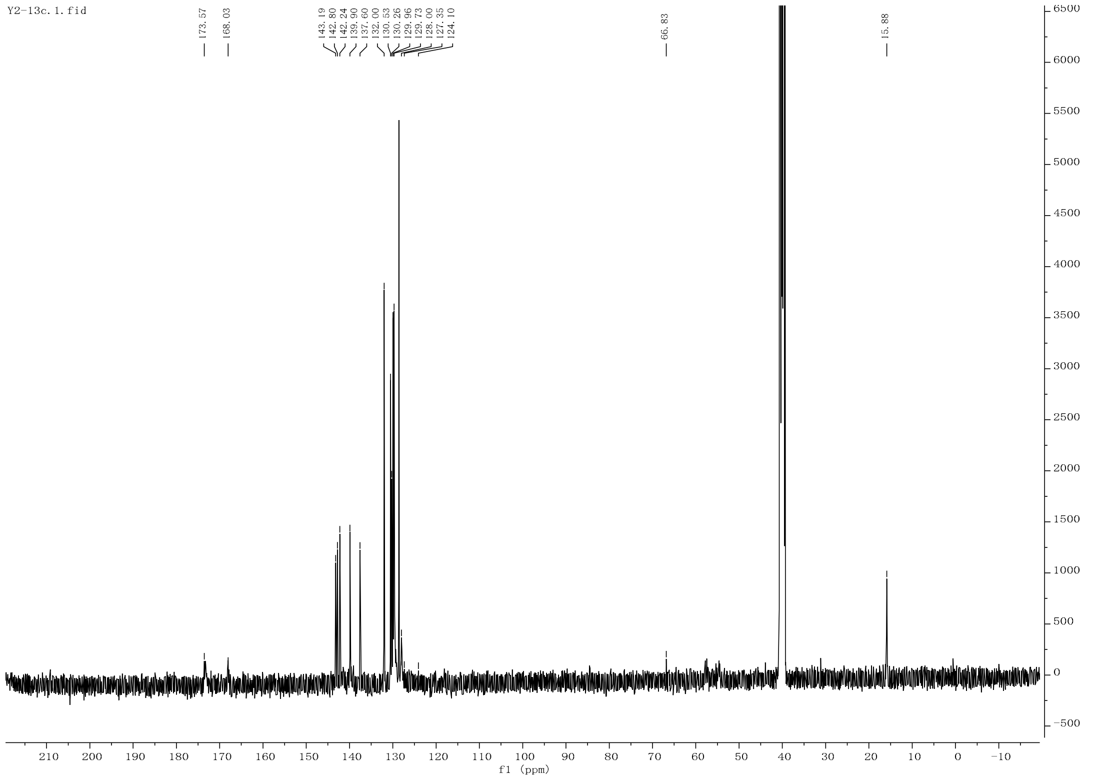

The ^{13}C NMR spectrogram of compound **F1**

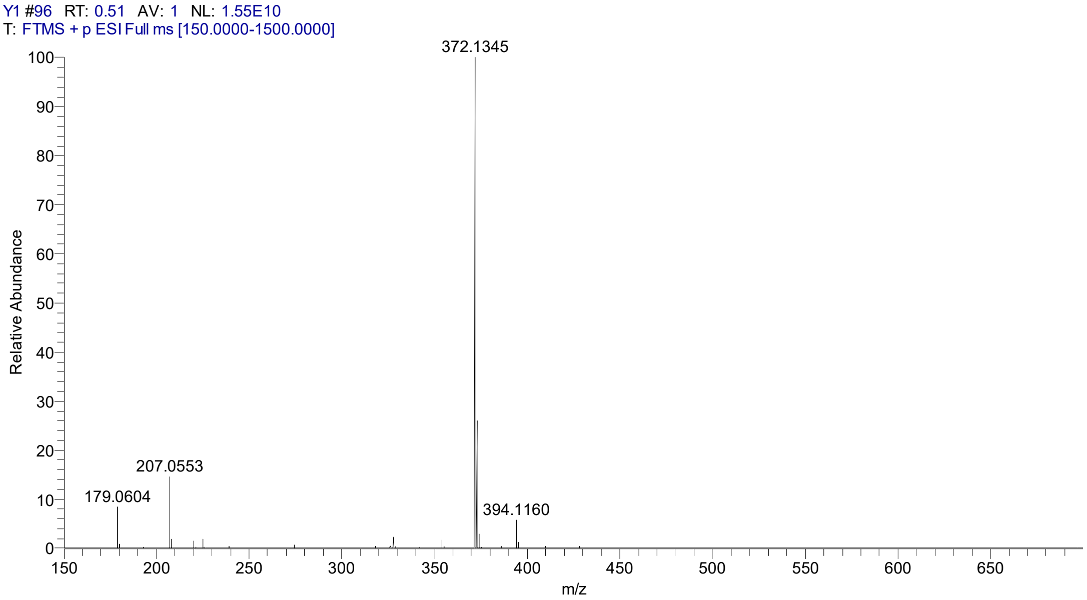

The HRMS spectrogram of compound **F1**

Compound **F2**,

N-(2-fluorophenyl)-*N*-(phenazine-1-carbonyl)alanine

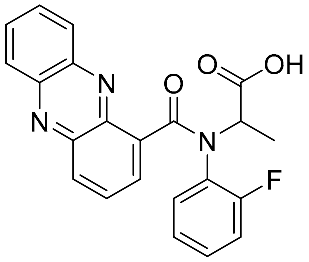

Yellow solid, yield 78.9%, m.p. 108.4-110.2°C;^{1}H NMR (400 MHz, DMSO-d_6) δ 12.98 (s, 1H), 8.34 (s, 1H), 8.23 (d, J = 8.3 Hz, 1H), 8.13 (d, J = 10.0 Hz, 1H), 8.08 – 7.97 (m, 2H), 7.83 (s, 2H), 7.14 (s, 1H), 7.03 (d, J = 7.8 Hz, 1H), 6.94 (d, J = 6.9 Hz, 1H), 6.77 (s, 1H), 5.17 (1H, two isomers), 1.39 (3H, two isomers). ^{13}C NMR (101 MHz, DMSO-d_6) δ 173.26, 168.68, 161.49, 159.19, （dd, $_{C=C-F}$=J= 232.3 Hz）143.16, 142.75, 142.04, 139.68, 136.84 (2C), 132.03 (2C), 131.35 (2C), 130.95, 130.76, 130.52, 129.82, 129.70, 123.96, 116.18, 55.11, 15.07. HRMS (ESI): calcd for $C_{22}H_{16}FN_3O_3$ {[M+H]$^+$}, 390.1248; found, 390.1250.

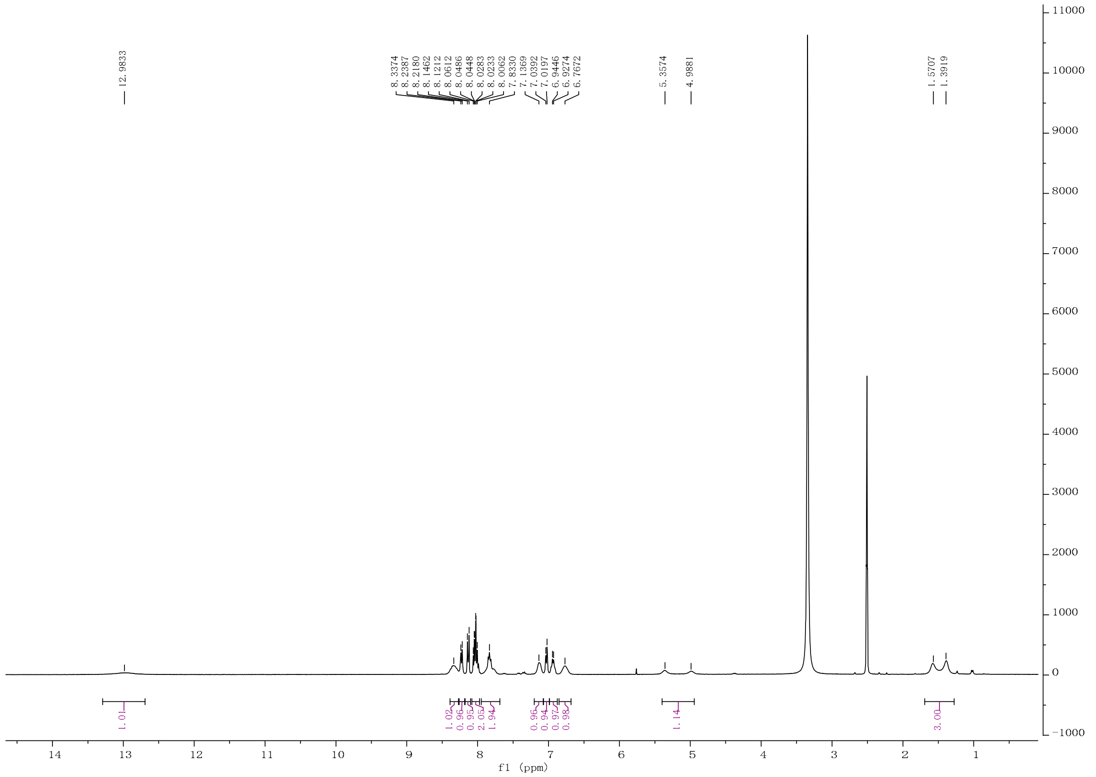

The ^{1}H NMR spectrogram of compound **F2**

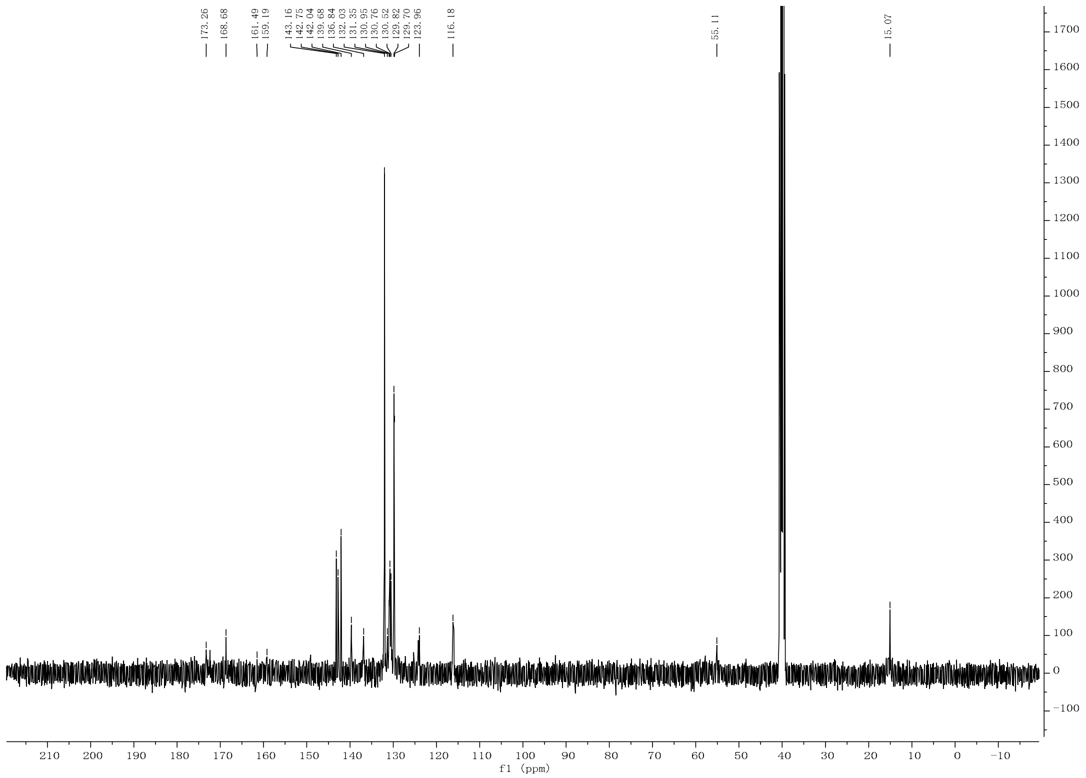

The ^{13}C NMR spectrogram of compound **F2**

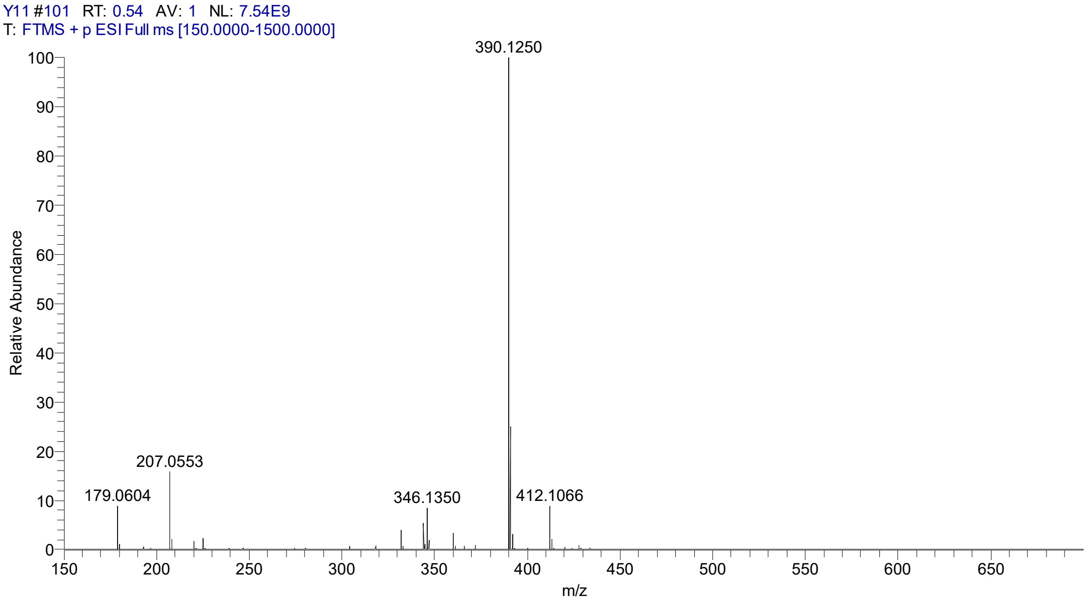

The HRMS spectrogram of compound **F2**

Compound **F3**,

N-(3-fluorophenyl)-*N*-(phenazine-1-carbonyl)alanine

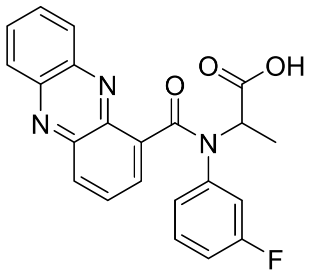

Yellow solid, yield 80.1%, m.p. 106.2-107.1°C; ^{1}H NMR (400 MHz, DMSO-d_6) δ 12.96 (s, 1H), 8.33 (dd, J = 17.2, 9.4 Hz, 1H), 8.21 (d, J = 8.3 Hz, 1H), 8.12 (d, J = 10.0 Hz, 1H), 8.05 – 7.97 (m, 2H), 7.93 – 7.70 (m, 2H), 7.12 (s, 1H), 7.02 (d, J = 7.8 Hz, 1H), 6.92 (q, J = 7.1 Hz, 1H), 6.74 (s, 1H), 5.17 (1H, two isomers), 1.47 (3H, two isomers). ^{13}C NMR (101 MHz, DMSO-d_6) δ 173.16, 168.00, 162.63, 160.19, (dd, C=C-F=J = 246.44 Hz) 143.22, 142.79, 142.21, 139.74, 137.20, 132.09, 130.59 (2C), 130.00 (2C), 129.88 (2C), 129.74 (2C), 126.38, 116.92, 115.15, 57.71, 15.86. HRMS (ESI): calcd for $C_{22}H_{16}FN_3O_3$ {[M+H]$^+$}, 390.1248; found, 390.1248.

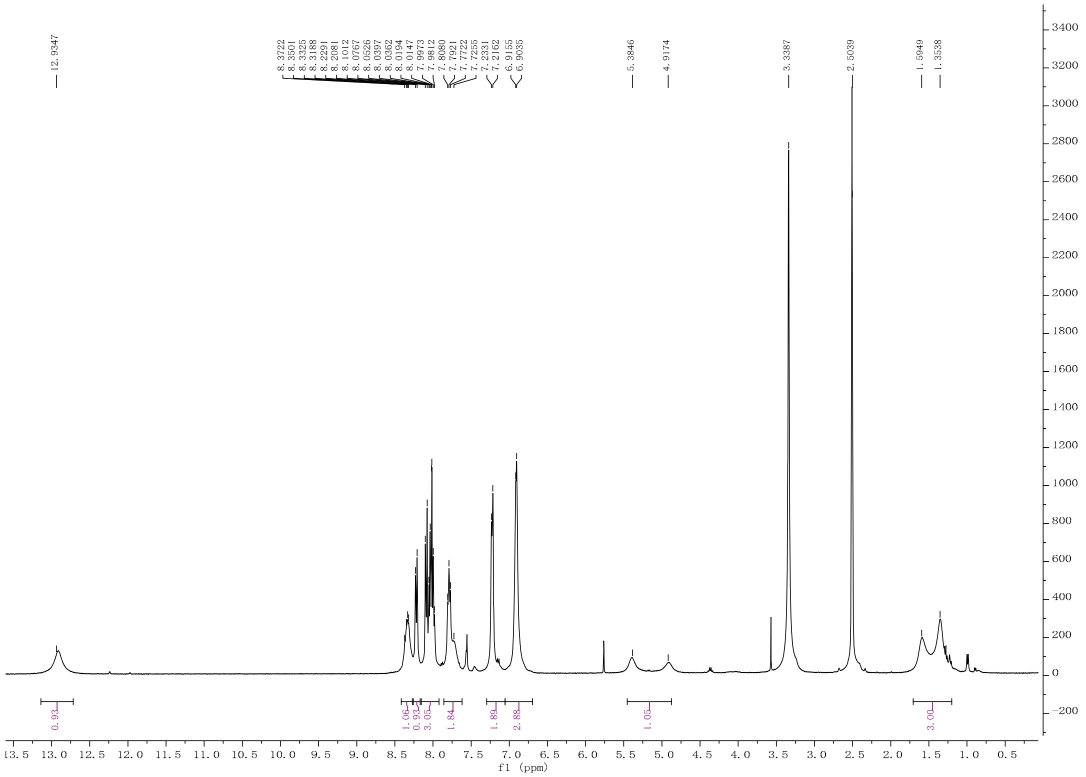

The ^{1}H NMR spectrogram of compound **F3**

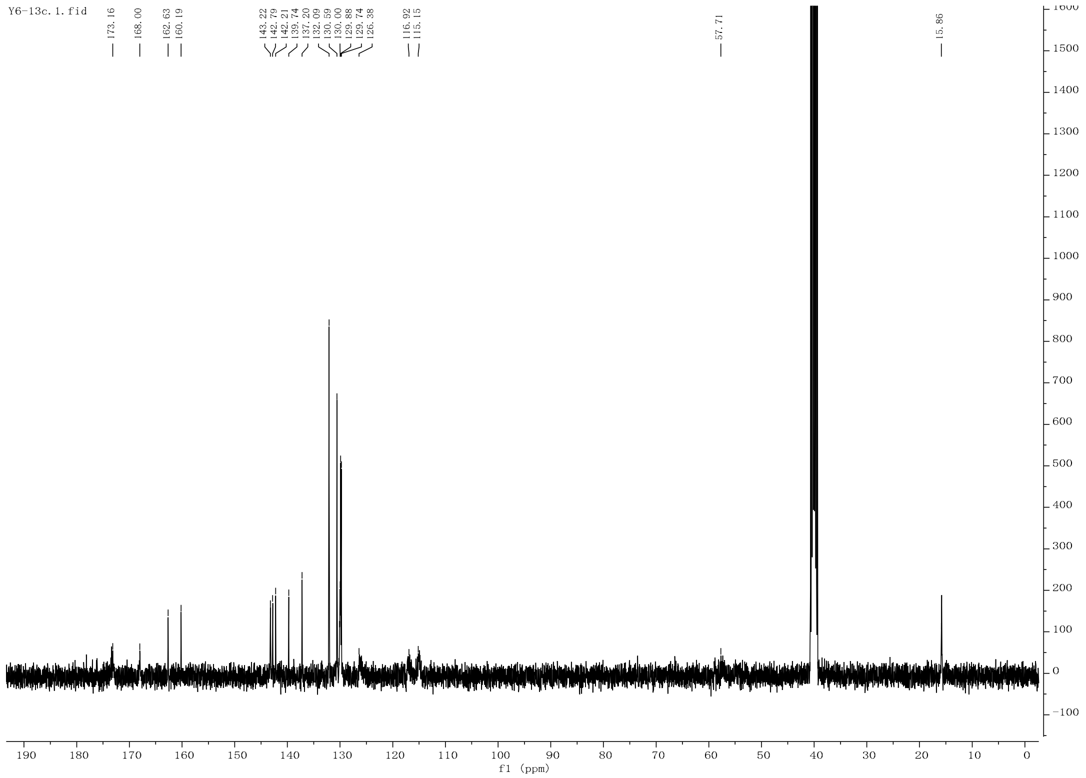

The ^{13}C NMR spectrogram of compound **F3**

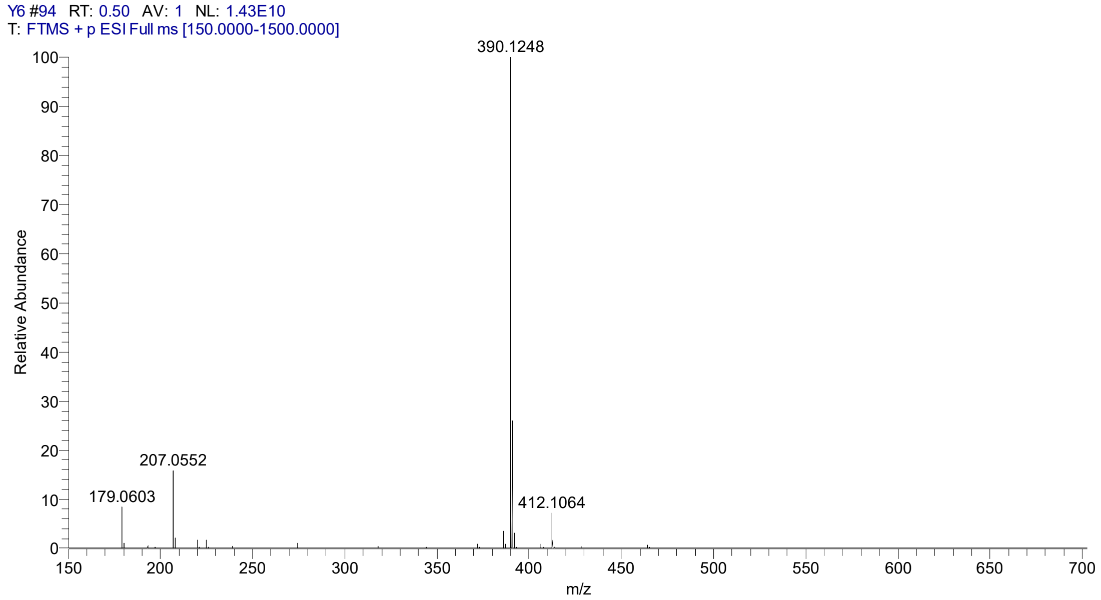

The HRMS spectrogram of compound **F3**

Compound **F4**,

N-(4-fluorophenyl)-*N*-(phenazine-1-carbonyl)alanine

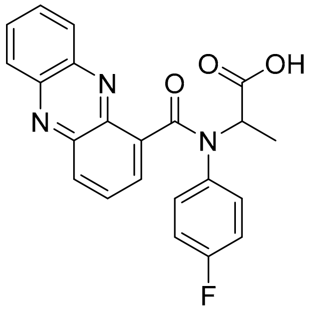

Yellow solid, yield 80.5%, m.p. 80.1-81.4°C; ^{1}H NMR (400 MHz, DMSO-d_6) δ 12.97 (s, 1H), 8.39 – 8.27 (m, 1H), 8.26 – 8.18 (m, 1H), 8.12 (d, J = 10.1 Hz, 1H), 8.01 (dddd, J = 14.4, 8.1, 6.6, 1.6 Hz, 2H), 7.81 (dd, J = 8.7, 6.6 Hz, 2H), 7.43 – 7.17 (m, 2H), 6.76 (t, J = 8.0 Hz, 2H), 5.17 (1H, two isomers), 1.45 (3H, two isomers). ^{13}C NMR (101 MHz, DMSO-d_6) δ 173.14, 168.27, 162.36, 159.91, (dd, $_{C=C-F}$=J = 247.45 Hz) 143.26, 142.83, 142.25, 139.75, 137.46, 132.04, 130.55, 130.37, 129.95, 129.74, 129.71, 115.50, 115.28, 54.59, 15.88. HRMS (ESI): calcd for $C_{22}H_{16}FN_3O_3$ {[M+H]$^+$}, 390.1249; found, 390.1252.

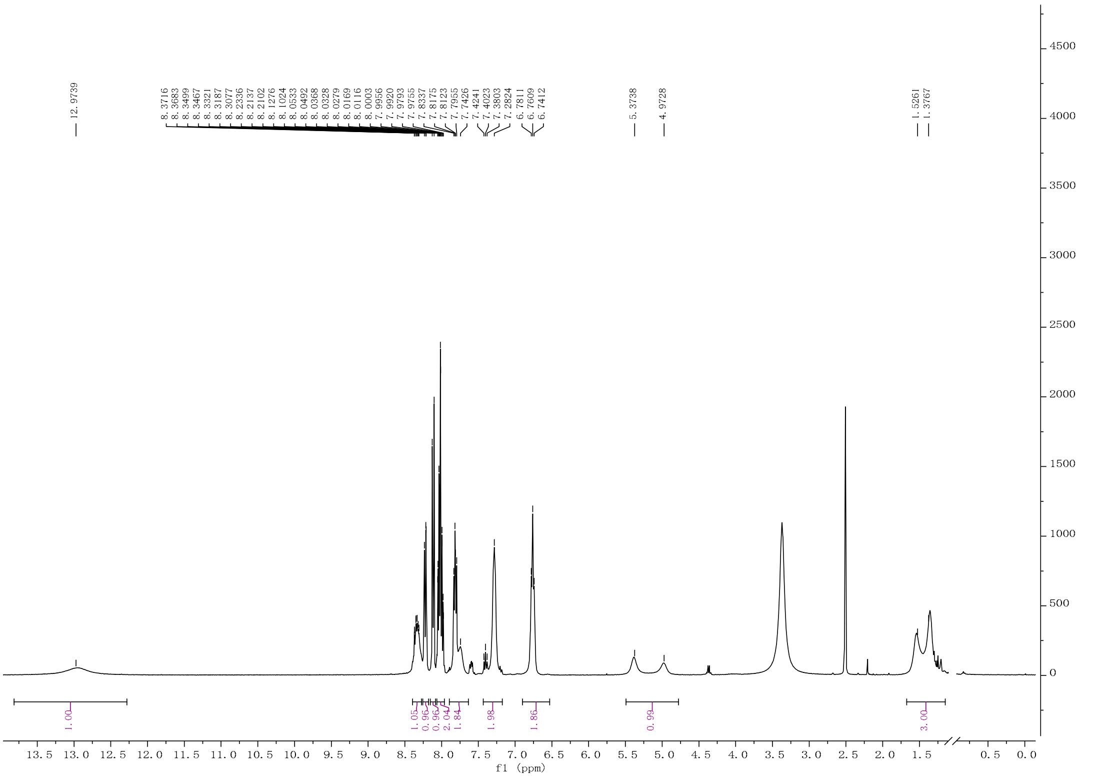

The ^{1}H NMR spectrogram of compound **F4**

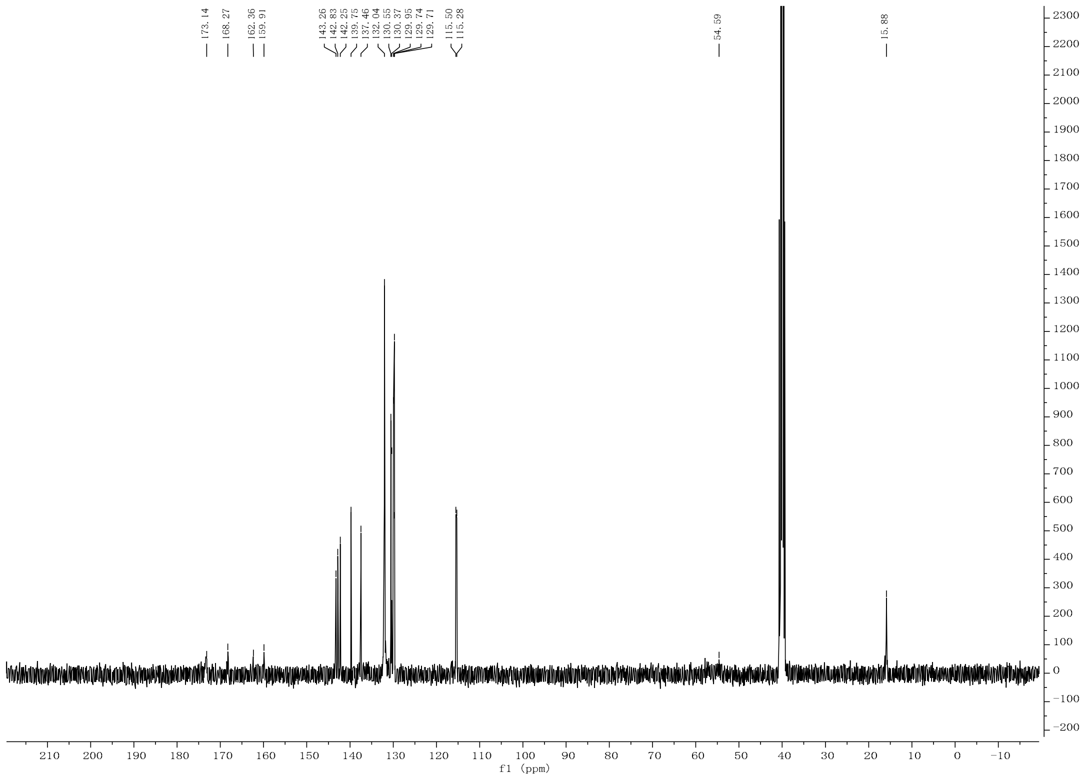

The ^{13}C NMR spectrogram of compound **F4**

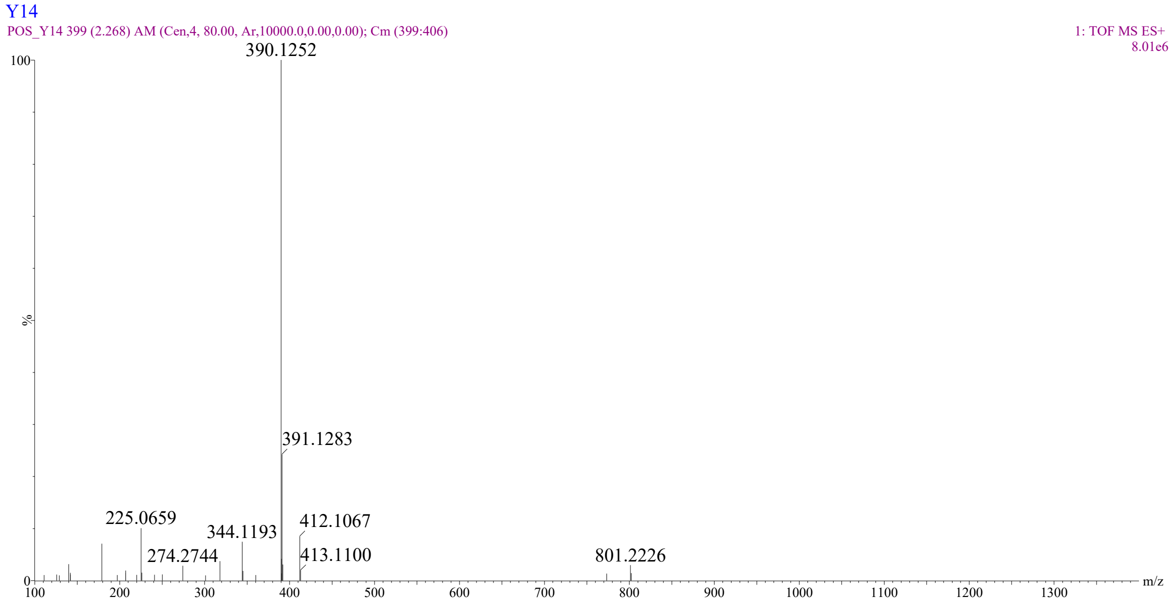

The HRMS spectrogram of compound **F4**

Compound **F5**,

N-(2-chlorophenyl)-*N*-(phenazine-1-carbonyl)alanine

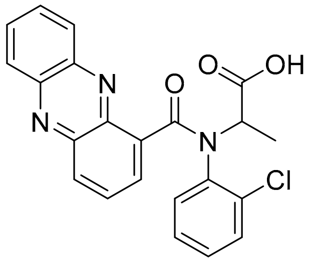

Yellow solid, yield 81.6%, m.p. 103.4-104.8℃; ^{1}H NMR (400 MHz, DMSO-d_6) δ 12.93 (s, 1H), 8.31 (d, J = 7.7 Hz, 1H), 8.21 (d, J = 8.2 Hz, 1H), 8.14 – 8.10 (m, 1H), 7.99 (ddd, J = 24.5, 15.5, 7.7 Hz, 4H), 7.82 (q, J = 8.5 Hz, 1H), 7.71 (d, J = 8.3 Hz, 1H), 7.19 (s, 1H), 7.00 – 6.72 (m, 2H), 5.15 (s, 1H), 1.22 (d, J = 7.2 Hz, 3H). ^{13}C NMR (101 MHz, DMSO-d_6) δ 173.48, 168.58, 143.14, 142.70, 142.06, 139.85, 136.71, 132.05, 130.97, 130.78, 130.50, 130.42, 130.34, 130.07, 129.91, 129.83, 129.74, 128.51, 127.22, 55.27, 14.93. HRMS (ESI): calcd for $C_{22}H_{16}ClN_3O_3$ {[M+H]$^+$}, 406.0953; found, 406.0955.

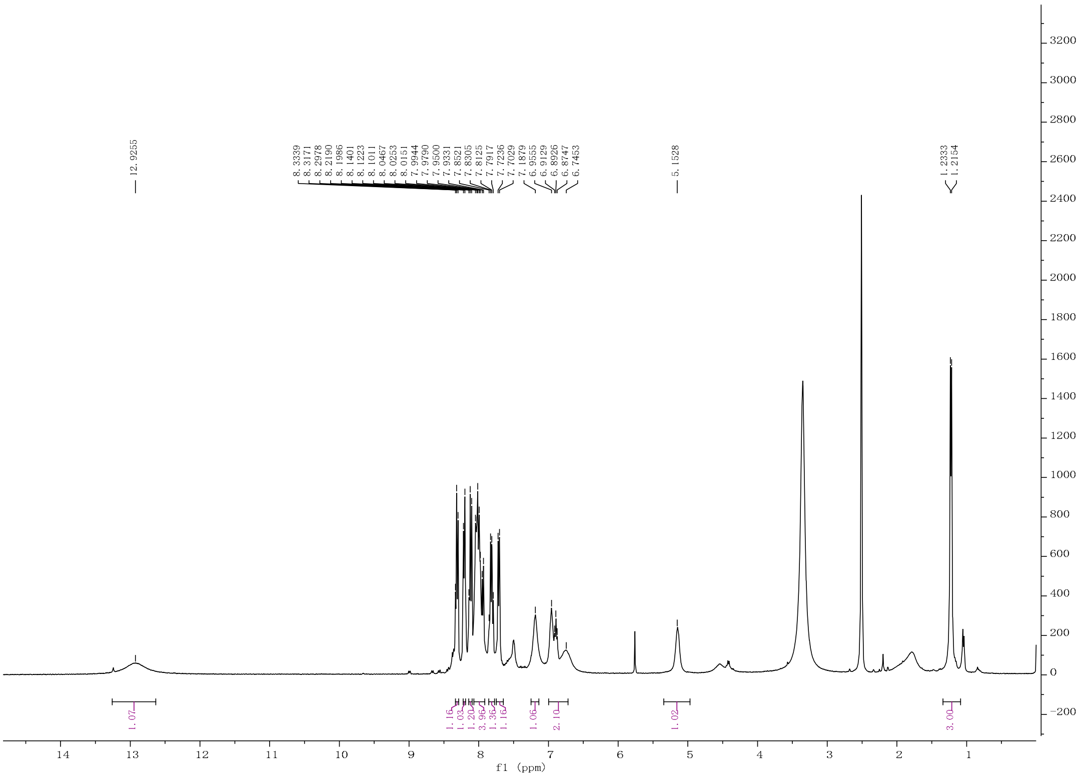

The ^{1}H NMR spectrogram of compound **F5**

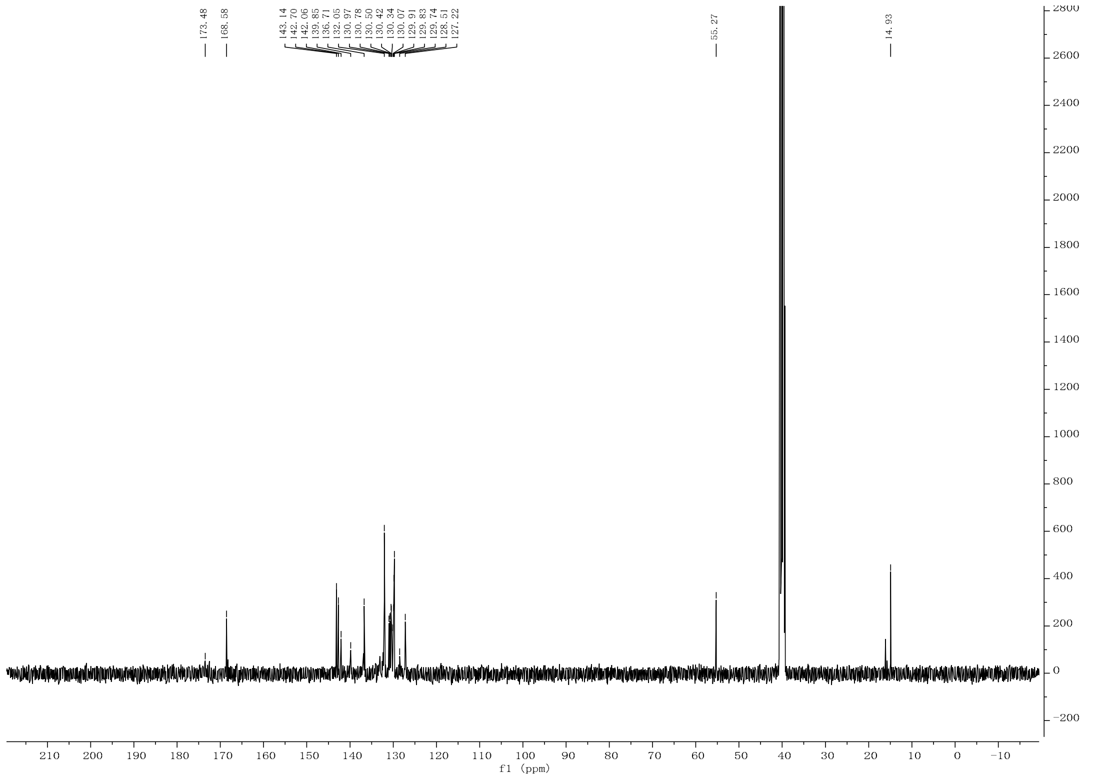

The ^{13}C NMR spectrogram of compound **F5**

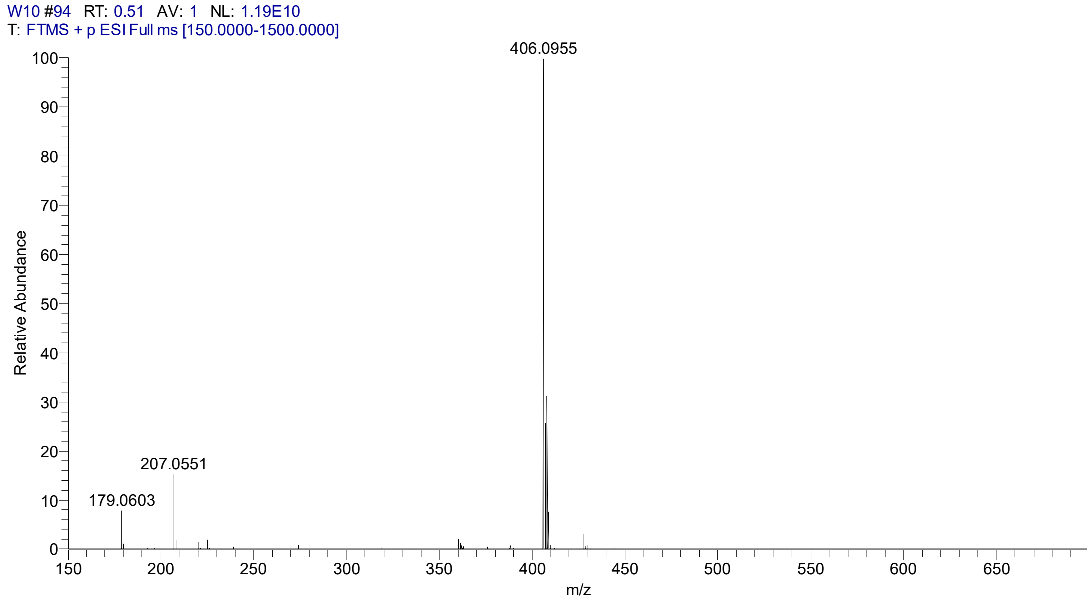

The HRMS spectrogram of compound **F5**

Compound **F6**,

N-(3-chlorophenyl)-*N*-(phenazine-1-carbonyl)alanine

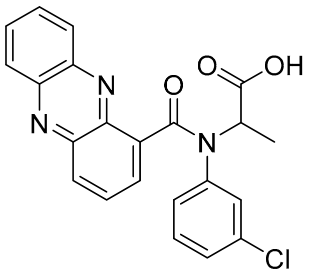

White solid, yield 79.9%, m.p. 118.3-119.4°C; ^{1}H NMR (400 MHz, DMSO-d_6) δ 13.01 (s, 1H), 8.35 (dd, J = 19.3, 6.8 Hz, 1H), 8.22 (d, J = 8.2 Hz, 1H), 8.14 (d, J = 9.9 Hz, 1H), 8.06 – 7.97 (m, 2H), 7.94 – 7.70 (m, 2H), 7.38 (s, 1H), 7.15 (d, J = 7.1 Hz, 1H), 7.02 – 6.83 (m, 2H), 5.18 (1H, two isomers), 1.48 (3H, two isomers). ^{13}C NMR (101 MHz, DMSO-d_6) δ 173.48, 167.98, 143.22, 142.77, 142.19, 139.61, 137.19, 132.47, 132.10, 132.06, 130.65, 130.58, 129.97, 129.88, 129.72, 128.07, 125.00, 119.51, 118.24, 66.84, 15.86. HRMS (ESI): calcd for $C_{22}H_{16}ClN_3O_3$ {[M+H]$^+$}, 406.0953; found, 406.0958.

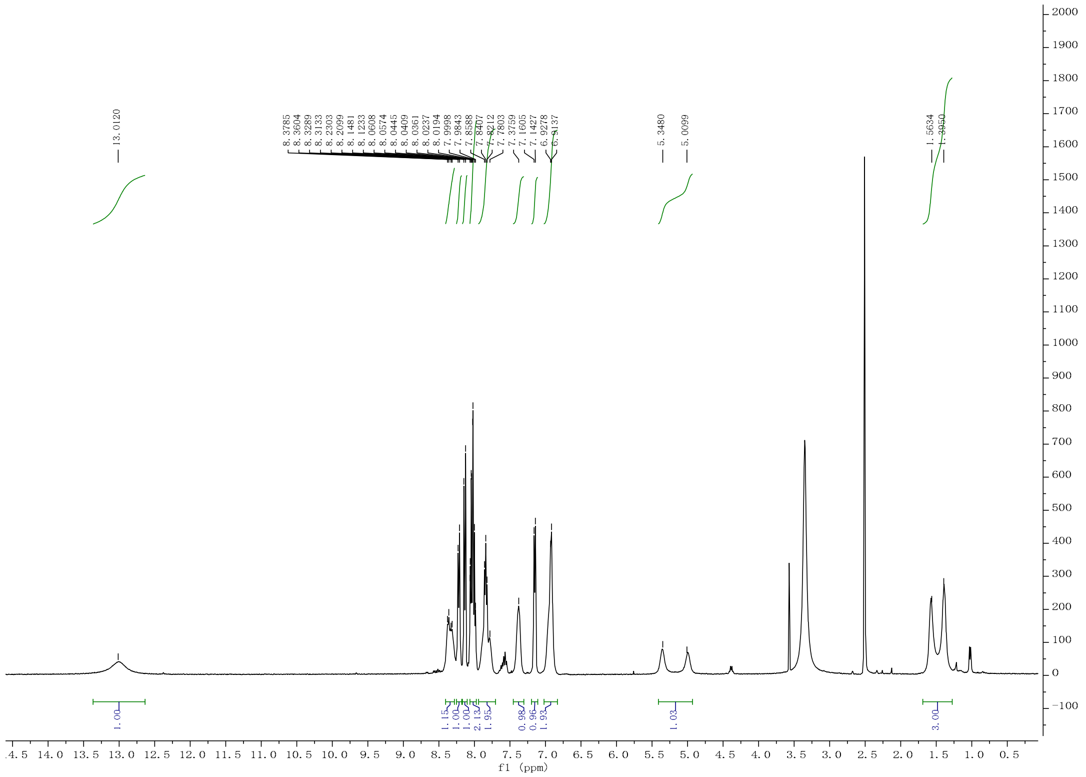

The ^{1}H NMR spectrogram of compound **F6**

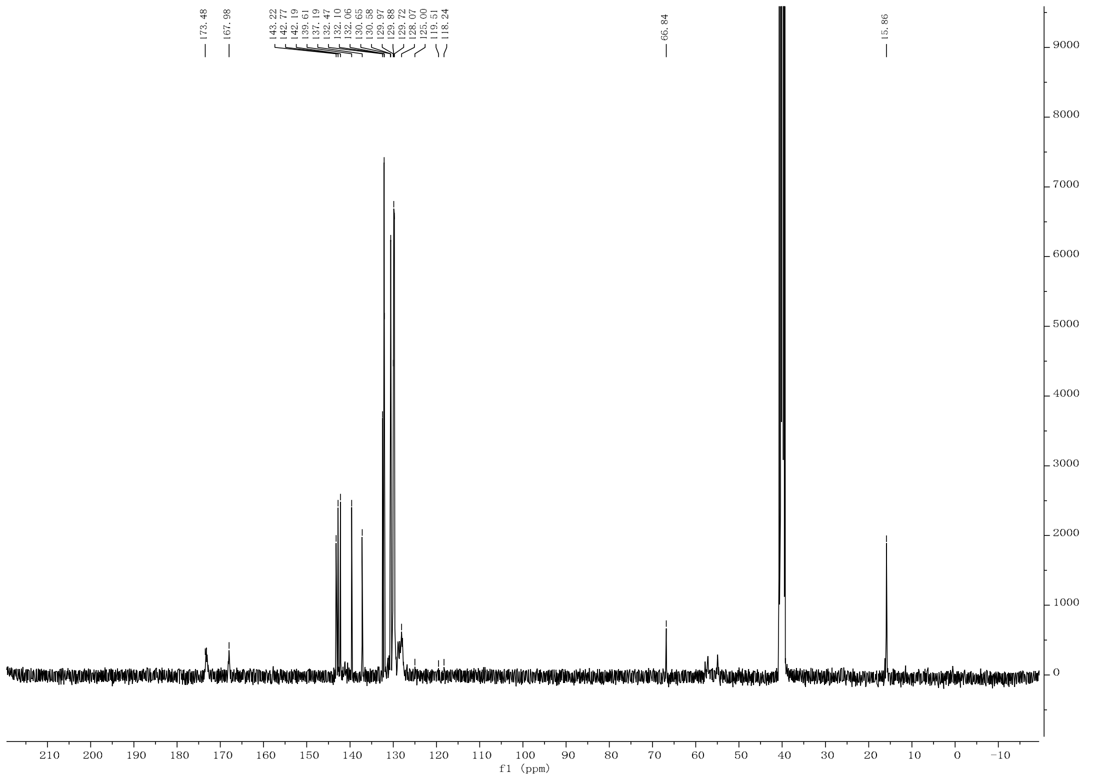

The ^{13}C NMR spectrogram of compound **F6**

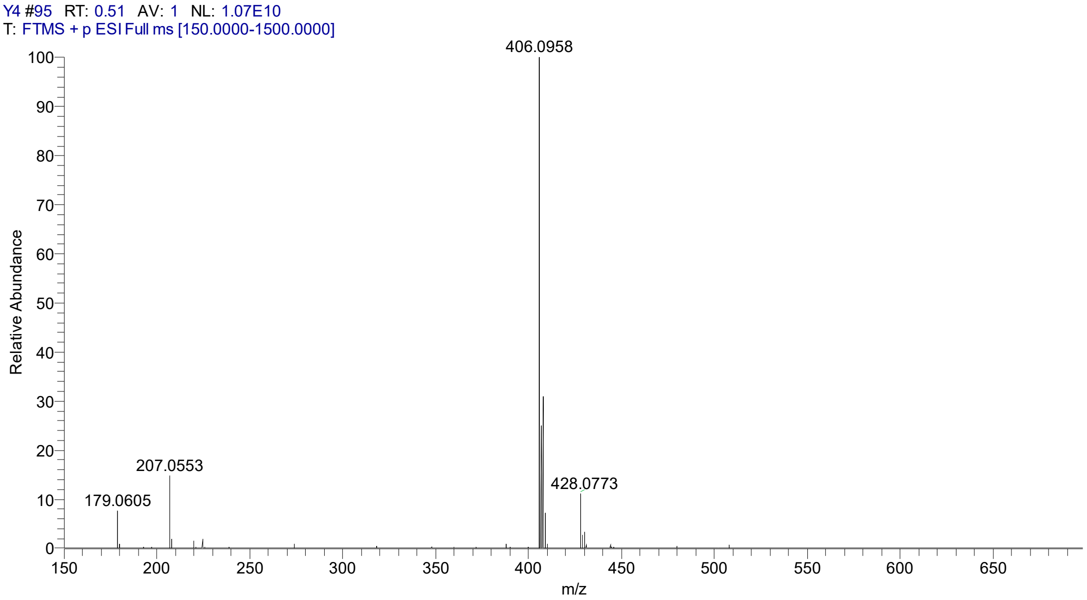

The HRMS spectrogram of compound **F6**

Compound **F7**,

N-(4-chlorophenyl)-*N*-(phenazine-1-carbonyl)alanine

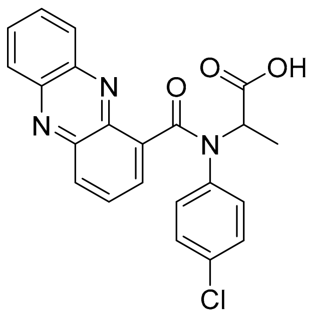

Yellow solid, yield 80.3%, m.p. 93.2-94.9°C; ^{1}H NMR (400 MHz, DMSO-d_6) δ 12.99 (s, 1H), 8.39 – 8.27 (m, 1H), 8.22 (d, *J* = 8.3 Hz, 1H), 8.13 (d, *J* = 10.0 Hz, 1H), 8.02 (dtd, *J* = 12.7, 6.5, 3.5 Hz, 2H), 7.90 – 7.65 (m, 2H), 7.34 – 7.15 (m, 2H), 7.00 (d, *J* = 7.4 Hz, 2H), 5.16 (1H, two isomers), 1.46 (3H, two isomers). ^{13}C NMR (101 MHz, DMSO-d_6) δ 173.51, 168.02, 143.28, 142.86, 142.25, 139.78, 137.29, 132.06, 130.56 (2C), 130.51 (2C), 129.96 (4C), 129.75, 128.67, 121.70 (2C), 57.44, 15.88. HRMS (ESI): calcd for $C_{22}H_{16}ClN_3O_3$ {[M+H]$^+$}, 406.0953; found, 406.0959.

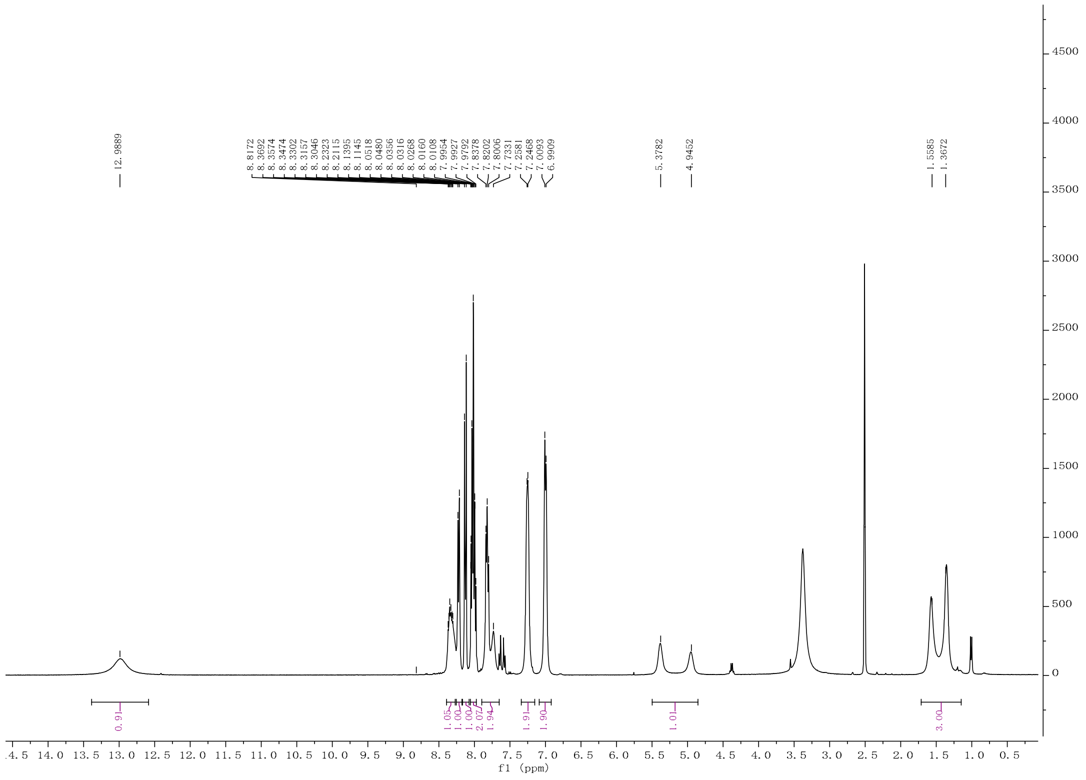

The ^{1}H NMR spectrogram of compound **F7**

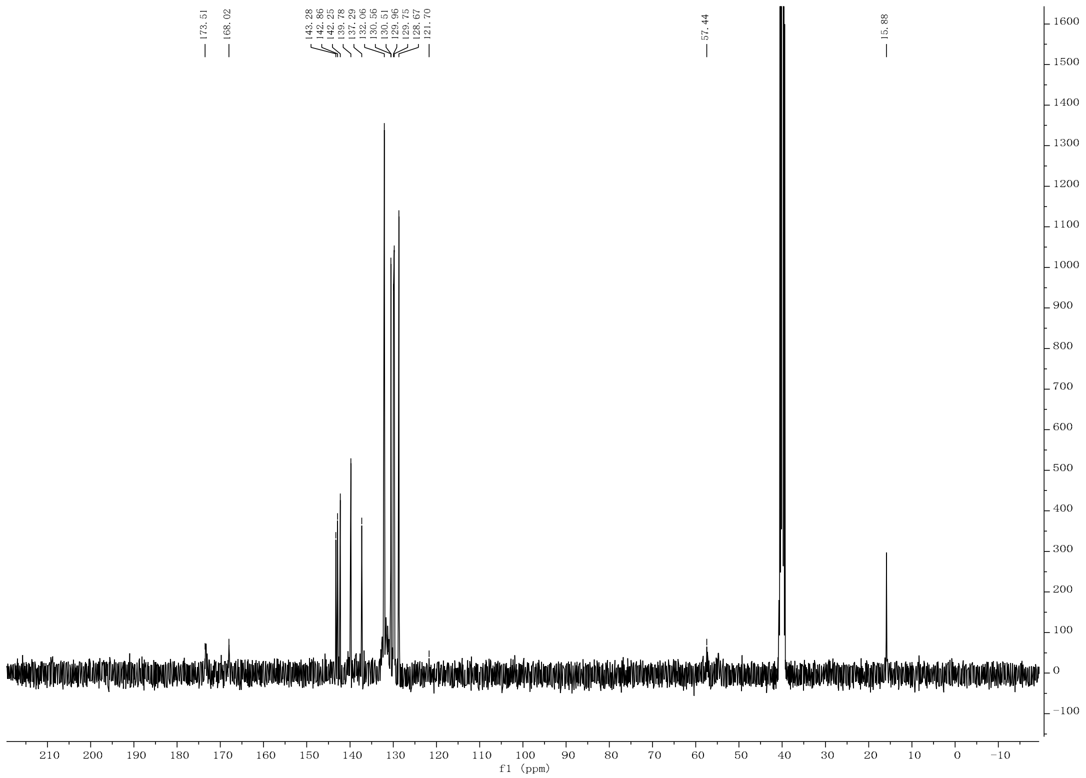

The ^{13}C NMR spectrogram of compound **F7**

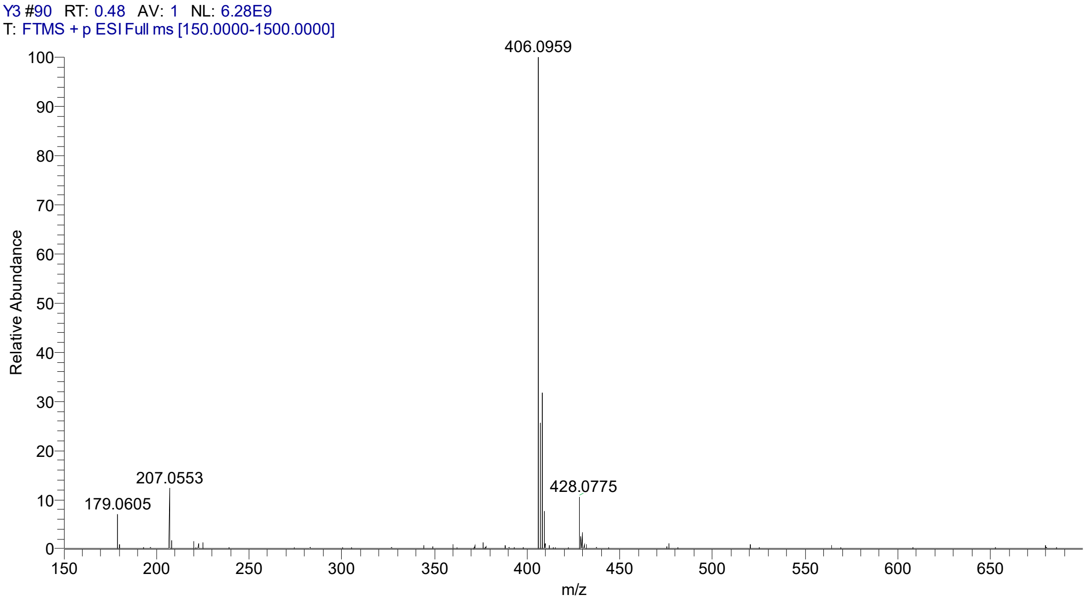

The HRMS spectrogram of compound **F7**

Compound **F8**,

N-(3-bromophenyl)-*N*-(phenazine-1-carbonyl)alanine

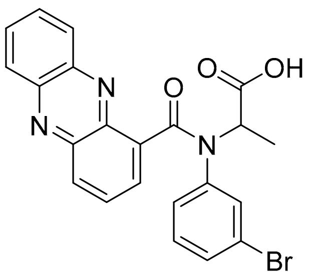

Brown solid, yield 80.2%, m.p. 116.5-117.3°C; ^{1}H NMR (400 MHz, DMSO-d_6) δ 13.00 (s, 1H), 8.34 (dd, J = 18.0, 8.6 Hz, 1H), 8.22 (d, J = 8.2 Hz, 1H), 8.13 (d, J = 9.9 Hz, 1H), 8.06 – 7.97 (m, 2H), 7.92 – 7.71 (m, 2H), 7.52 (d, J = 14.0 Hz, 1H), 7.20 (d, J = 8.0 Hz, 1H), 7.07 (s, 1H), 6.86 (d, J = 7.2 Hz, 1H), 5.17 (1H, two isomers), 1.48 (3H, two isomers). ^{13}C NMR (101 MHz, DMSO-d_6) δ 173.37, 167.89, 143.22, 142.78, 142.19, 139.60, 137.21, 132.10 (2C), 132.05 (2C), 130.89, 130.66 (2C), 130.56, 130.22, 129.91, 129.70, 120.67 (2C), 54.87, 15.88. HRMS (ESI): calcd for $C_{22}H_{16}BrN_3O_3$ {$[M+H]^+$}, 450.0448; found, 450.0451.

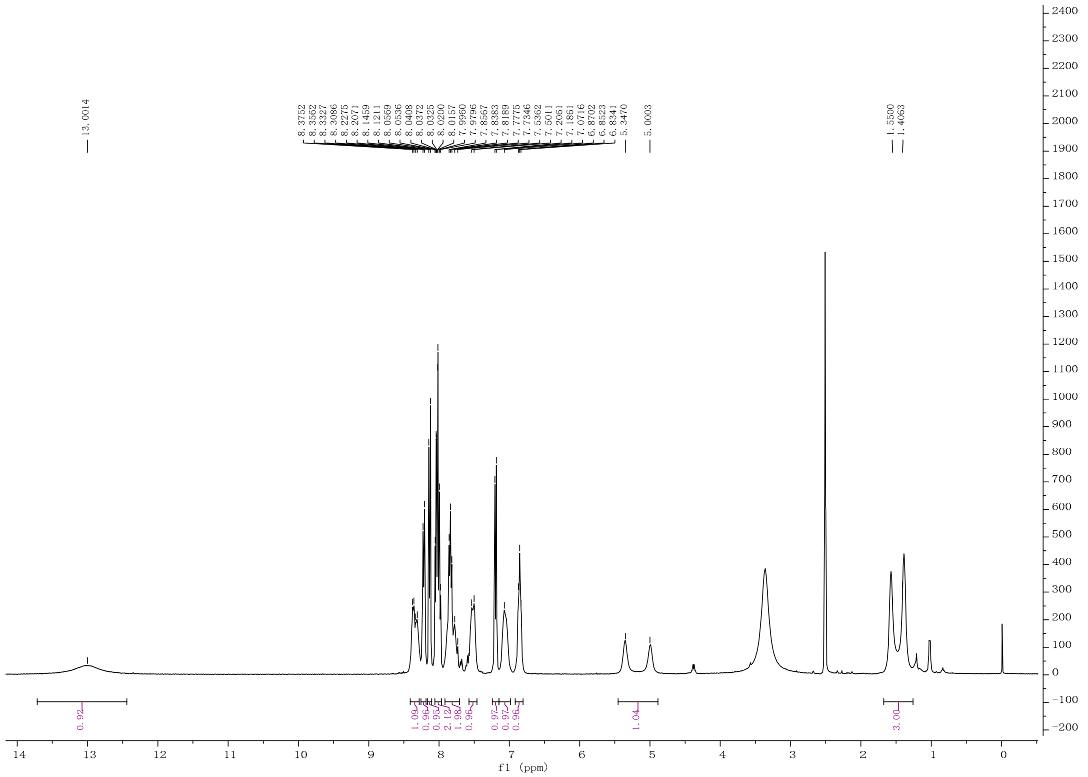

The ^{1}H NMR spectrogram of compound **F8**

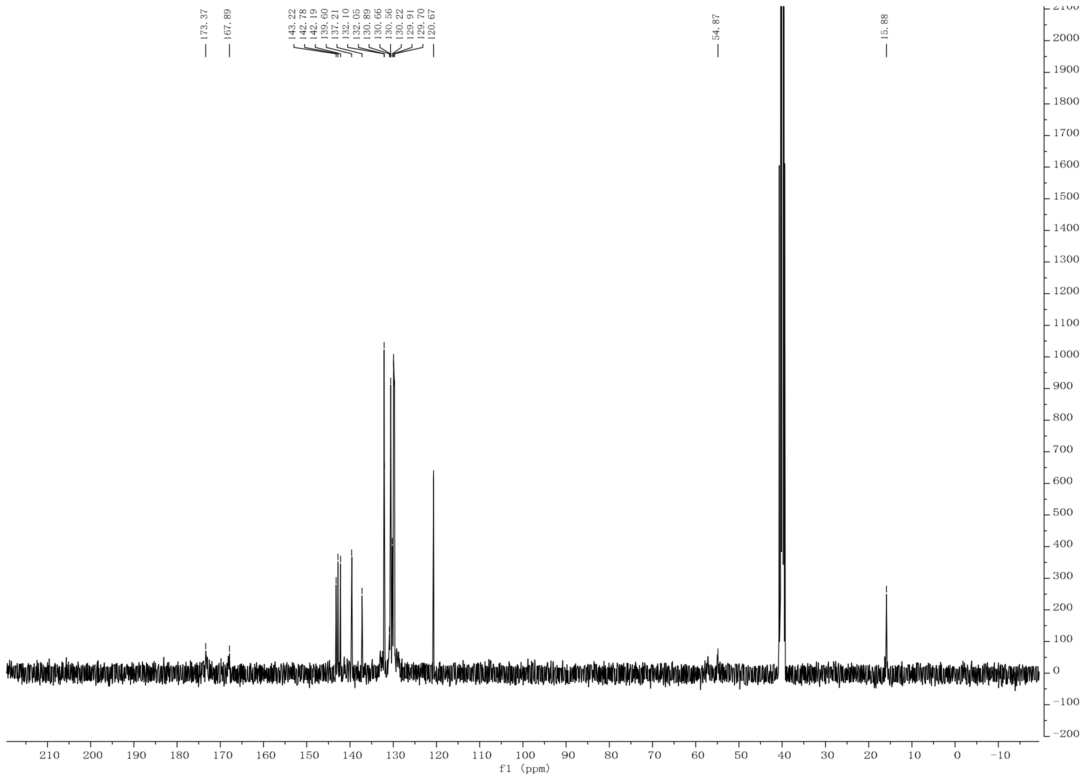

The ^{13}C NMR spectrogram of compound **F8**

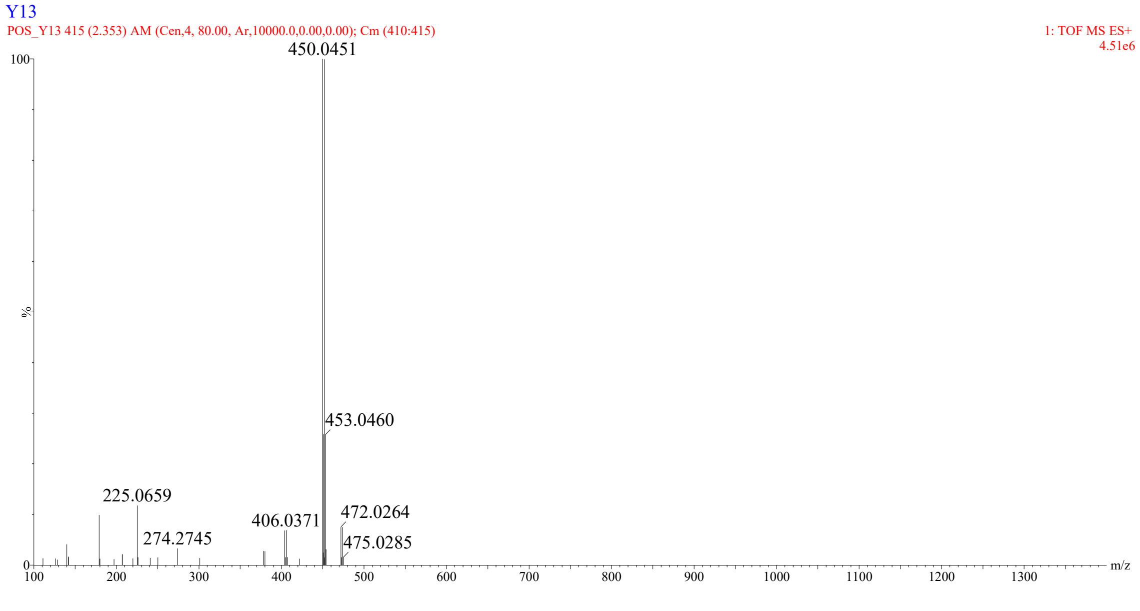

The HRMS spectrogram of compound **F8**

Compound **F9**,

N-(4-bromophenyl)-*N*-(phenazine-1-carbonyl)alanine

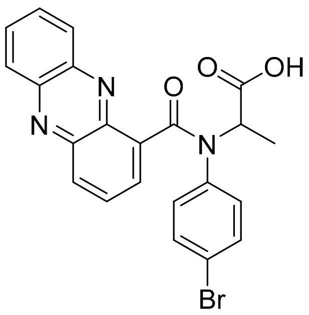

Brownness solid, yield 77.9%, m.p. 111.3-112.5℃;^{1}H NMR (400 MHz, DMSO-d_6) δ 12.98 (s, 1H), 8.33 (dd, J = 16.4, 9.2 Hz, 1H), 8.22 (d, J = 8.3 Hz, 1H), 8.12 (d, J = 9.9 Hz, 1H), 8.05 – 7.96 (m, 2H), 7.85 – 7.69 (m, 2H), 7.15 (d, J = 12.7 Hz, 4H), 5.16 (1H, two isomers), 1.46 (3H, two isomers). ^{13}C NMR (101 MHz, DMSO-d_6) δ 172.98, 168.05, 143.29, 142.86, 142.23, 139.79, 137.24, 132.10, 131.68, 130.59, 130.53, 129.97, 129.87, 129.75, 121.28, 55.38, 15.92. HRMS (ESI): calcd for $C_{22}H_{16}BrN_3O_3$ {[M+H]$^+$}, 450.0448; found, 450.0451.

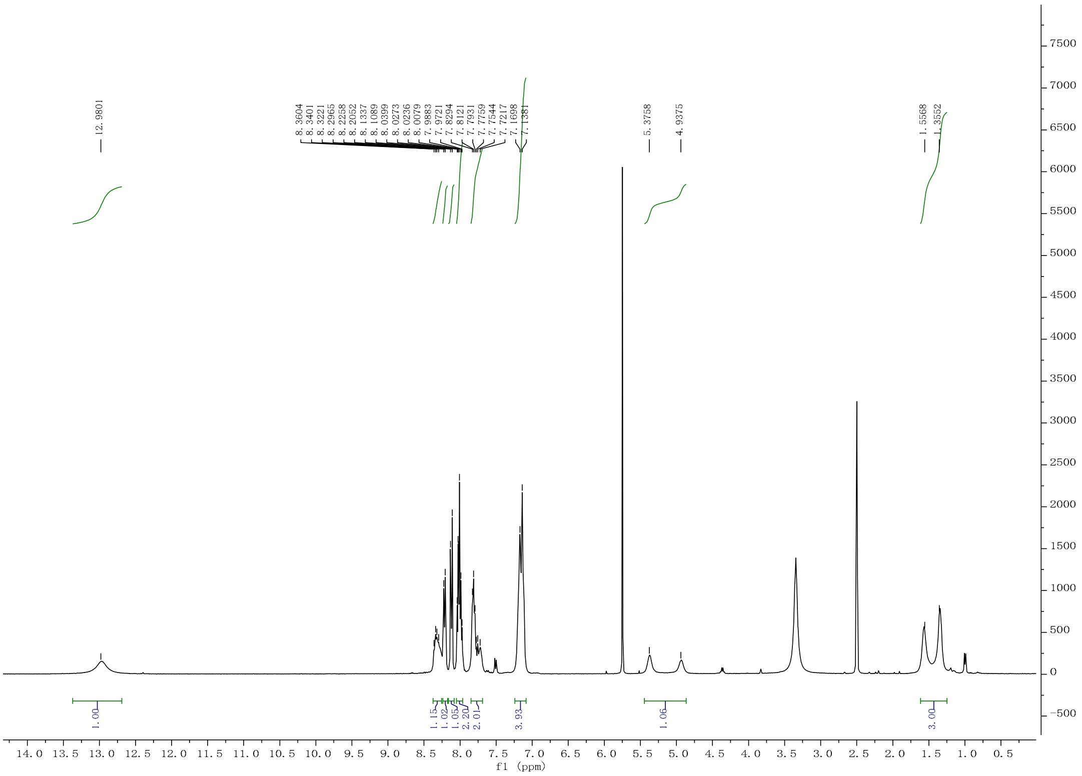

The ^{1}H NMR spectrogram of compound **F9**

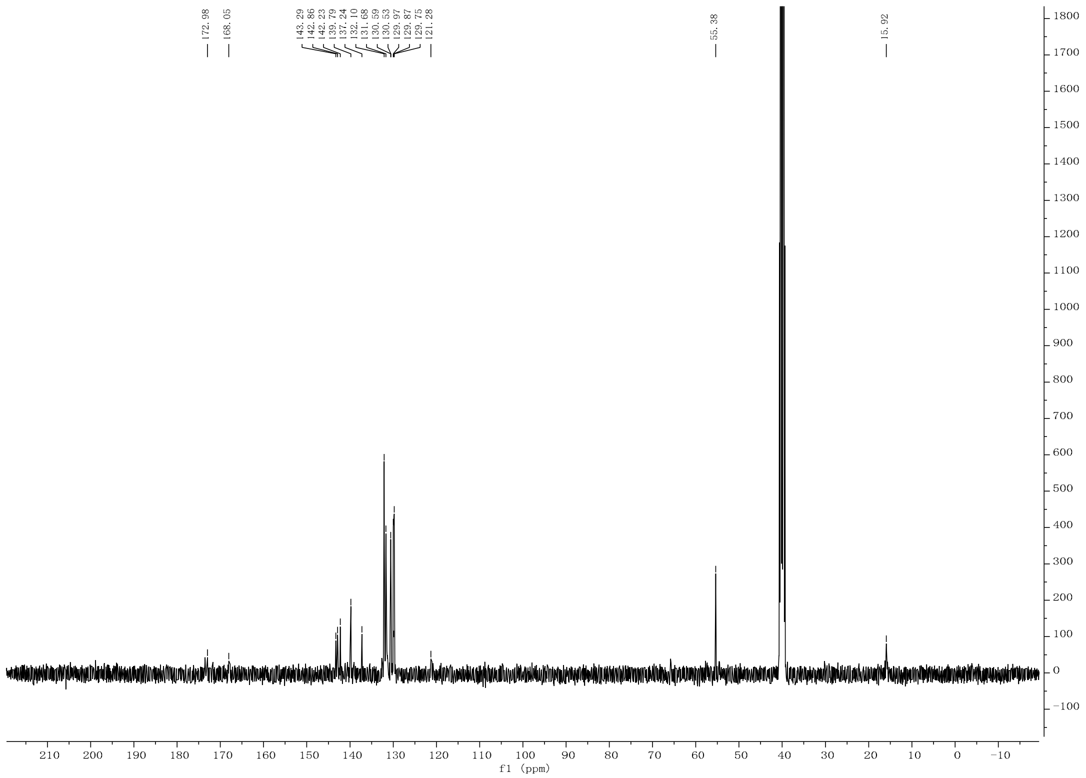

The ^{13}C NMR spectrogram of compound **F9**

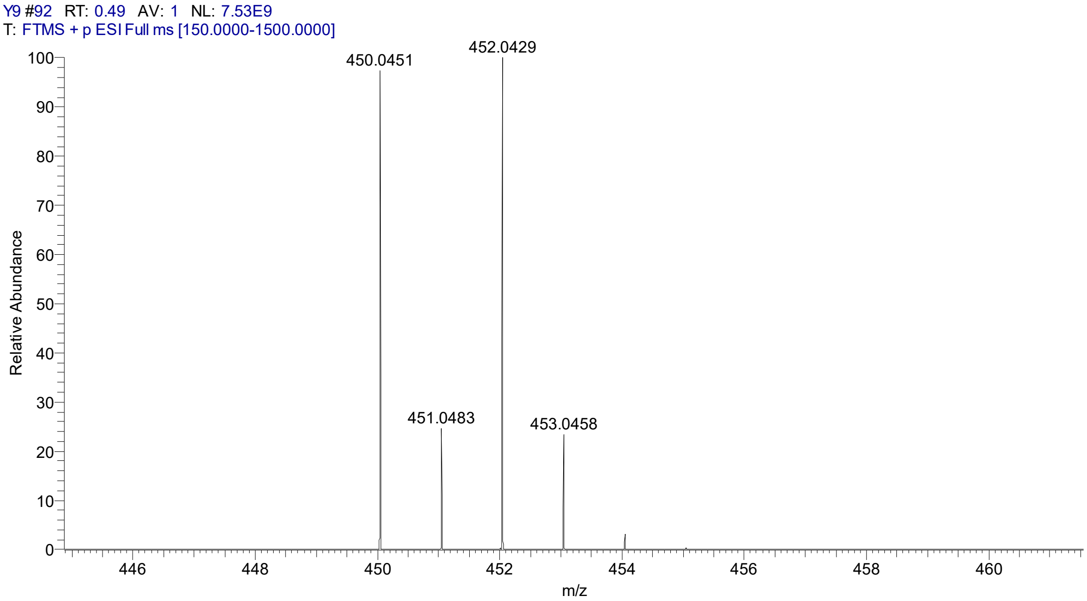

The HRMS spectrogram of compound **F9**

Compound **F10**,

N-(phenazine-1-carbonyl)-*N*-(m-tolyl)alanine

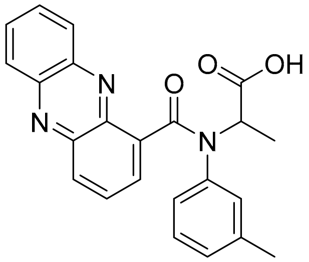

Purple solid, yield 80.2%, m.p. 94.2-95.3°C; ^{1}H NMR (400 MHz, DMSO-d_6) δ 8.39 – 8.30 (m, 1H), 8.21 (d, J = 8.3 Hz, 1H), 8.10 – 7.97 (m, 3H), 7.80 (s, 2H), 7.12 (s, 1H), 6.95 (d, J = 7.7 Hz, 1H), 6.75 (d, J = 7.4 Hz, 2H), 5.11 (1H, two isomers), 1.81 (s, 3H), 1.48 (3H, two isomers). ^{13}C NMR (101 MHz, DMSO-d_6) δ 173.67, 168.26, 143.12, 142.73, 142.23, 139.87 (2C), 137.79, 132.04, 131.97, 131.93, 130.55, 130.24, 129.88, 129.70, 129.63, 128.21, 127.02, 119.95, 119.02, 57.86, 54.65, 20.80, 15.87. HRMS (ESI): calcd for $C_{23}H_{19}N_3O_3$ {$[M+H]^+$}, 386.1500; found, 386.1503.

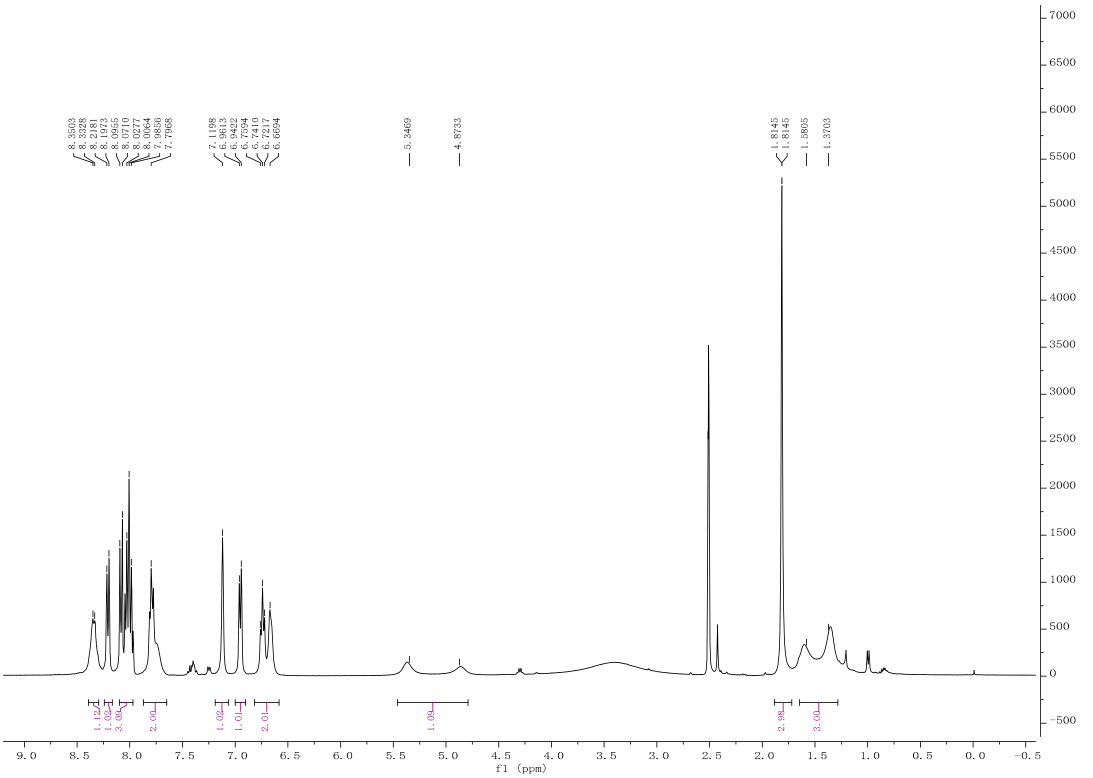

The ^{1}H NMR spectrogram of compound **F10**

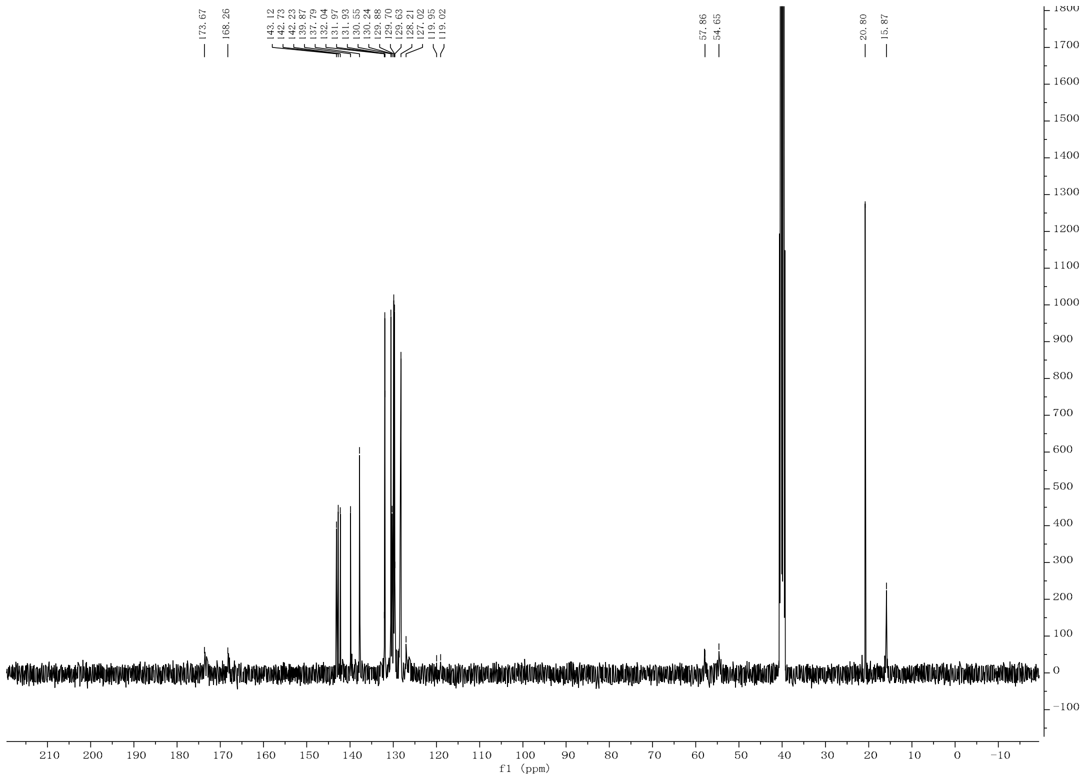

The ^{13}C NMR spectrogram of compound **F10**

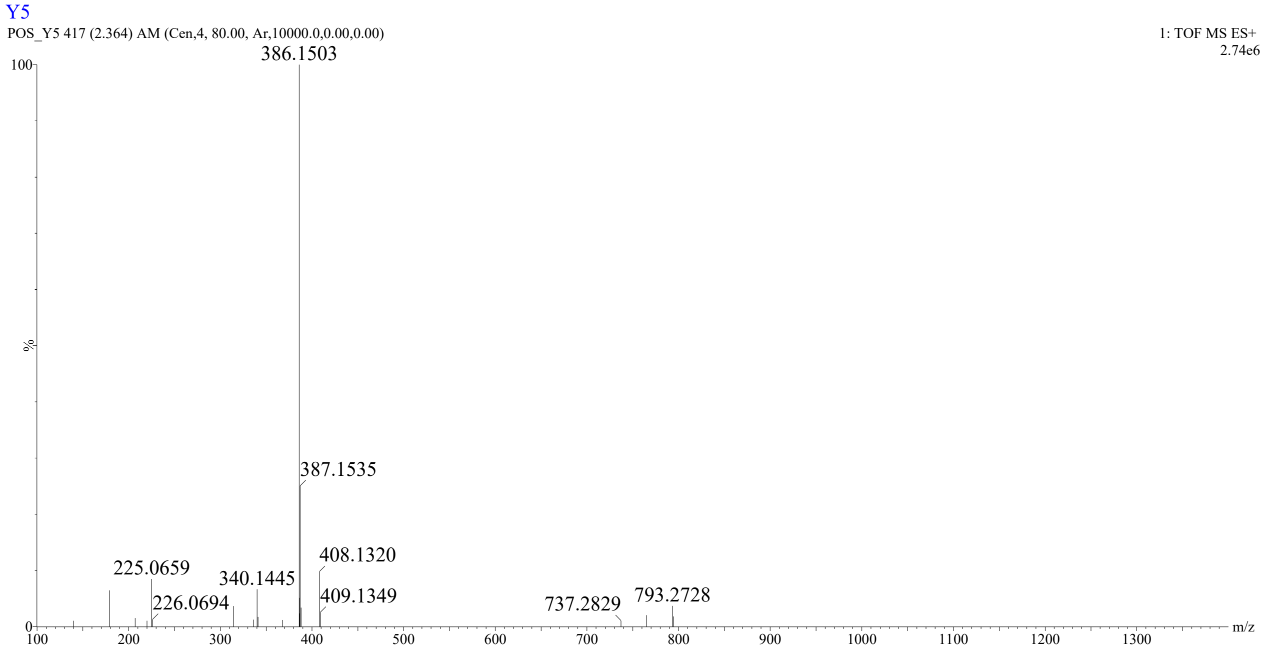

The HRMS spectrogram of compound **F10**

Compound **F11**,

N-(3-ethylphenyl)-*N*-(phenazine-1-carbonyl)alanine

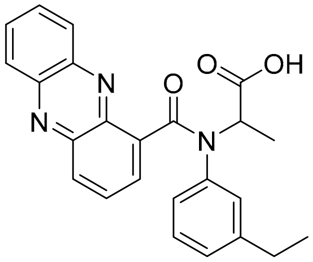

Yellow solid, yield 81.1%, m.p. 94.5-96.4°C; ^{1}H NMR (400 MHz, DMSO-d_6) δ 12.88 (s, 1H), 8.35 (s, 1H), 8.21 (d, J = 8.3 Hz, 1H), 8.10 – 7.96 (m, 3H), 7.85 – 7.66 (m, 2H), 7.02 (d, J = 7.1 Hz, 2H), 6.75 (d, J = 58.9 Hz, 2H), 5.38 (1H, two isomers), 2.07 (q, J = 7.0 Hz, 2H), 1.48 (3H, two isomers), 0.37 (s, 3H). ^{13}C NMR (101 MHz, DMSO-d_6) δ 173.64, 168.15, 144.06, 143.16, 142.78, 142.21, 139.87, 137.76, 131.97, 130.54 (2C), 130.19, 129.89, 129.69, 129.57, 128.41 (2C), 127.42, 127.01, 123.13, 57.60, 27.85, 15.89, 15.12. HRMS (ESI): calcd for $C_{24}H_{21}N_3O_3$ {[M+H]$^+$}, 400.1656; found, 400.1658.

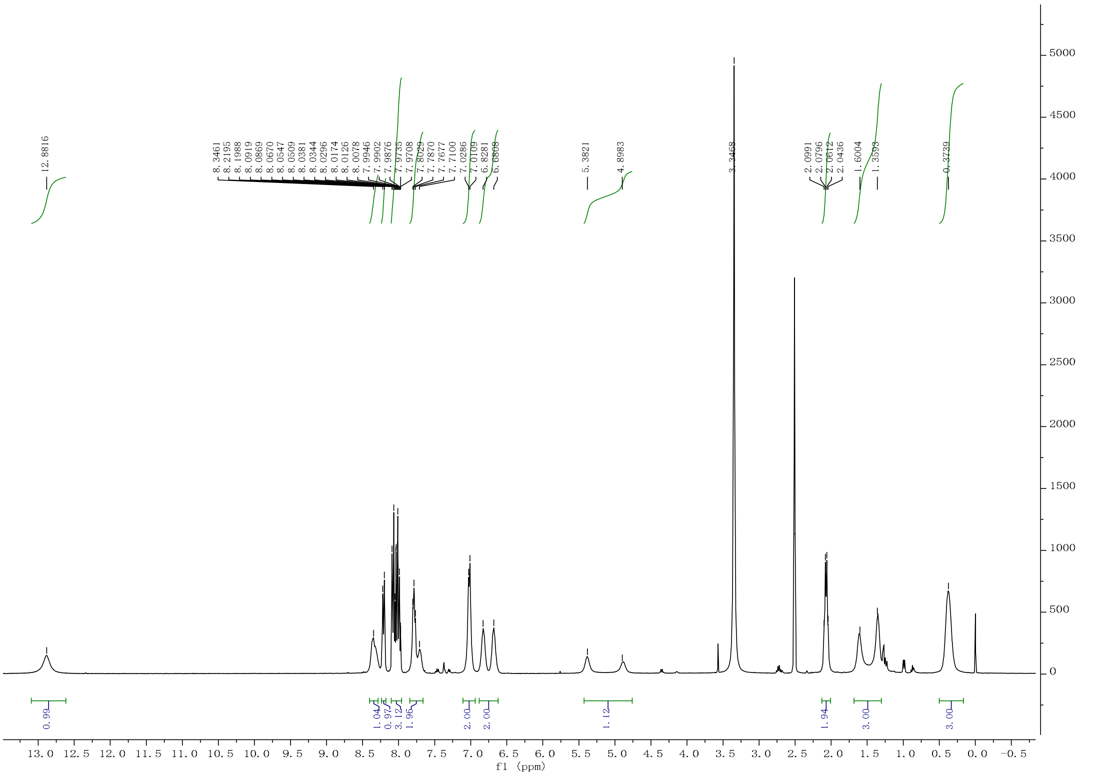

The ^{1}H NMR spectrogram of compound **F11**

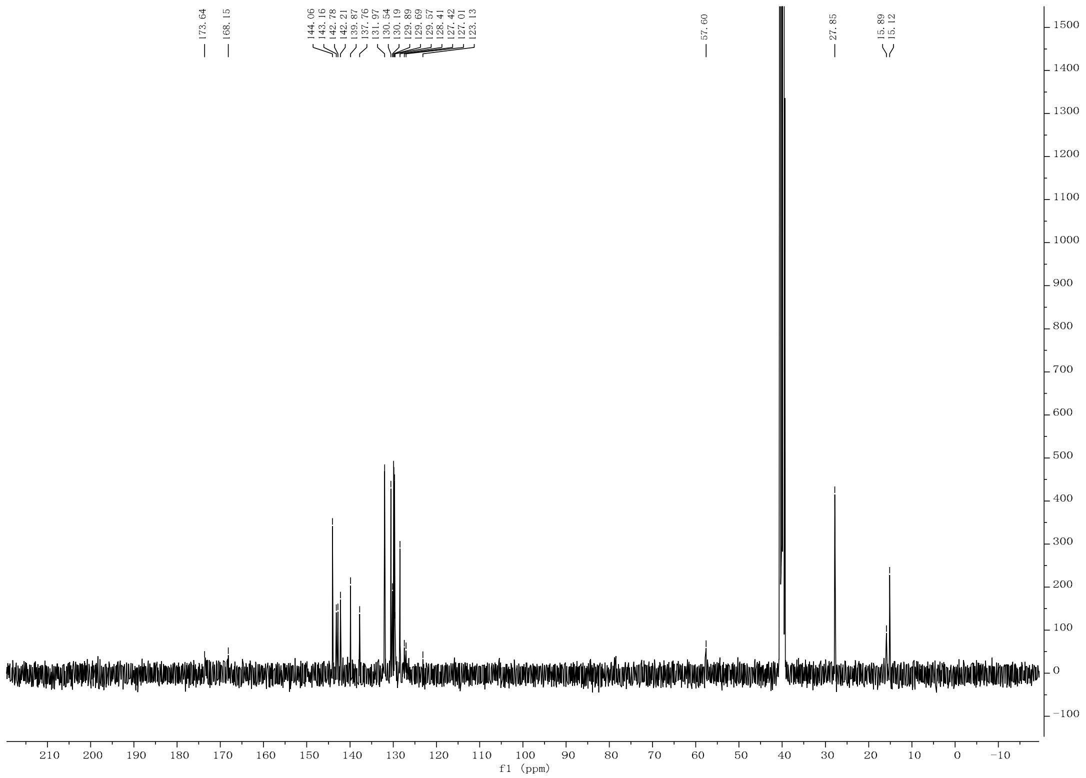

The ^{13}C NMR spectrogram of compound **F11**

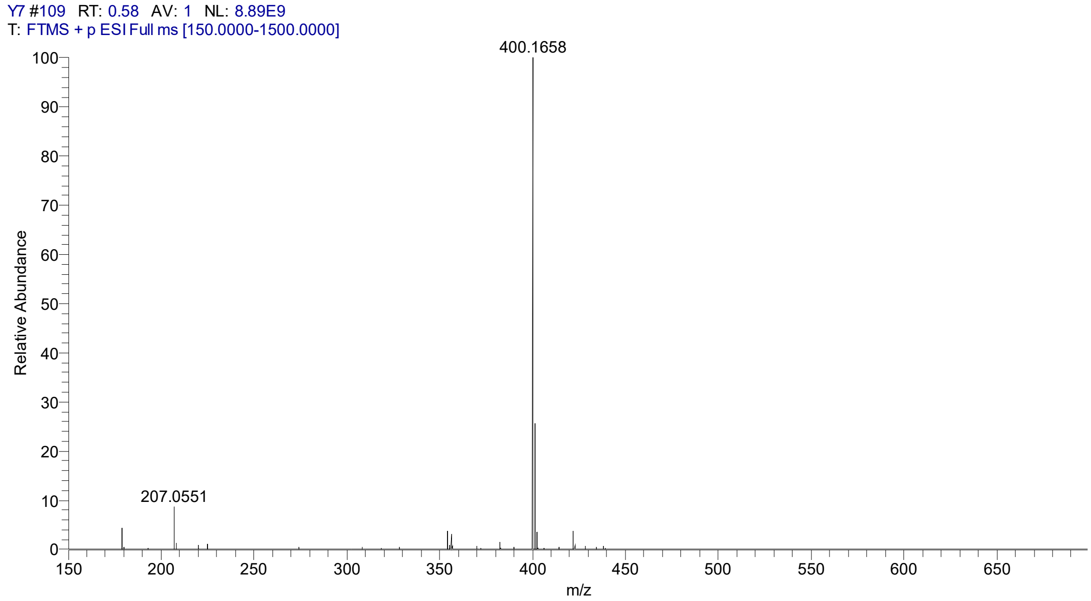

The HRMS spectrogram of compound **F11**

Compound **F12**,

N-(4-ethylphenyl)-*N*-(phenazine-1-carbonyl)alanine

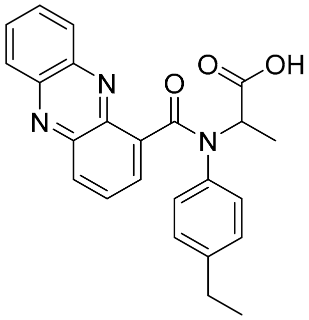

Yellow solid, yield 83.1%, m.p. 91.8-93.4°C; ^{1}H NMR (400 MHz, DMSO-*d*6) *δ* 12.86 (s, 1H), 8.33 (s, 1H), 8.21 (d, *J* = 8.1 Hz, 1H), 8.10 – 7.96 (m, 3H), 7.84 – 7.62 (m, 2H), 7.13 (d, *J* = 7.9 Hz, 2H), 6.74 (d, *J* = 7.8 Hz, 2H), 5.13 (1H, two isomers), 2.24 (q, *J* = 7.4 Hz, 2H), 1.46 (3H, two isomers), 0.85 (t, *J* = 7.5 Hz, 3H). ^{13}C NMR (101 MHz, DMSO-*d*6) *δ* 173.59, 167.54, 143.57, 143.20, 142.82, 142.27, 139.99, 137.71, 131.99, 130.58, 130.16, 129.97, 129.72, 129.50, 127.90, 121.71, 54.51, 27.71, 15.82, 15.36. HRMS (ESI): calcd for $C_{24}H_{21}N_3O_3$ {[M+H]$^+$}, 400.1656; found, 400.1662.

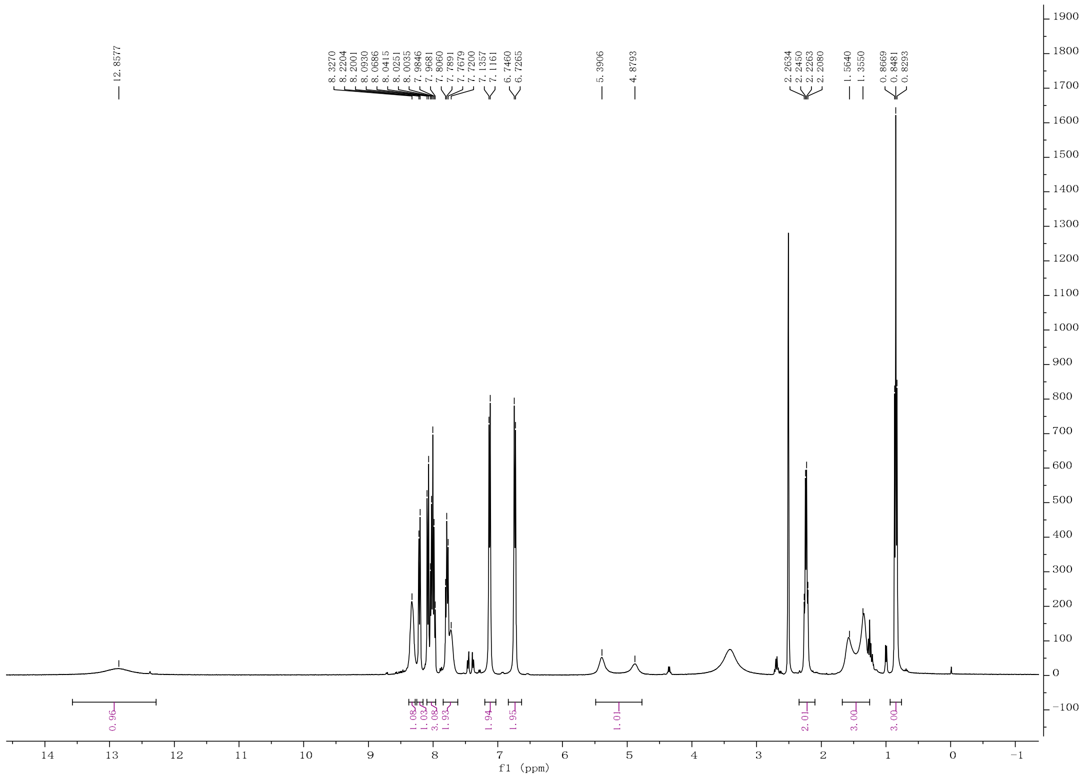

The ^{1}H NMR spectrogram of compound **F12**

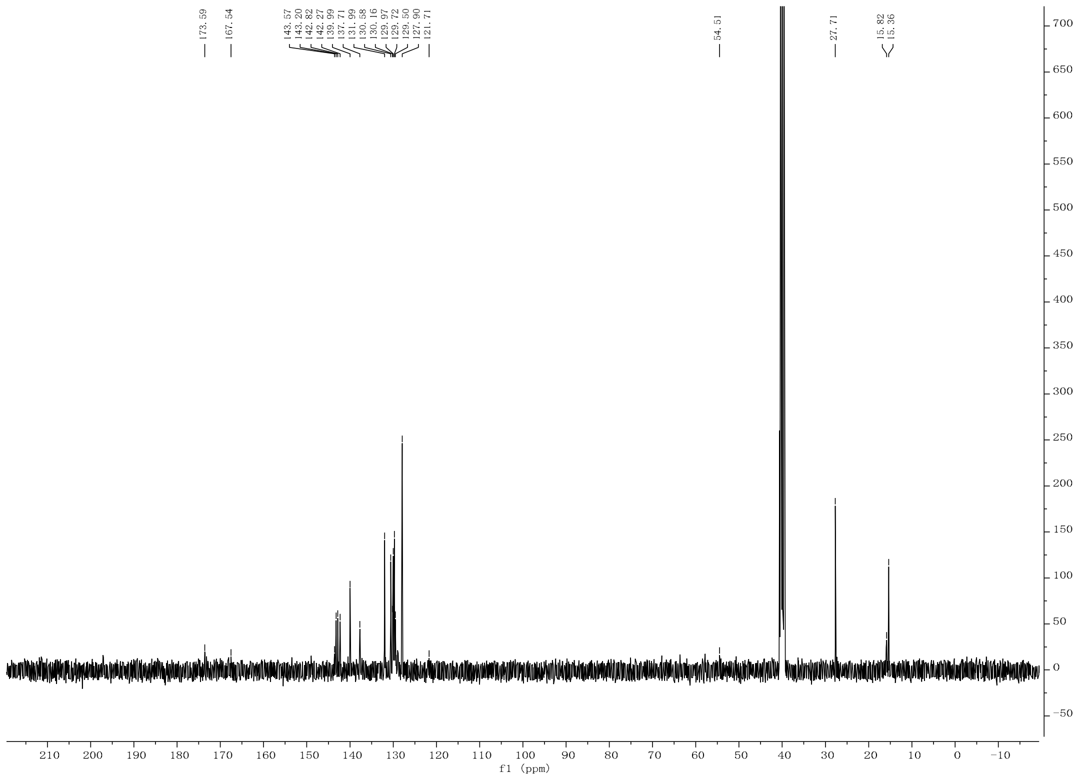

The ^{13}C NMR spectrogram of compound **F12**

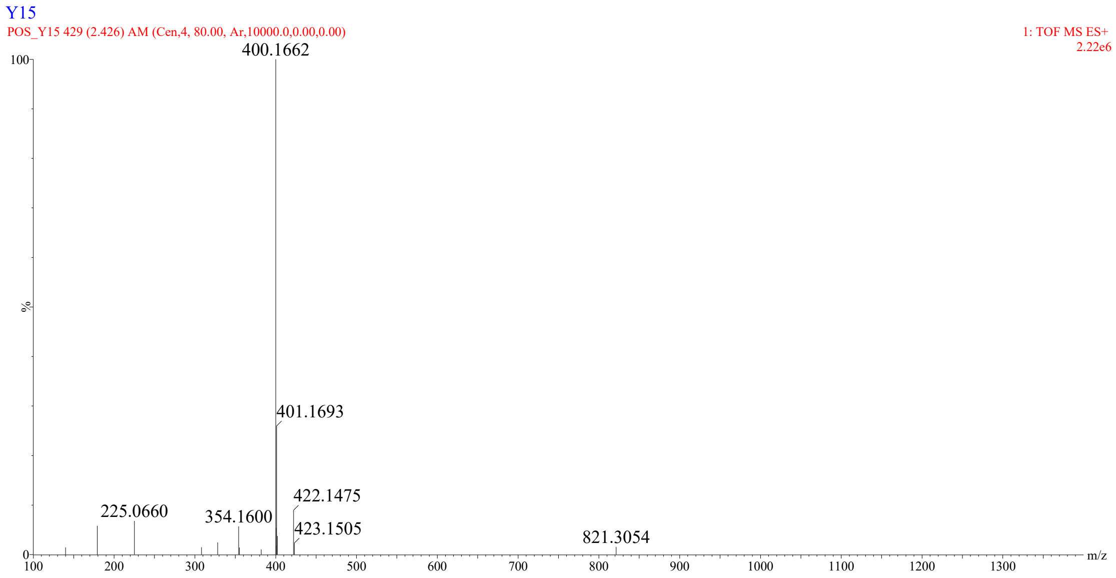

The HRMS spectrogram of compound **F12**

Compound **F13**,

N-(3-isopropylphenyl)-*N*-(phenazine-1-carbonyl)alanine

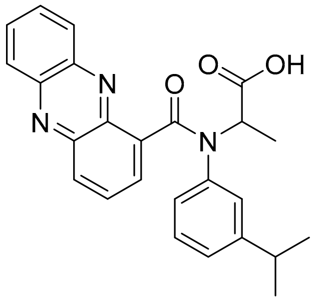

Brownness solid, yield 80.0%, m.p. 98.0-99.6℃; ^{1}H NMR (400 MHz, DMSO-d_6) δ 12.86 (s, 1H), 8.40 – 8.29 (m, 1H), 8.21 (d, J = 8.3 Hz, 1H), 8.09 – 7.95 (m, 3H), 7.82 – 7.65 (m, 2H), 7.11 – 6.81 (m, 3H), 6.70 (s, 1H), 5.17 (1H, two isomers), 2.32 (t, J = 6.6 Hz, 1H), 1.48 (3H, two isomers), 0.70 – 0.18 (m, 6H). ^{13}C NMR (101 MHz, DMSO-d_6) δ 173.59, 168.51, 148.57, 143.20, 142.84, 142.23, 139.92, 137.81, 131.96 (2C), 130.53 (4C), 130.12 (2C), 129.91, 129.67, 128.48, 127.01, 126.35, 54.28, 32.96, 23.36, 15.93. HRMS (ESI): calcd for $C_{25}H_{23}N_3O_3$ {[M+H]$^+$}, 414.1813; found, 414.1817.

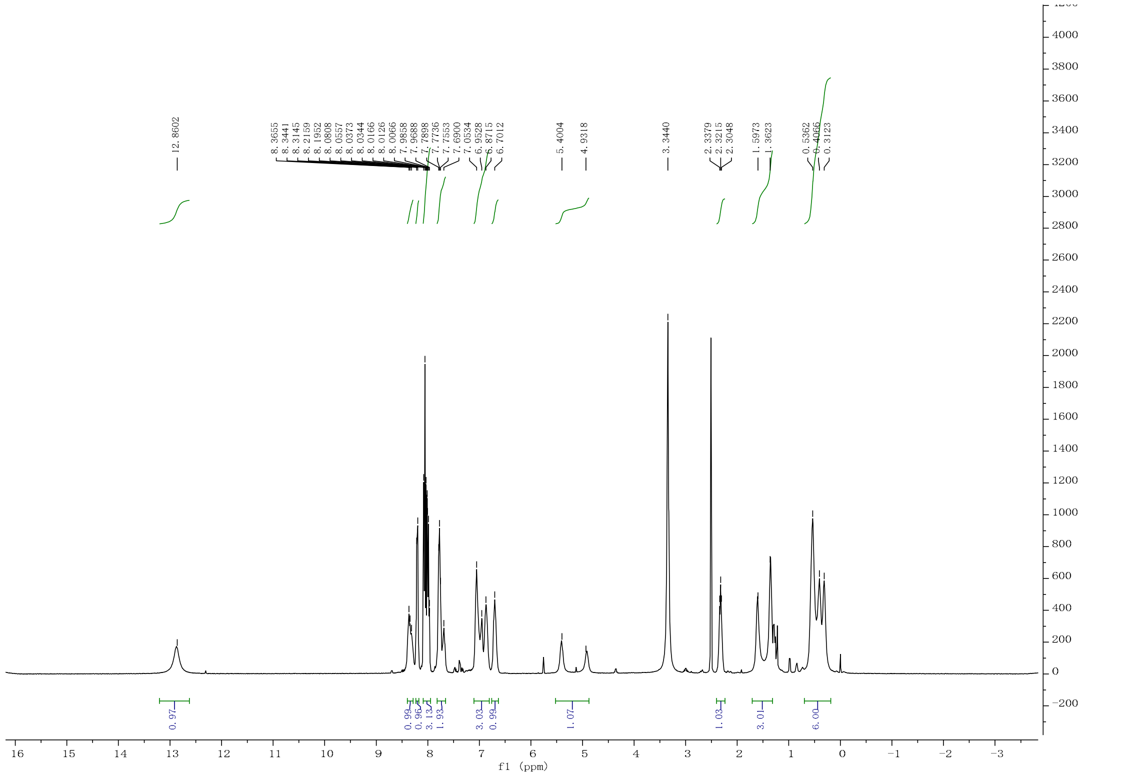

The ^{1}H NMR spectrogram of compound **F13**

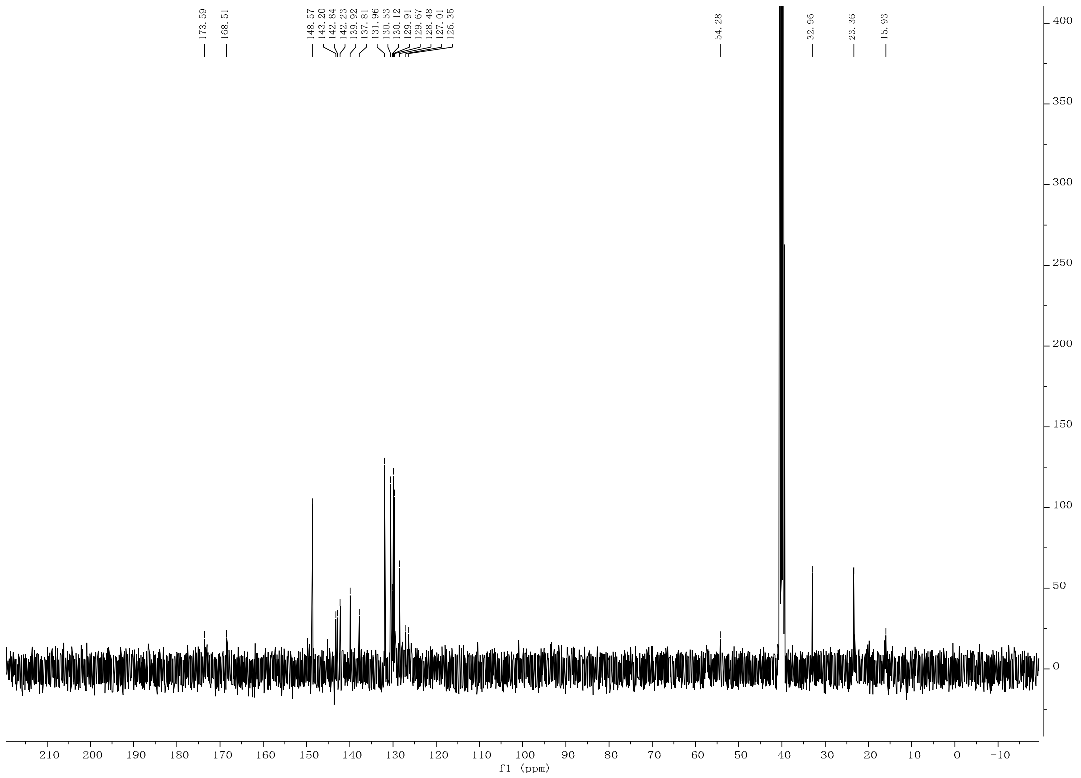

The ^{13}C NMR spectrogram of compound **F13**

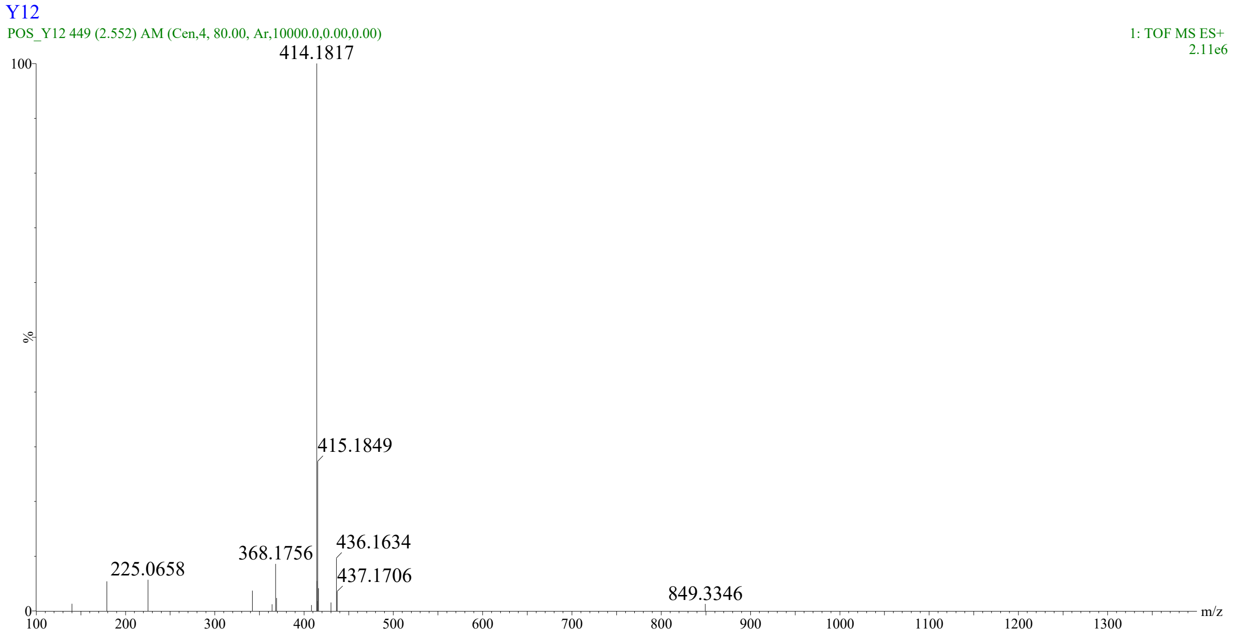

The HRMS spectrogram of compound **F13**

Compound **F14**,

N-(4-isopropylphenyl)-*N*-(phenazine-1-carbonyl)alanine

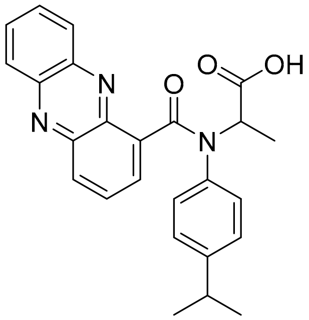

Yellow solid, yield 81.7%, m.p. 71.9-73.7°C; ^{1}H NMR (400 MHz, Chloroform-*d*) δ 8.45 (d, J = 8.1 Hz, 1H), 8.24 (d, J = 7.9 Hz, 1H), 8.16 (d, J = 8.7 Hz, 1H), 8.02 – 7.87 (m, 3H), 7.76 (s, 1H), 7.01 (d, J = 6.8 Hz, 2H), 6.75 (d, J = 8.4 Hz, 2H), 5.91 (s, 1H), 2.57 (sep, J = 6.8 Hz, 1H), 1.52 (s, 3H), 0.95 (d, J = 6.9 Hz, 6H). ^{13}C NMR (101 MHz, DMSO-d_6) δ 173.55, 168.26, 143.17, 142.79, 142.27 (2C), 140.00, 137.68, 131.93, 130.52, 130.16, 129.97, 129.692 (2C), 129.55 (2C), 127.38 (2C), 126.35 (2C), 54.60, 32.97, 24.33, 23.80, 15.89. HRMS (ESI): calcd for $C_{25}H_{23}N_3O_3$ {[M+H]$^+$}, 414.1813; found, 414.1818.

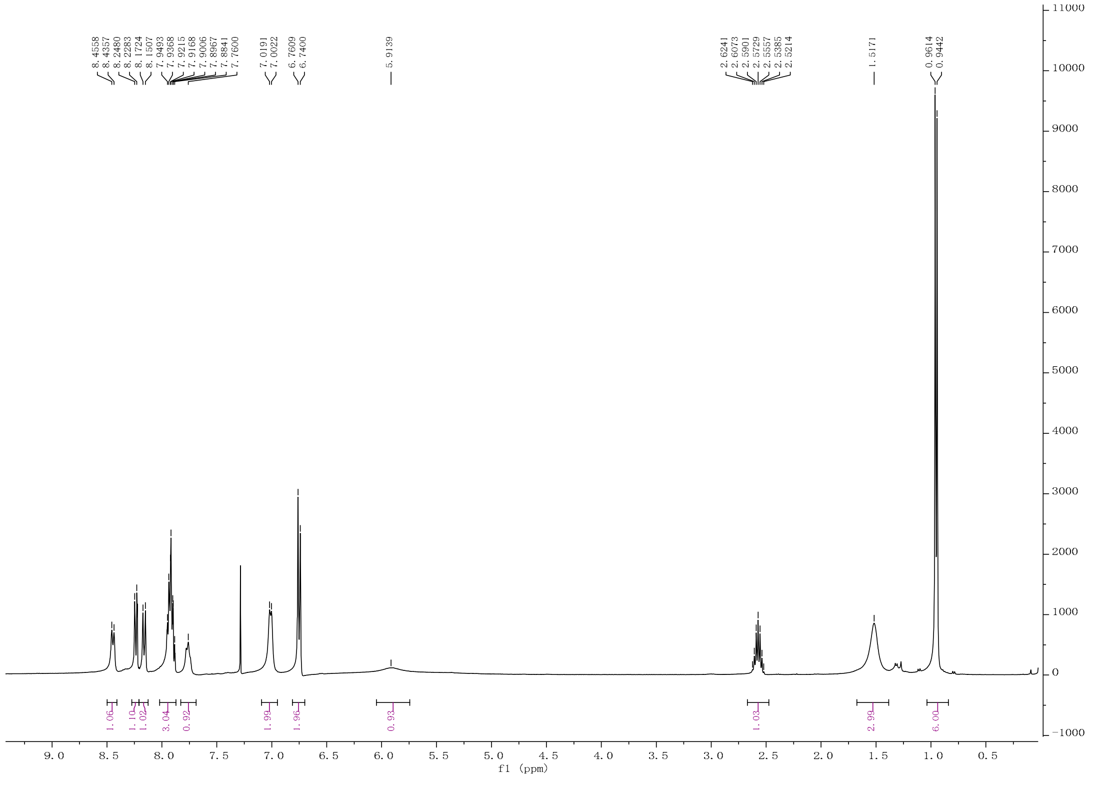

The ^{1}H NMR spectrogram of compound **F14**

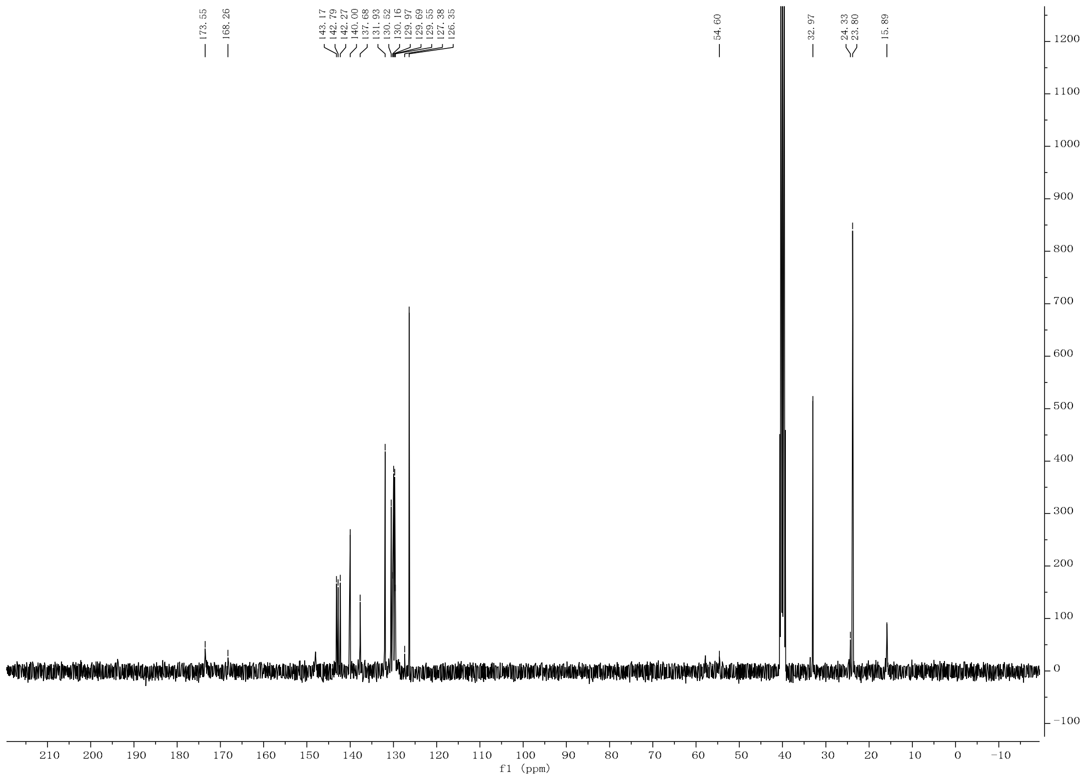

The ^{13}C NMR spectrogram of compound **F14**

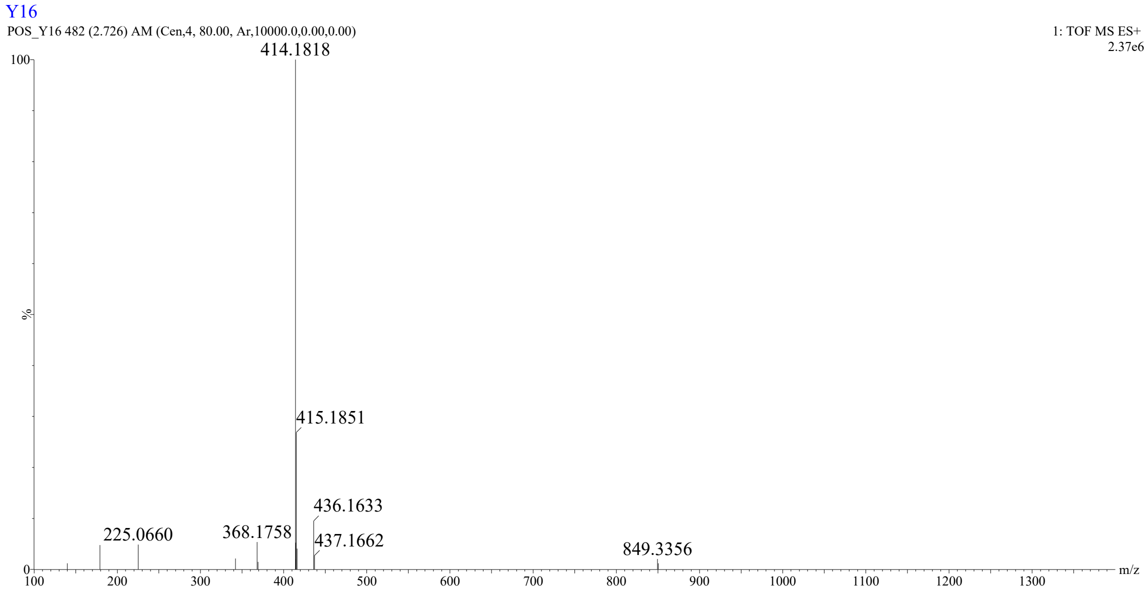

The HRMS spectrogram of compound **F14**

Compound **F15**,

N-(2,6-dimethylphenyl)-*N*-(phenazine-1-carbonyl)alanine

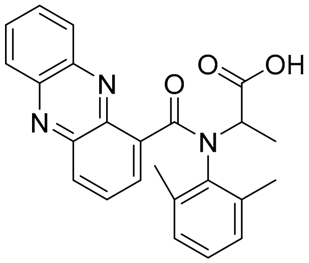

Yellow solid, yield 79.3%, m.p. 103.4-104.8°C; ^{1}H NMR (400 MHz, DMSO-d_6) δ 12.68 (s, 1H), 8.26 – 8.18 (m, 2H), 8.09 (td, *J* = 5.5, 4.6, 2.6 Hz, 1H), 8.03 – 7.94 (m, 2H), 7.81 (d, *J* = 6.3 Hz, 2H), 6.81 – 6.62 (m, 3H), 4.70 (1H, two isomers), 2.47 (d, *J* = 11.5 Hz, 6H), 1.12 (3H, two isomers). ^{13}C NMR (101 MHz, DMSO-d_6) *δ* 173.66, 168.93, 143.08, 142.36, 142.31, 140.19, 138.40, 137.00 (2C), 132.06, 131.97, 130.47, 130.27 (2C), 129.80, 129.50 (2C), 128.73, 128.38, 128.29, 50.65, 19.44 (2C), 15.55. HRMS (ESI): calcd for $C_{24}H_{21}N_3O_3$ {[M+H]$^+$}, 400.1656; found, 400.1660.

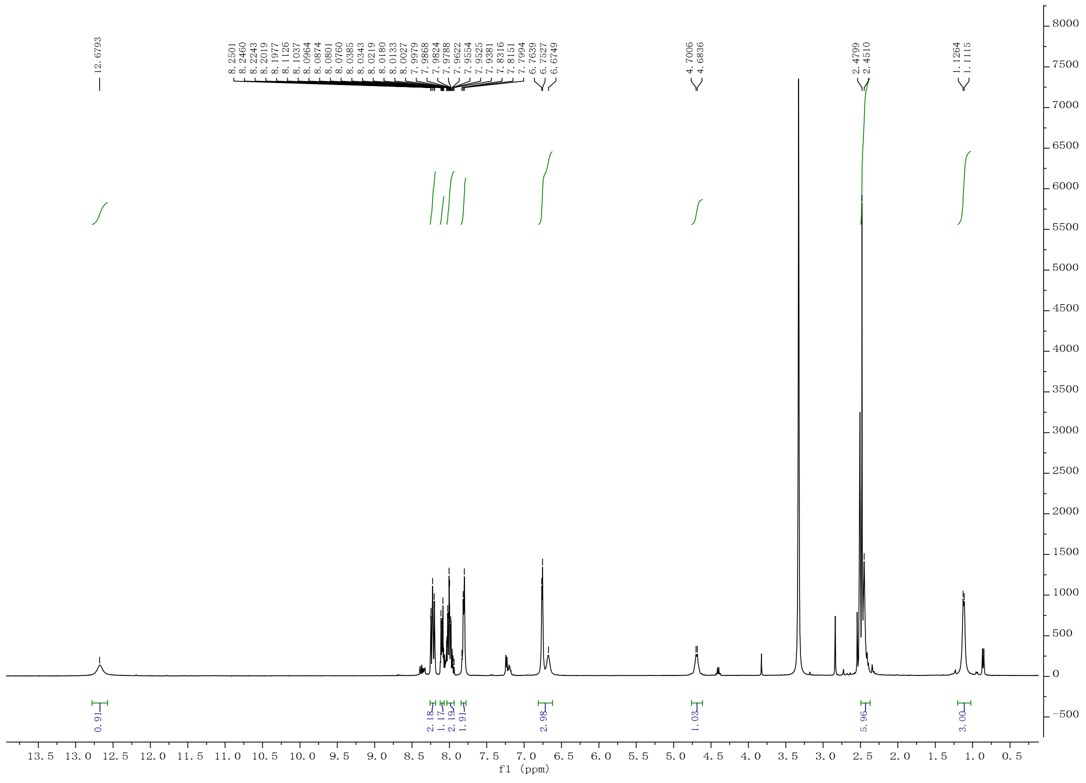

The ^{1}H NMR spectrogram of compound **F15**

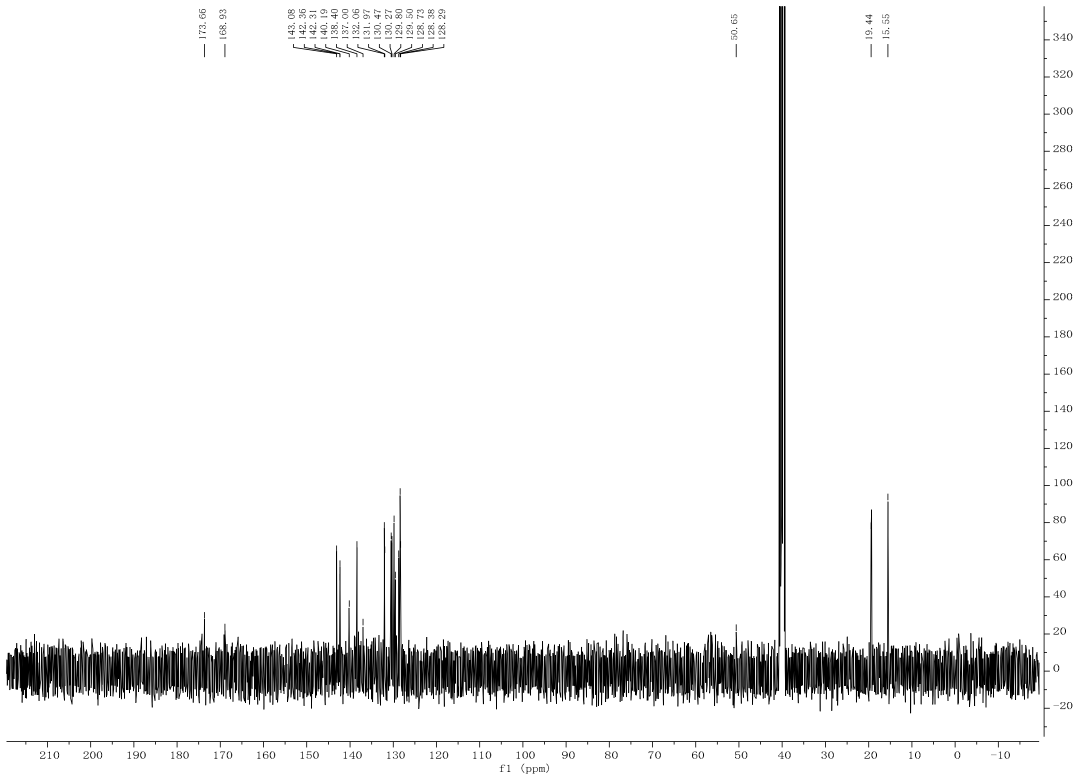

The ^{13}C NMR spectrogram of compound **F15**

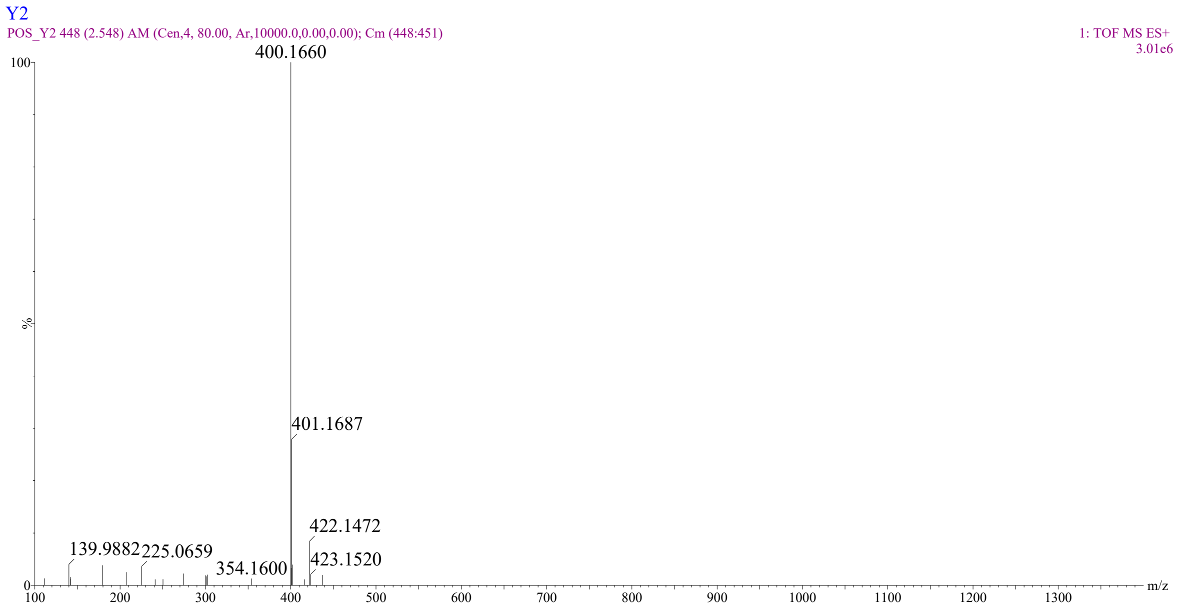

The HRMS spectrogram of compound **F15**

Compound **F16**,

N-(3-nitrophenyl)-*N*-(phenazine-1-carbonyl)alanine

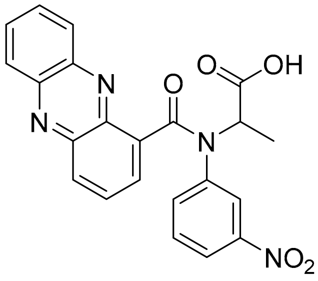

Yellow solid, yield 77.9%, m.p. 99.3-100.8°C; ^{1}H NMR (400 MHz, DMSO-d_6) δ 13.10 (s, 1H), 8.41 – 8.28 (m, 1H), 8.25 – 8.09 (m, 3H), 8.09 – 7.90 (m, 3H), 7.83 (s, 1H), 7.78 – 7.50 (m, 2H), 7.20 (t, J = 7.4 Hz, 1H), 5.25 (1H, two isomers), 1.51 (3H, two isomers). ^{13}C NMR (101 MHz, DMSO-d_6) δ 173.08, 167.95, 147.23, 143.23, 142.79, 142.14, 139.35, 136.77, 132.16, 130.96 (2C), 130.61 (2C), 130.31 (2C), 129.86 (2C), 129.67, 124.96, 123.00, 55.20, 15.91. HRMS (ESI): calcd for $C_{22}H_{16}N_4O_5$ {[M+H]$^+$}, 417.1193; found, 417.1195.

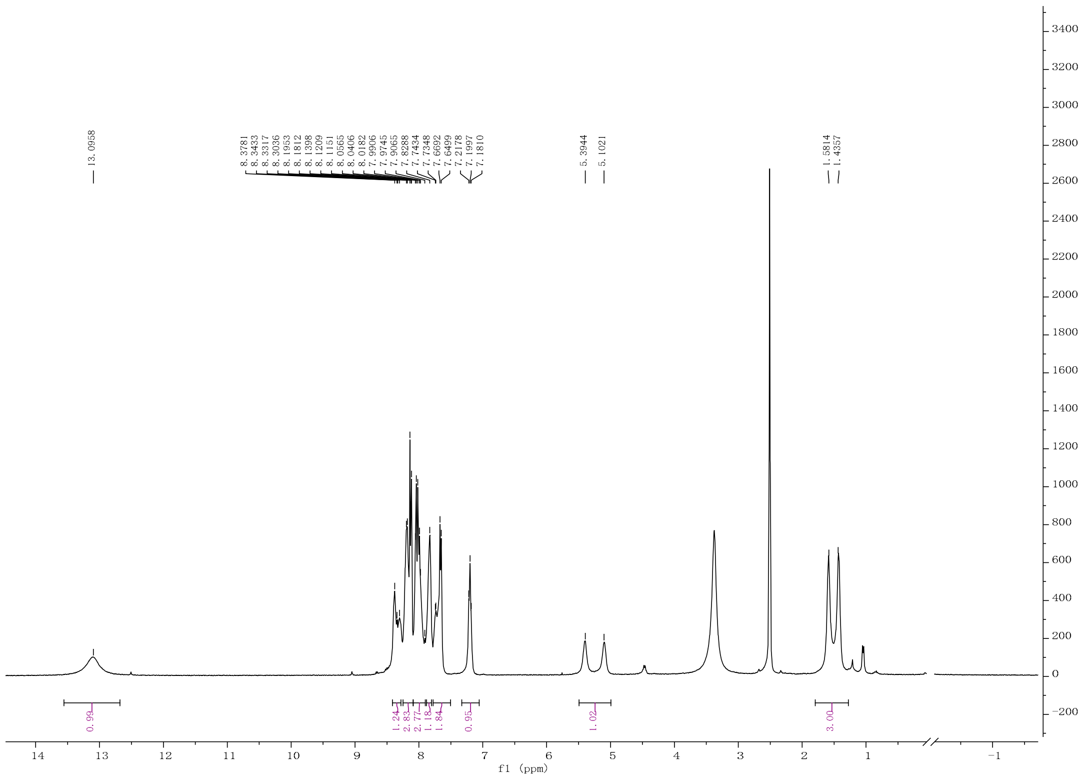

The ^{1}H NMR spectrogram of compound **F16**

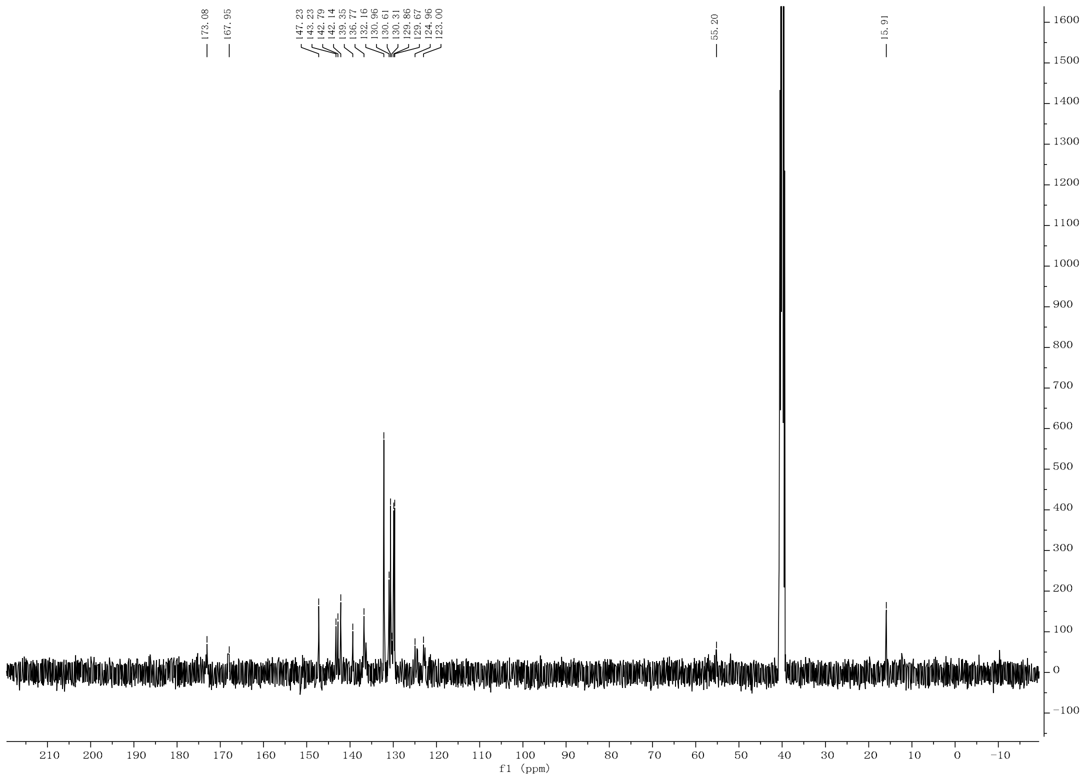

The ^{13}C NMR spectrogram of compound **F16**

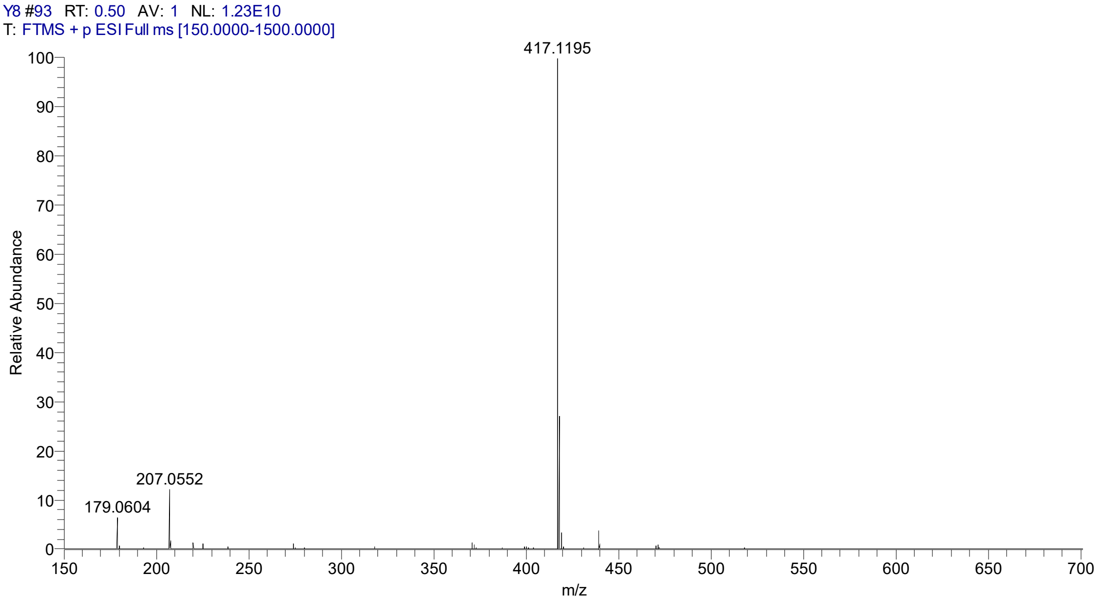

The HRMS spectrogram of compound **F16**

Compound **E1**, methyl *N*-(phenazine-1-carbonyl)-*N*-phenylalaninate

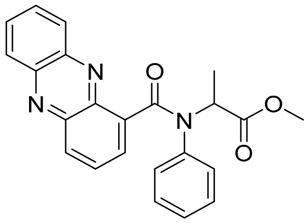

Yellow solid, yield 83.2%, m.p. 150.4-152.1°C; ^{1}H NMR (400 MHz, $CDCl_3$) δ 8.37 (d, J = 9.5 Hz, 1H), 8.21 (d, J = 9.4 Hz, 1H), 8.07 (d, J = 8.6 Hz, 1H), 7.88 (p, J = 6.8 Hz, 2H), 7.73 – 7.60 (m, 2H), 7.29 – 7.22 (m, 2H), 6.91 (s, 3H), 5.60 (s, 1H), 3.94 (s, 3H), 1.60 (d, J = 25.7 Hz, 3H). ^{13}C NMR (101 MHz, $CDCl_3$) δ 169.00, 162.78, 143.37, 143.26, 142.47, 140.41, 137.20, 130.82, 130.52 (2C), 130.36 (2C), 130.19, 129.57 (2C), 129.30, 129.00, 128.33, 127.79 (2C), 52.42, 33.57, 15.58. HRMS (ESI): calcd for $C_{23}H_{19}N_3O_3$ {[M+H]$^+$}, 386.1499; found, 386.1499.

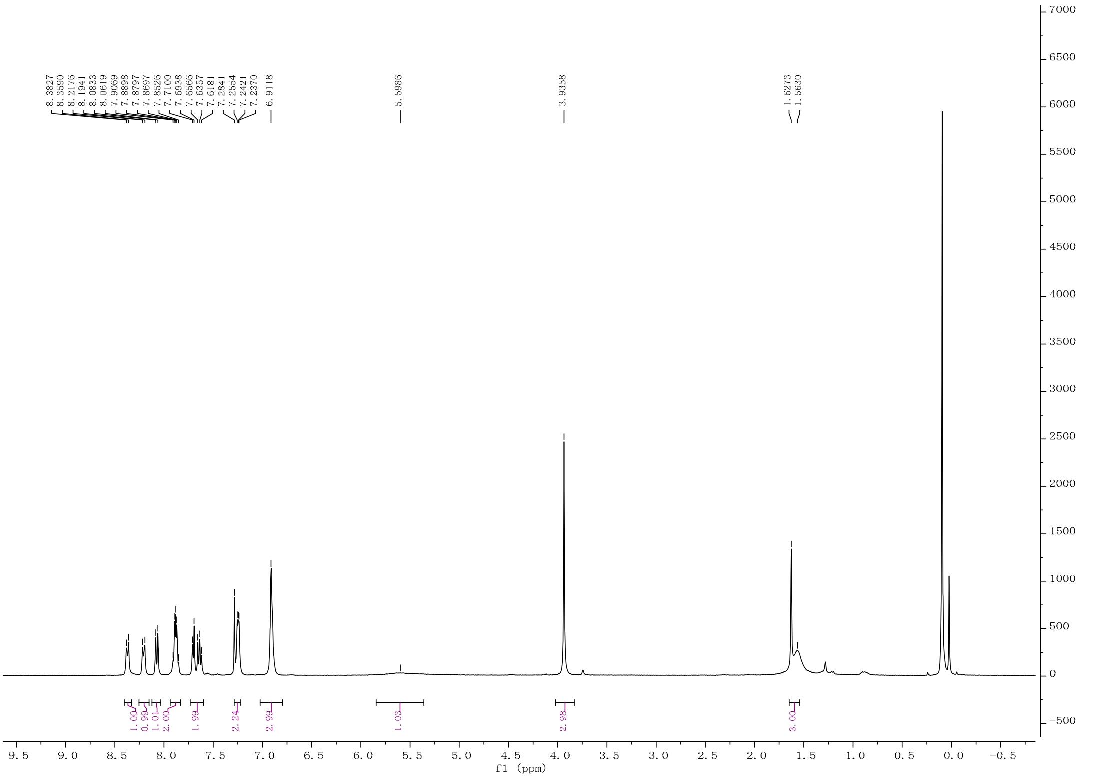

The ^{1}H NMR spectrogram of compound **E1**

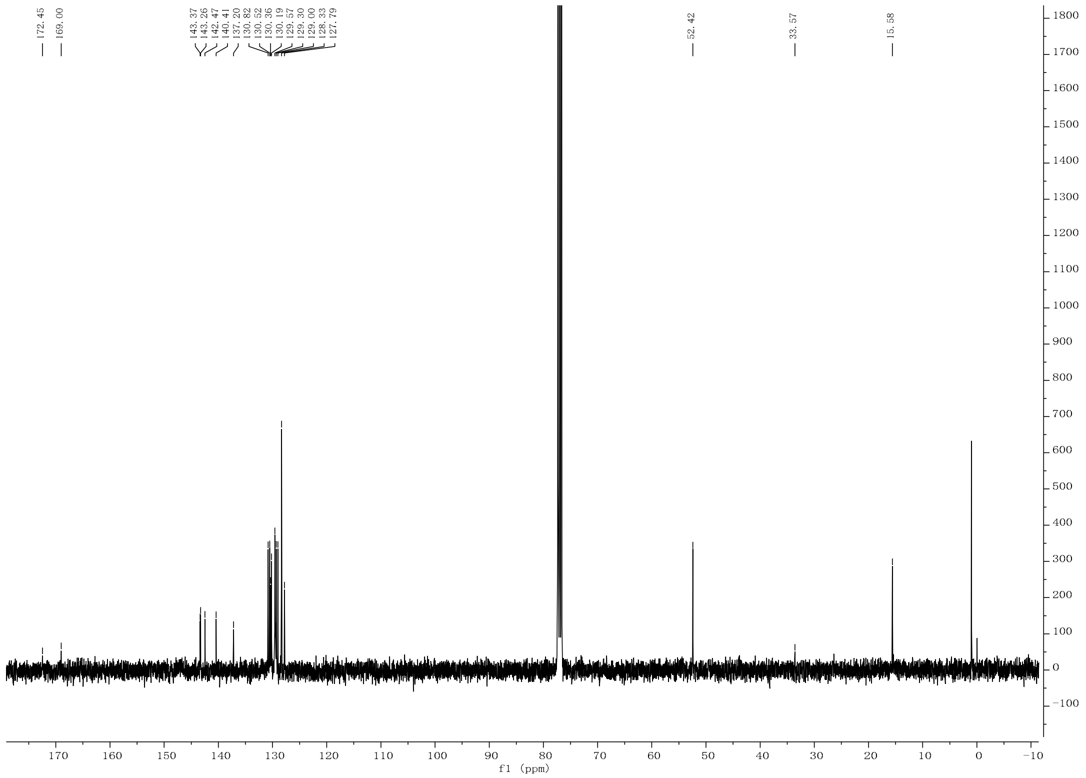

The ^{13}C NMR spectrogram of compound **E1**

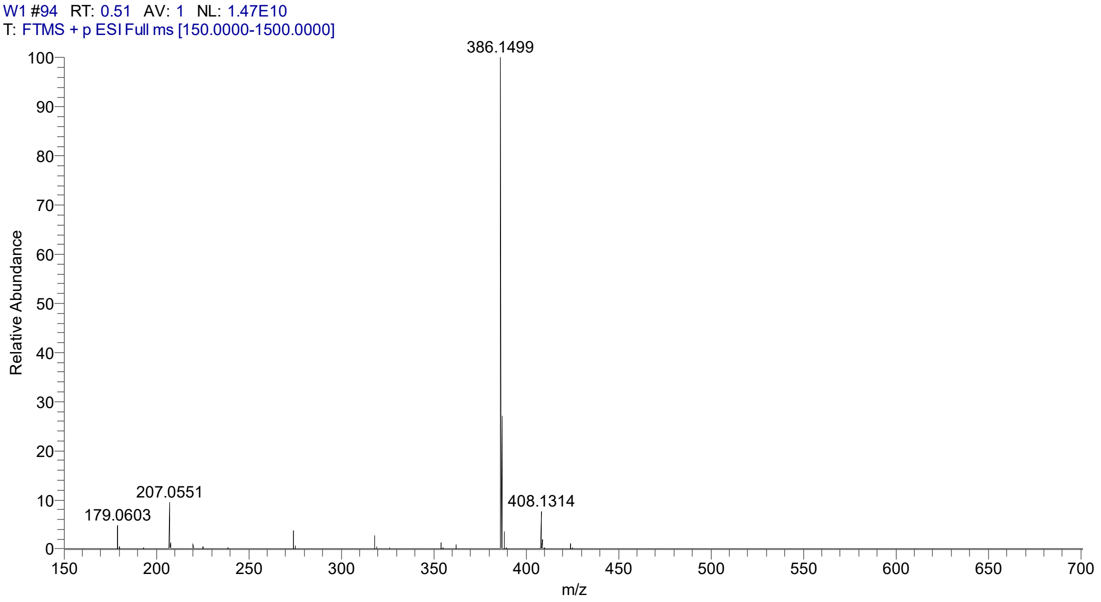

The HRMS spectrogram of compound **E1**

Compound **E2**, methyl *N*-(2-fluorophenyl)-*N*-(phenazine-1-carbonyl)alaninate

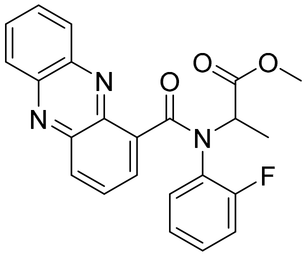

Yellow solid, yield 82.1%, m.p. 142.1-143.9°C; ^{1}H NMR (400 MHz, DMSO-d_6) δ 8.35 – 8.25 (m, 1H), 8.16 (dd, J = 24.2, 7.9 Hz, 2H), 8.02 (d, J = 27.1 Hz, 2H), 7.86 (s, 2H), 7.38 (s, 1H), 6.95 (s, 2H), 6.59 (s, 1H), 5.22 (1H, two isomers), 3.85 (d, J = 25.6 Hz, 3H), 1.62 (3H, two isomers). ^{13}C NMR (101 MHz, DMSO-d_6) δ 171.72, 168.20, 165.14, 162.90(dd, C=C-F= J =226.24 Hz), 143.19, 142.74, 142.01, 139.59, 136.57, 132.08, 131.46 (2C), 131.05 (2C), 1+30.92, 130.53 (2C), 129.86, 129.71, 124.31, 116.07, 55.23, 52.76, 15.04. HRMS (ESI): calcd for $C_{23}H_{18}FN_3O_3$ {[M+H]$^+$}, 404.1405; found, 404.1406.

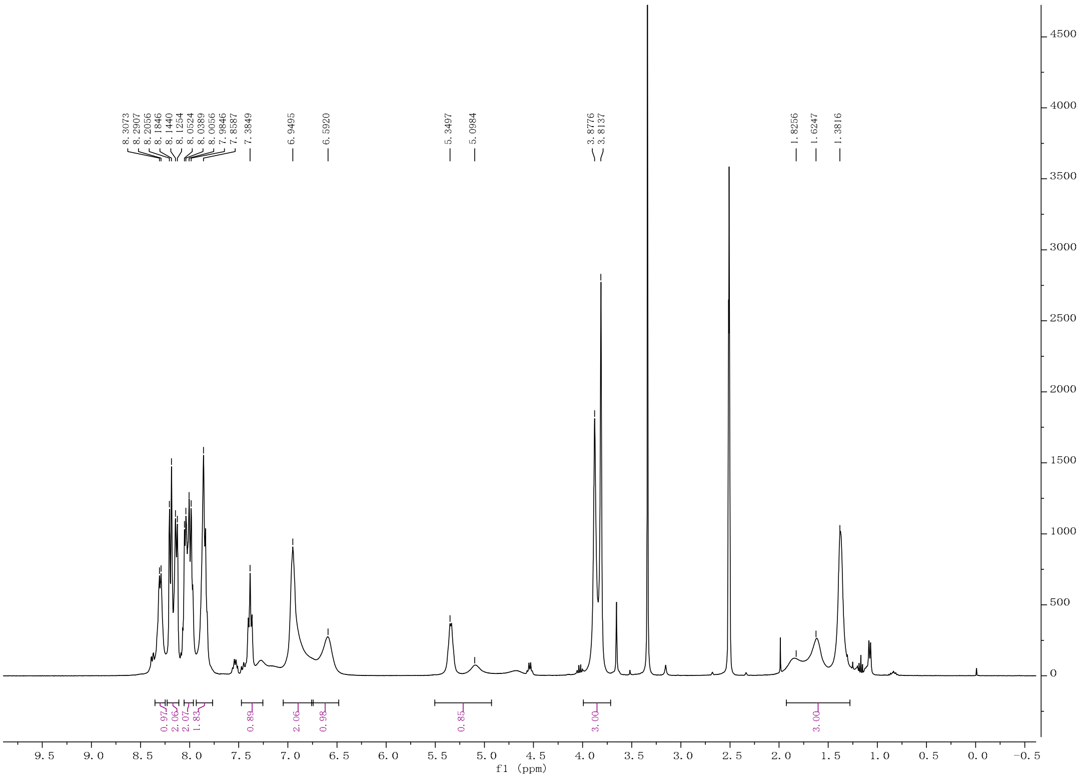

The ^{1}H NMR spectrogram of compound **E2**

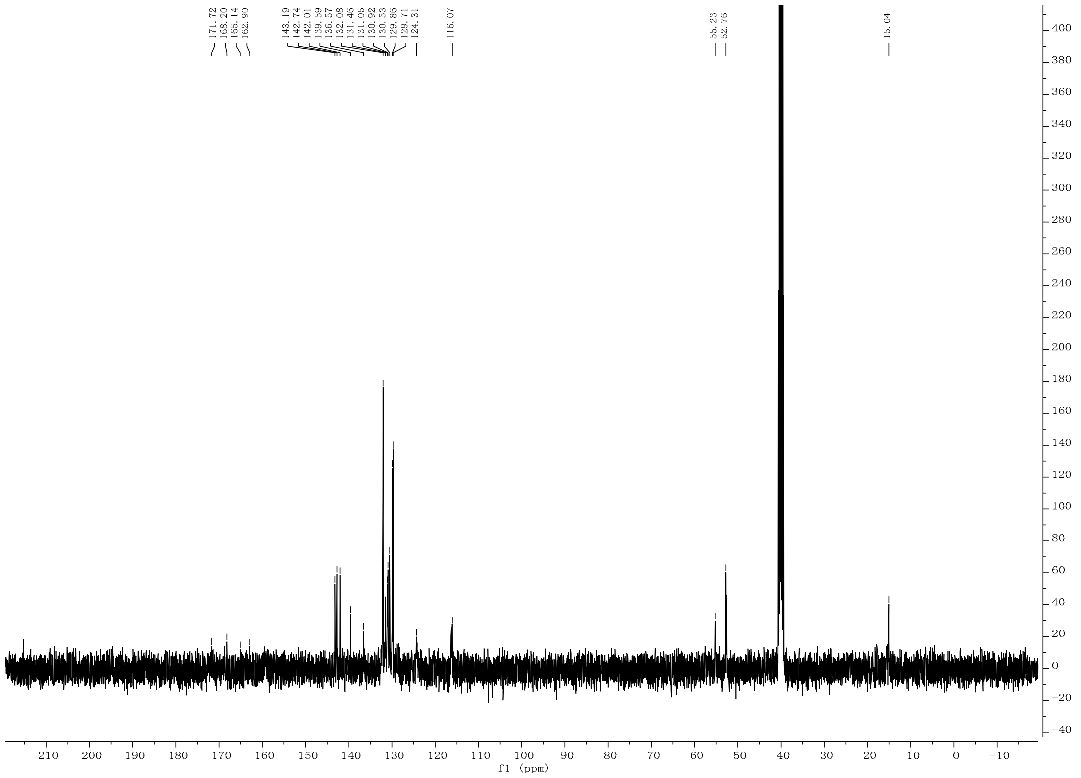

The ^{13}C NMR spectrogram of compound **E2**

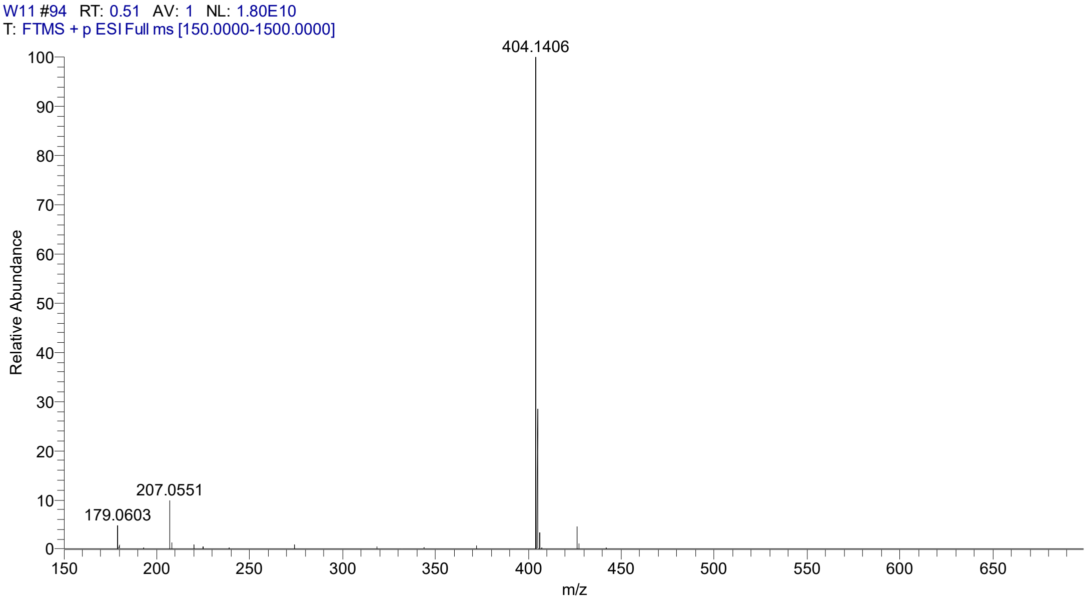

The HRMS spectrogram of compound **E2**

Compound **E3**,
methyl *N*-(3-fluorophenyl)-*N*-(phenazine-1-carbonyl)alaninate

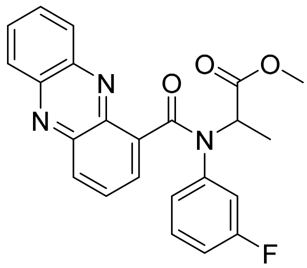

Yellow solid, yield 80.2%, m.p. 145.3-147.2℃; ^{1}H NMR (400 MHz, DMSO-d_6) δ 8.33 (s, 1H), 8.22 (d, J = 8.4 Hz, 1H), 8.14 (d, J = 10.0 Hz, 1H), 8.07 – 7.97 (m, 2H), 7.86 (q, J = 11.7, 7.0 Hz, 2H), 7.12 (d, J = 9.4 Hz, 1H), 7.02 (d, J = 8.1 Hz, 1H), 6.94 (d, J = 7.1 Hz, 1H), 6.77 (s, 1H), 5.21 (1H, two isomers), 3.85 (s, 3H), 1.53 (3H, two isomers).^{13}C NMR (101 MHz, DMSO-d_6) δ 172.11, 168.01, 162.67, 160.23, (dd, C=C-*f*=*J*= 246.44 Hz), 143.22, 142.77, 142.18, 139.65, 136.88, 132.16, 130.72, 130.57, 130.20, 130.12 (2C), 129.91, 129.74 (2C), 126.25, 116.82, 115.13, 52.76, 15.70. HRMS (ESI): calcd for $C_{23}H_{18}FN_3O_3$ {[M+H]$^+$}, 404.1405; found, 404.1408.

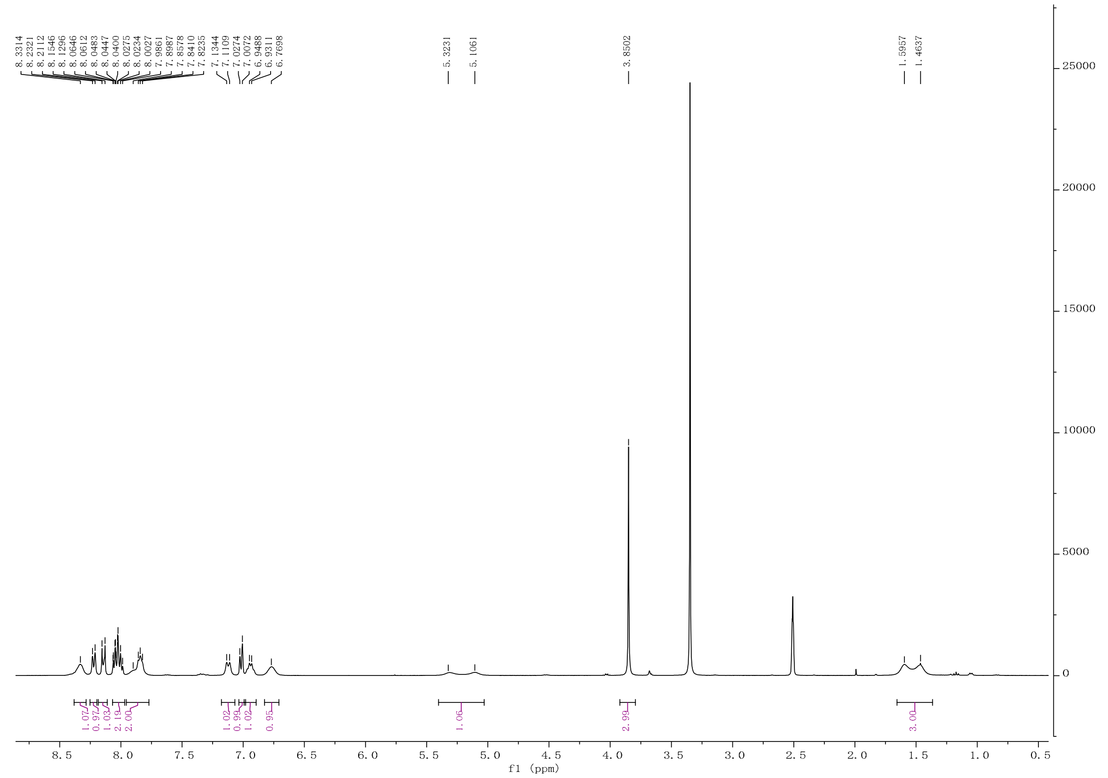

The ^{1}H NMR spectrogram of compound **E3**

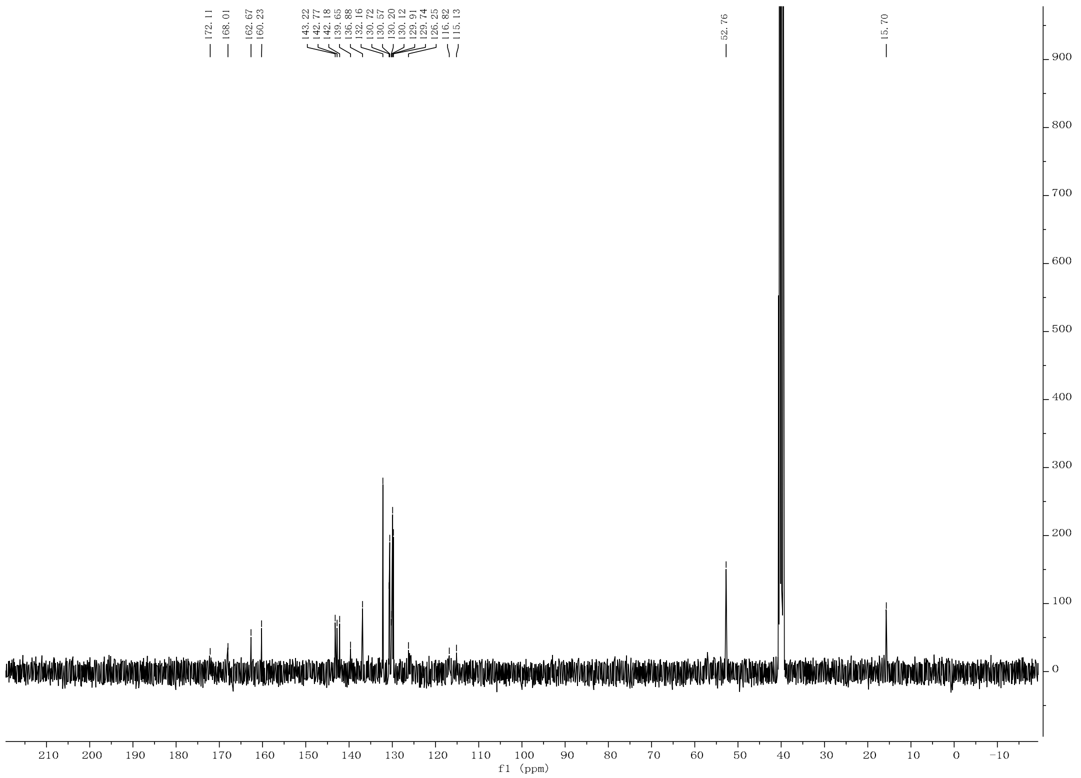

The ^{13}C NMR spectrogram of compound **E3**

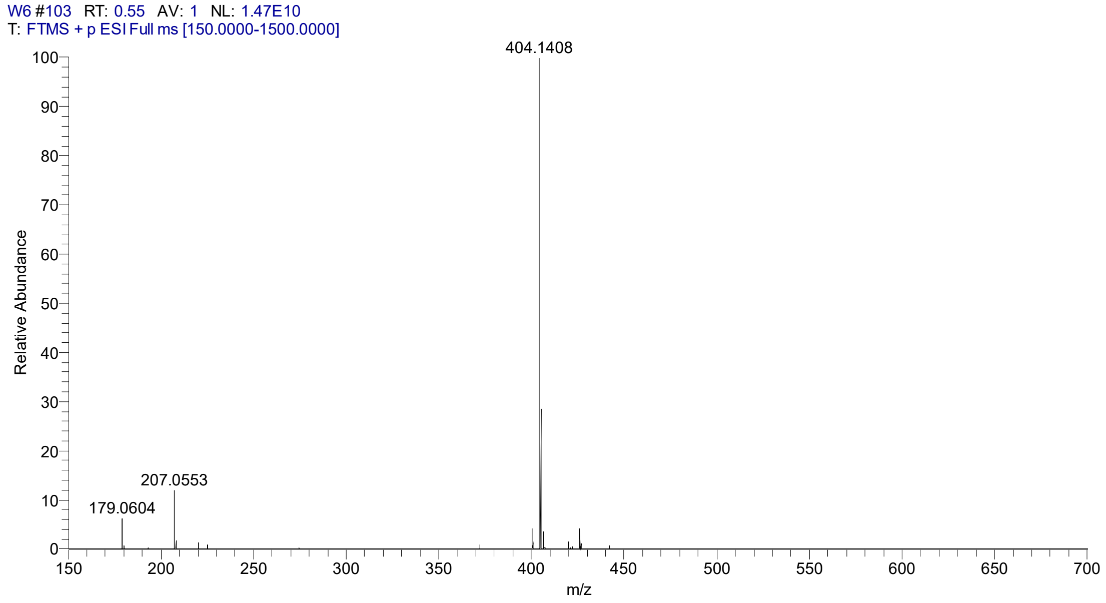

The HRMS spectrogram of compound **E3**

Compound **E4**,
methyl *N*-(4-fluorophenyl)-*N*-(phenazine-1-carbonyl)alaninate

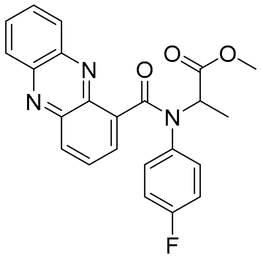

Yellow solid, yield 79.6%, m.p. 160.3-162.1°C; ^{1}H NMR (400 MHz, DMSO-*d*6) δ 8.33 (s, 1H), 8.27 – 8.19 (m, 1H), 8.16 – 8.10 (m, 1H), 8.02 (dddd, *J* = 14.9, 8.1, 6.6, 1.6 Hz, 2H), 7.91 – 7.73 (m, 2H), 7.35 – 7.17 (m, 2H), 6.77 (t, *J* = 8.3 Hz, 2H), 5.24 (1H, two isomers), 3.84 (s, 3H), 1.49 (3H, two isomers).^{13}C NMR (101 MHz, DMSO-*d*6) δ 169.01, 163.94, 159.75, 157.16 (dd, C=C-*f*=*J*= 261.59 Hz), 143.11, 142.35, 142.26, 140.13, 138.27, 132.12, 132.01, 130.59, 130.25 (2C), 129.80, 129.52, 128.79, 128.51, 128.40, 98.36 (2C), 52.45, 19.29, 15.49. HRMS (ESI): calcd for $C_{23}H_{18}FN_3O_3$ {[M+H]$^+$}, 404.1405; found, 404.1407.

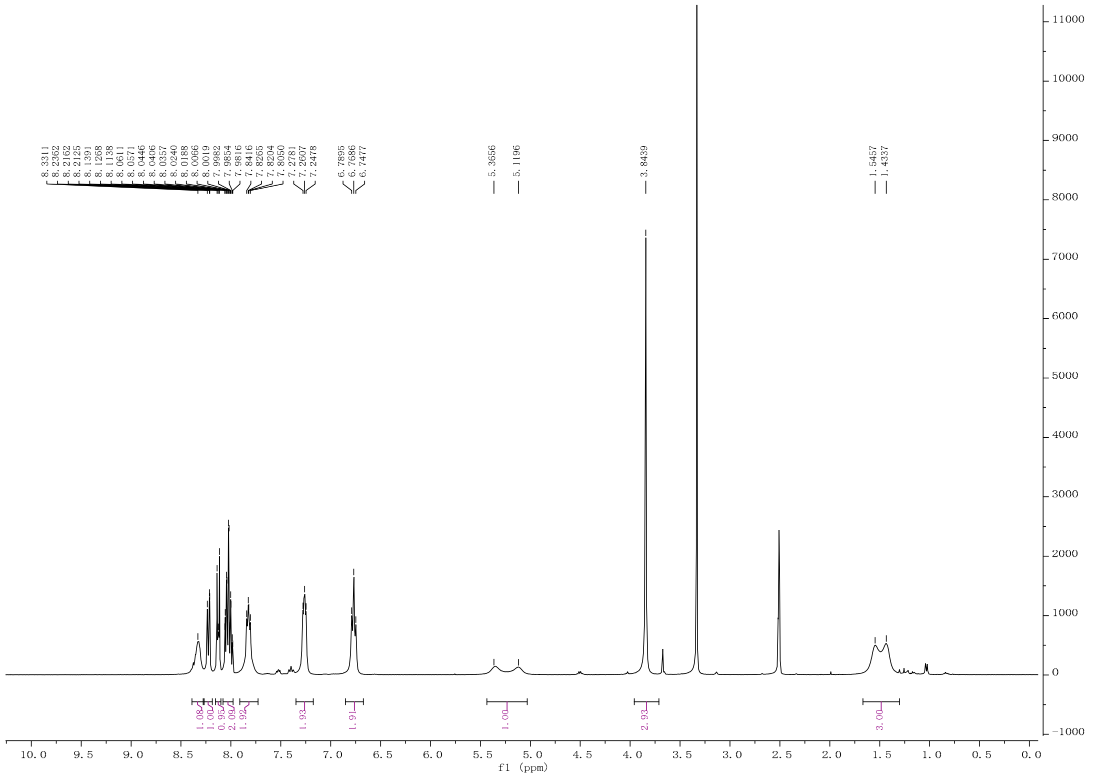

The ^{1}H NMR spectrogram of compound **E4**

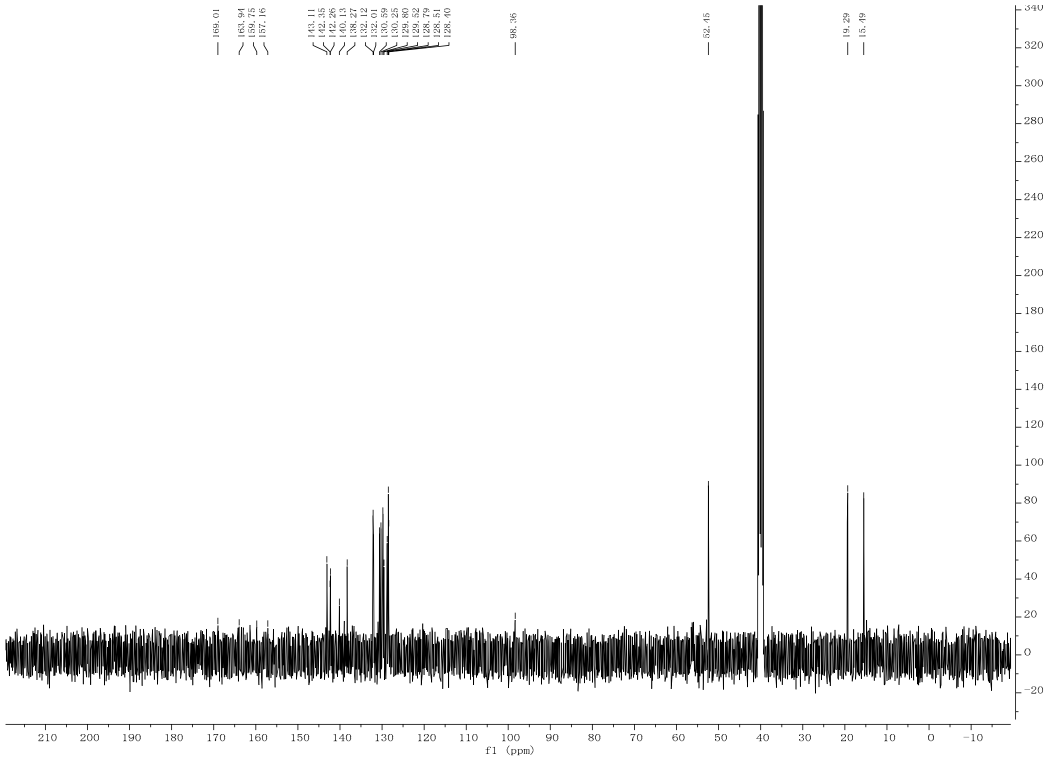

The ^{13}C NMR spectrogram of compound **E4**

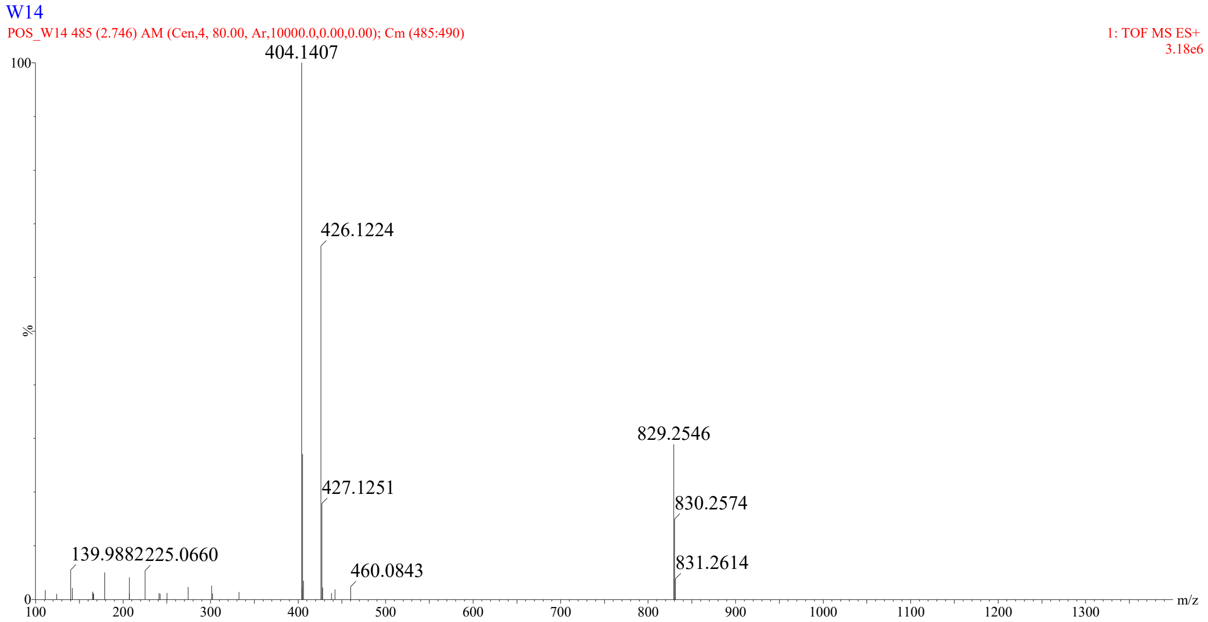

The HRMS spectrogram of compound **E4**

Compound **E5**,

methyl *N*-(2-chlorophenyl)-*N*-(phenazine-1-carbonyl)alaninate

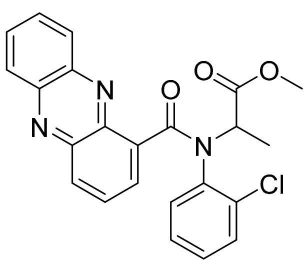

Yellow solid, yield 80.1%, m.p. 144.7-146.6°C; ^{1}H NMR (400 MHz, DMSO-d_6) δ 8.32 (s, 1H), 8.23 (d, J = 8.4 Hz, 1H), 8.14 (d, J = 10.1 Hz, 1H), 8.02 (dddd, J = 14.3, 7.9, 6.6, 1.5 Hz, 2H), 7.93 – 7.75 (m, 2H), 7.24 (d, J = 8.1 Hz, 2H), 7.01 (d, J = 8.1 Hz, 2H), 5.22 (1H, two isomers), 3.84 (s, 3H), 1.50 (3H, two isomers).^{13}C NMR (101 MHz, DMSO-d_6) δ 172.19, 168.24, 143.28, 142.83, 142.21, 139.70, 136.95, 132.13, 131.31, 130.65 (2C), 130.56 (2C), 129.99 (2C), 129.75 (2C), 128.79 (2C), 128.20, 52.75, 49.18, 15.75.HRMS (ESI): calcd for $C_{23}H_{18}ClN_3O_3$ {[M+H]$^+$},420.1109; found, 420.1113.

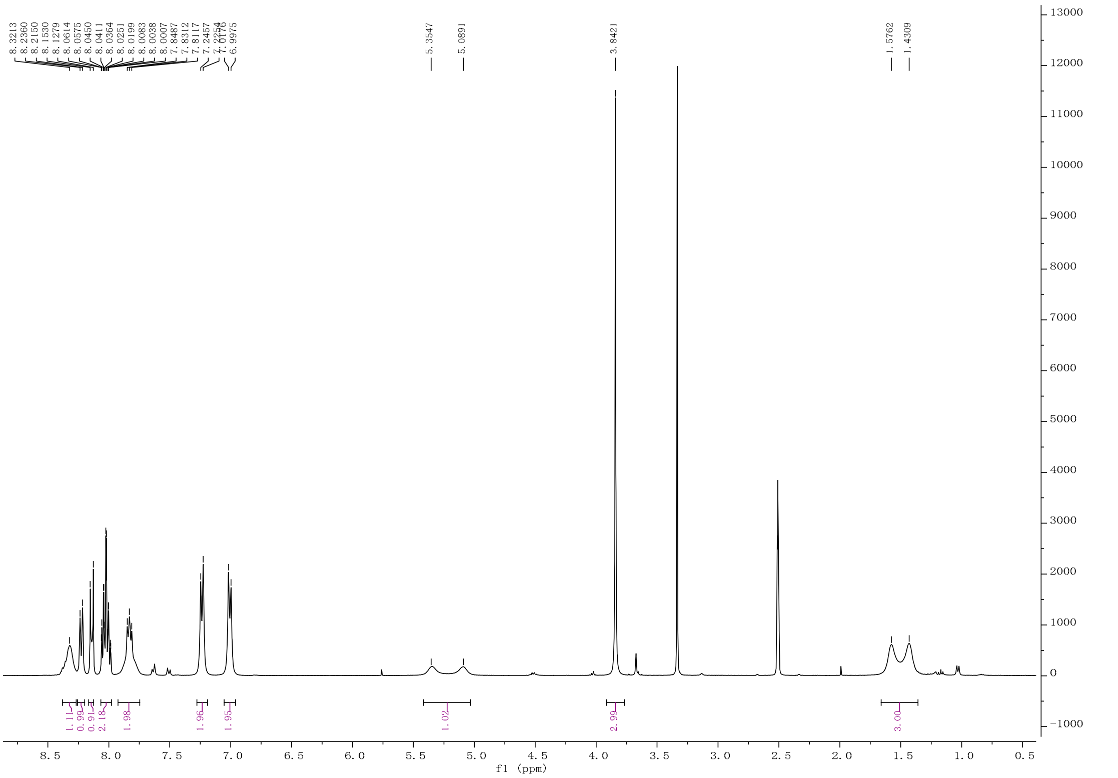

The ^{1}H NMR spectrogram of compound **E5**

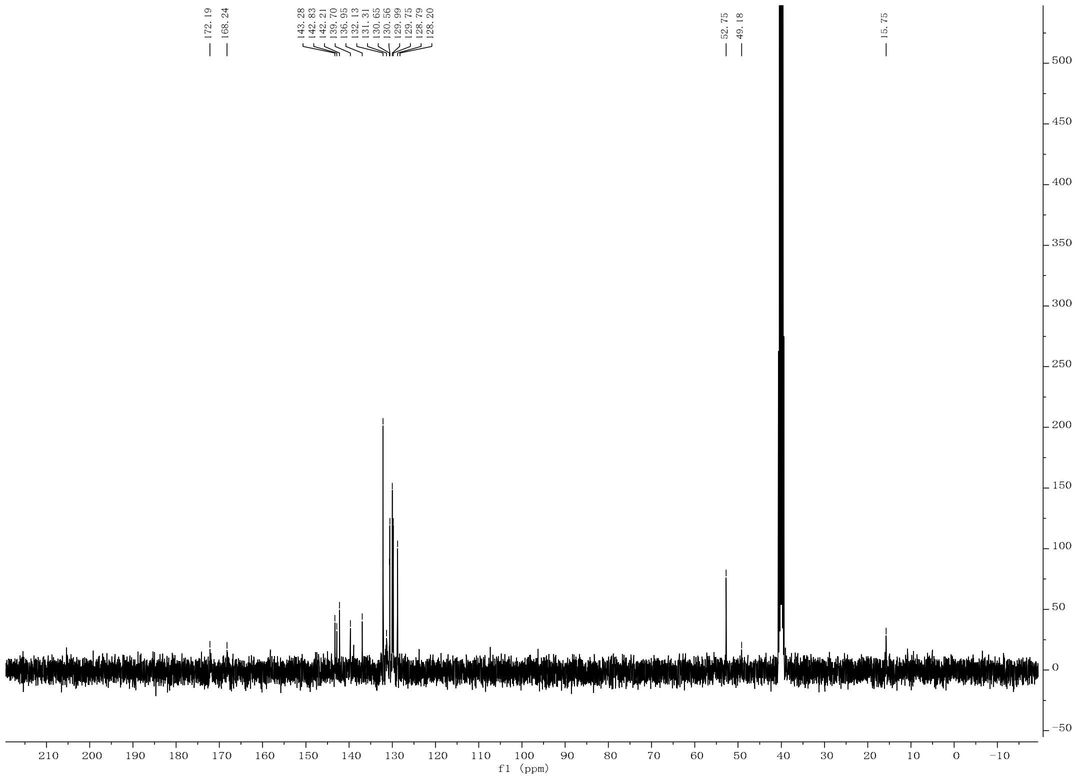

The ^{13}C NMR spectrogram of compound **E5**

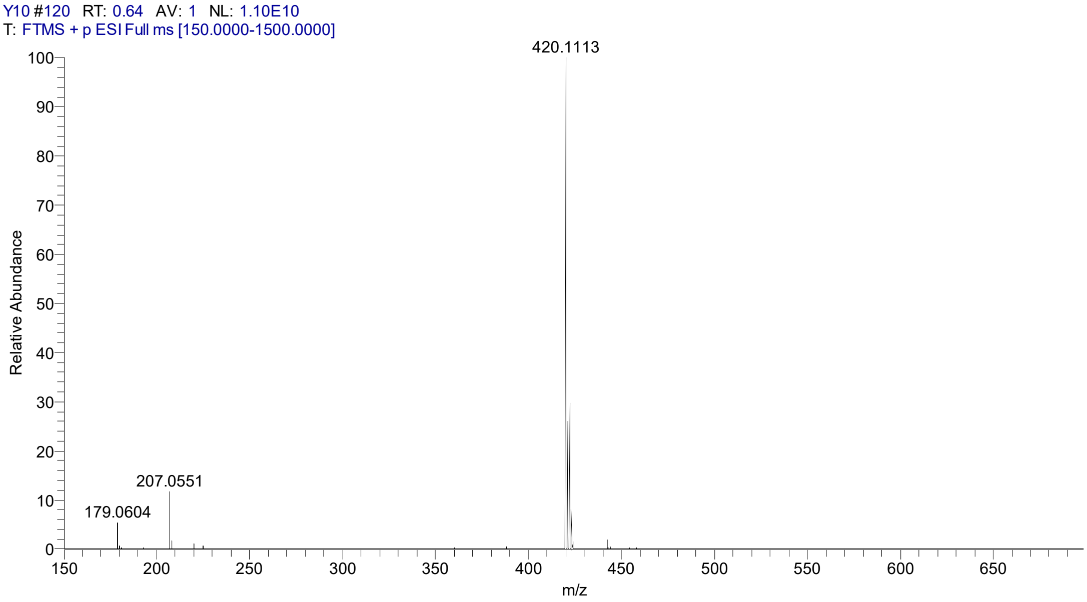

The HRMS spectrogram of compound **E5**

Compound **E6**,
methyl *N*-(3-chlorophenyl)-*N*-(phenazine-1-carbonyl)alaninate

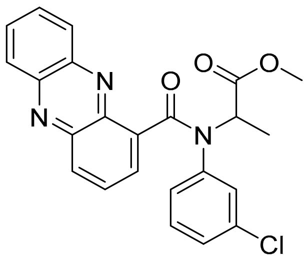

Yellow solid, yield 81.0%, m.p. 144.7-146.6°C; ^{1}H NMR (400 MHz, DMSO-*d*$_6$) δ 8.34 (s, 1H), 8.21 (d, J = 8.3 Hz, 1H), 8.14 (d, J = 9.8 Hz, 1H), 8.02 (dt, J = 15.7, 6.7 Hz, 2H), 7.96 – 7.78 (m, 2H), 7.37 (s, 1H), 7.14 (d, J = 7.1 Hz, 1H), 6.93 (d, J = 6.4 Hz, 2H), 5.21 (1H, two isomers), 3.85 (s, 3H), 1.53 (3H, two isomers). ^{13}C NMR (101 MHz, DMSO-*d*$_6$) δ 171.95, 168.14, 143.22, 142.76, 142.16, 142.11, 139.51, 136.86, 134.79, 132.56, 132.13 (2C), 130.80, 130.56, 130.08, 129.90, 129.71, 128.22, 123.02, 119.83, 57.02, 52.75, 15.76. HRMS (ESI): calcd for $C_{23}H_{18}ClN_3O_3$ {[M+H]$^+$}, 420.1109; found, 420.1110.

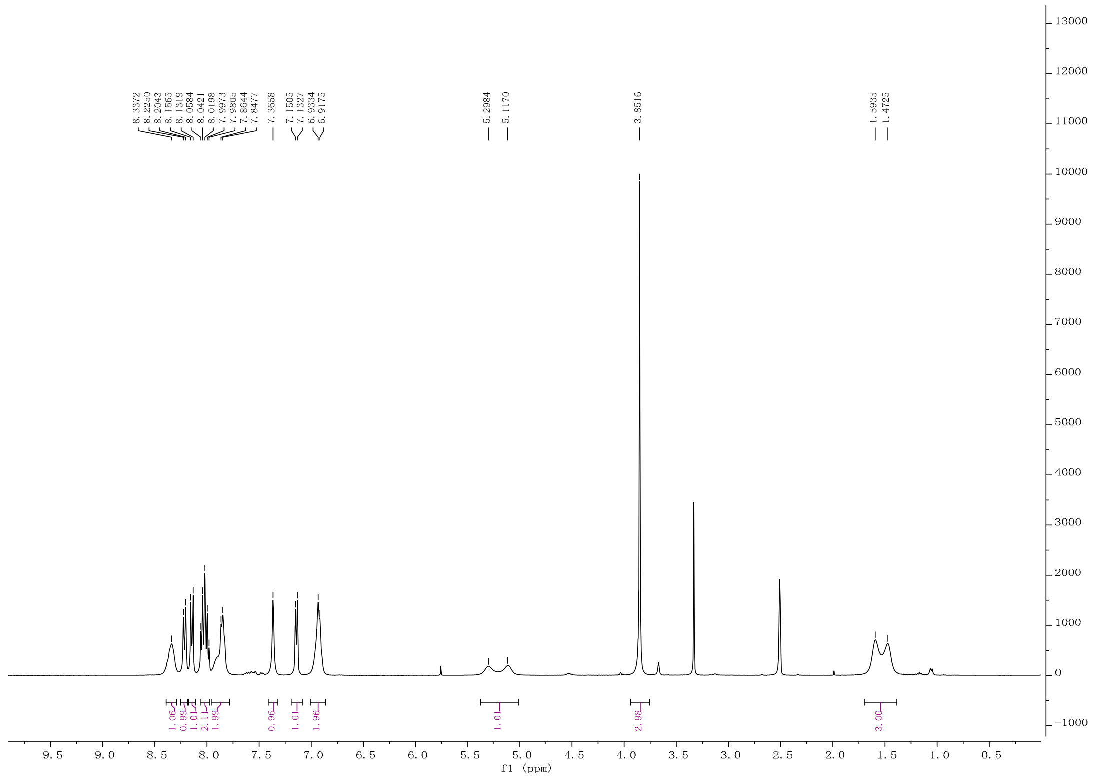

The ^{1}H NMR spectrogram of compound **E6**

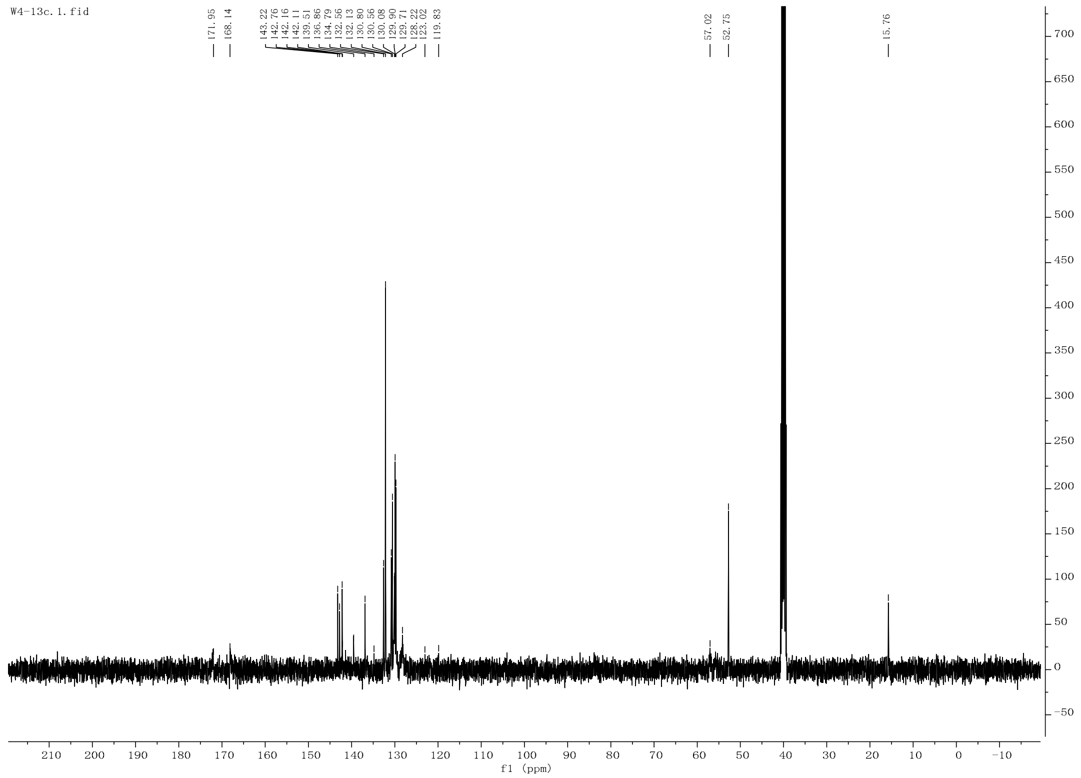

The ^{13}C NMR spectrogram of compound **E6**

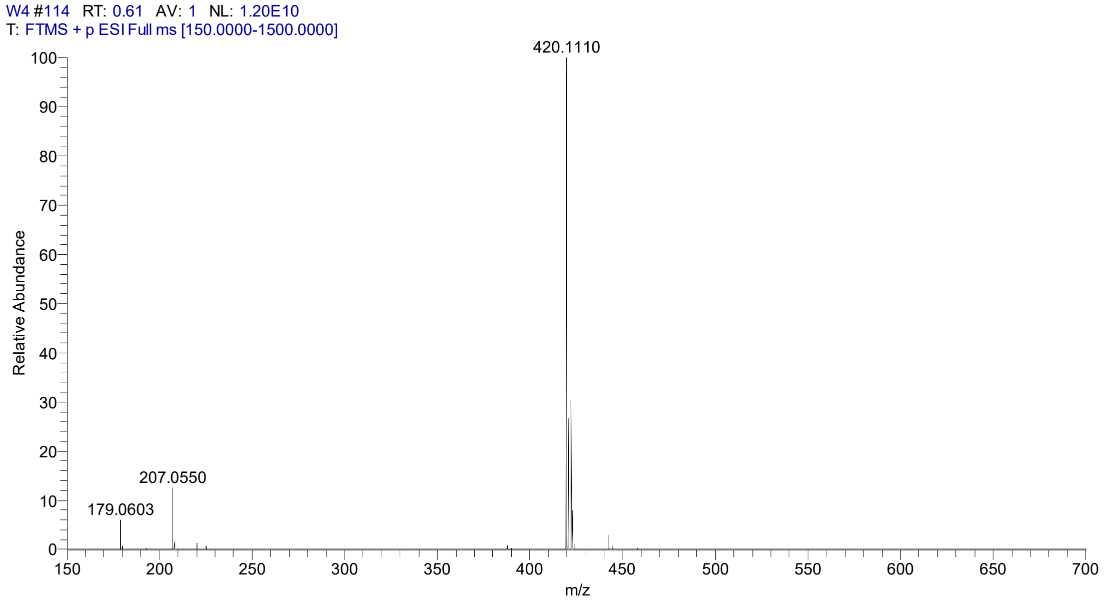

The HRMS spectrogram of compound **E6**

Compound **E7**,
methyl *N*-(4-chlorophenyl)-*N*-(phenazine-1-carbonyl)alaninate

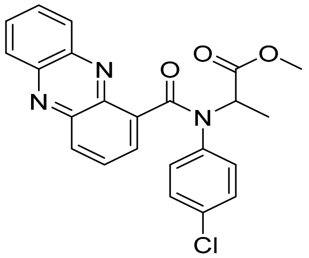

Yellow solid, yield 82.2%, m.p. 154.1-155.0°C; ^{1}H NMR (400 MHz, DMSO-*d*6) *δ* 8.32 (s, 1H), 8.22 (d, *J* = 8.5 Hz, 1H), 8.17 – 8.10 (m, 1H), 8.08 – 7.96 (m, 2H), 7.92 – 7.74 (m, 2H), 7.23 (s, 2H), 7.00 (d, *J* = 7.1 Hz, 2H), 5.11 (1H, two isomers), 3.84 (s, 3H), 1.51 (3H, two isomers). ^{13}C NMR (101 MHz, DMSO-*d*6) *δ* 175.03, 168.09, 143.28, 142.85, 142.18, 139.71, 136.96, 132.11, 131.49 (2C), 131.18, 130.65 (2C), 130.55, 129.98 (2C), 129.75, 128.78, 123.72 (2C), 56.32, 52.74, 15.77. HRMS (ESI): calcd for $C_{23}H_{18}ClN_3O_3$ {[M+H]$^+$}, 420.1109; found, 420.1111.

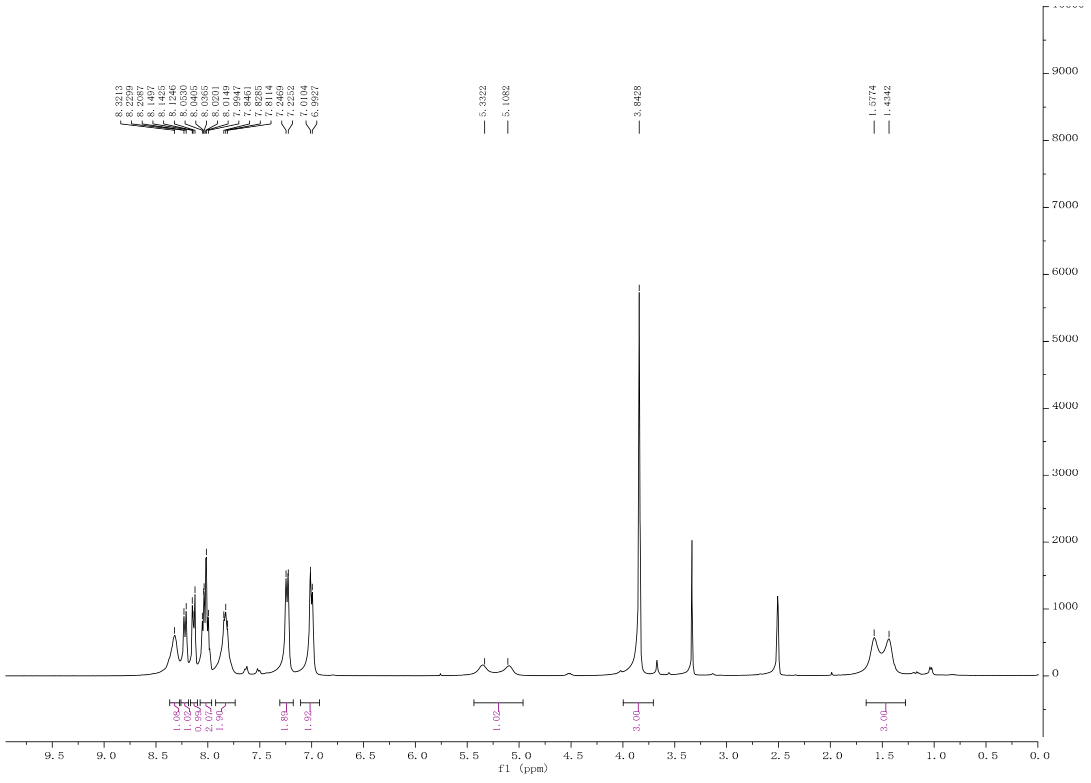

The ^{1}H NMR spectrogram of compound **E7**

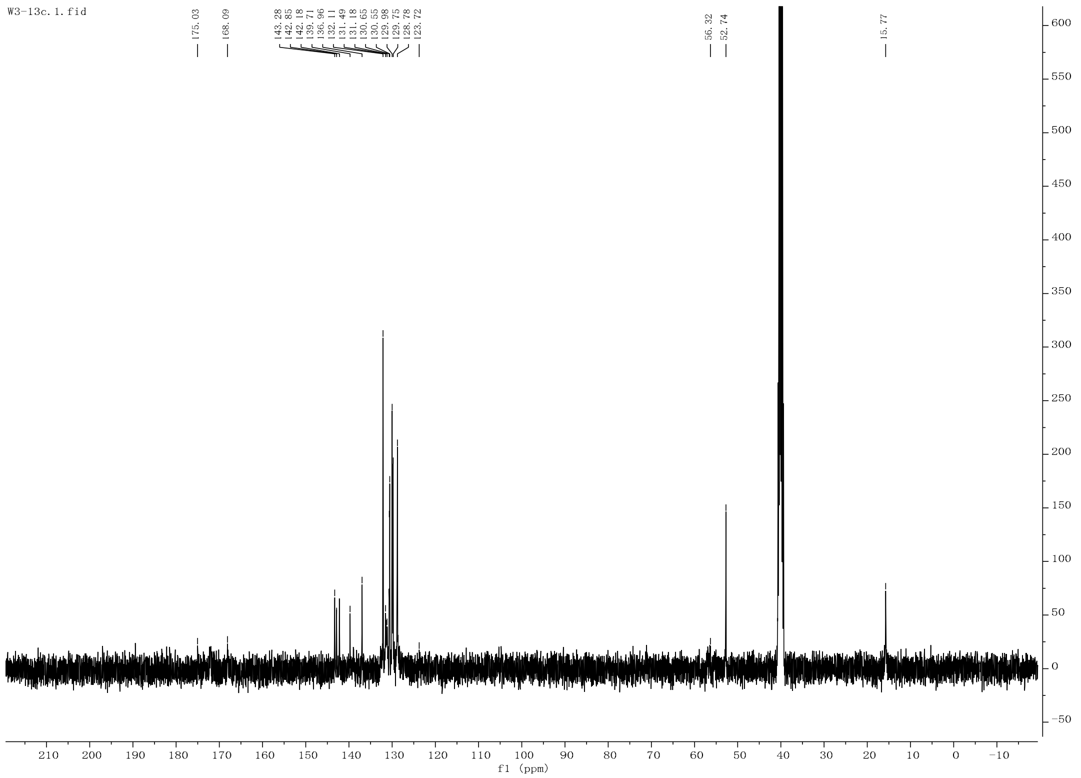

The ^{13}C NMR spectrogram of compound **E7**

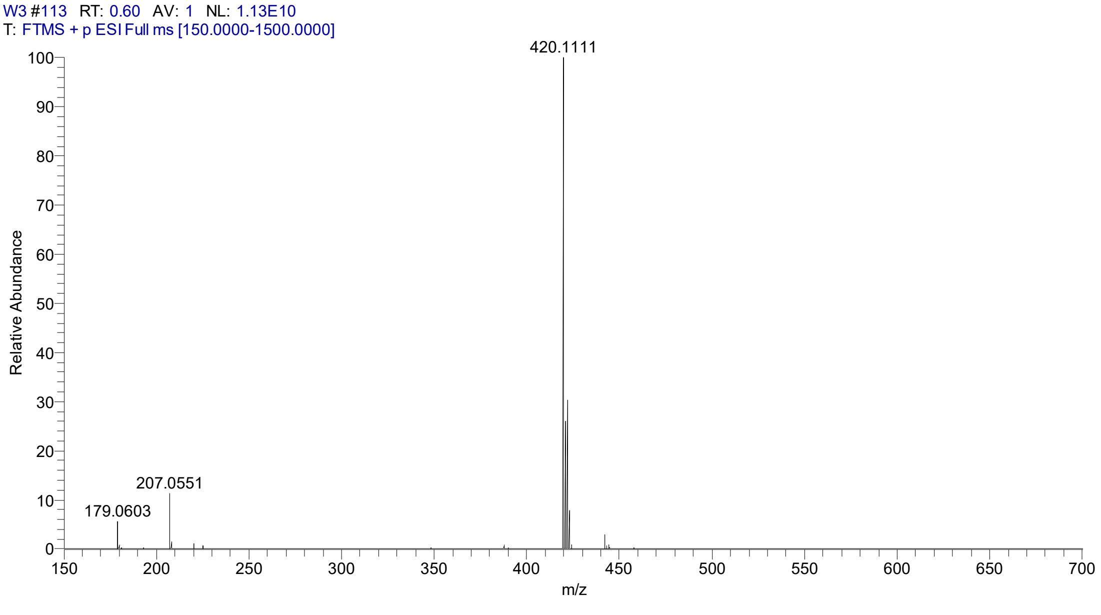

The HRMS spectrogram of compound **E7**

Compound **E8**,
methyl *N*-(3-bromophenyl)-*N*-(phenazine-1-carbonyl)alaninate

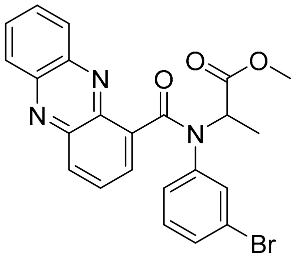

Yellow solid, yield 77.9%, m.p. 135.0-137.0℃;^{1}H NMR (400 MHz, DMSO-d_6) δ 8.39 – 8.30 (m, 1H), 8.22 (d, J = 8.3 Hz, 1H), 8.14 (d, J = 10.0 Hz, 1H), 8.06 – 7.98 (m, 2H), 7.94 – 7.78 (m, 2H), 7.50 (s, 1H), 7.26 – 7.14 (m, 1H), 7.07 (s, 1H), 6.86 (t, J = 7.5 Hz, 1H), 5.20 (1H, two isomers), 3.85 (s, 3H), 1.53 (3H, two isomers). ^{13}C NMR (101 MHz, DMSO-d_6) δ 172.22, 168.12, 143.22, 142.75, 142.15, 139.51, 136.86, 132.14 (2C), 131.05, 130.81, 130.56, 130.35, 129.93, 129.71, 128.59, 120.77, 57.00, 52.76, 15.75. HRMS (ESI): calcd for $C_{23}H_{18}BrN_3O_3$ {[M+H]$^+$}, 464.0605; found, 464.0607.

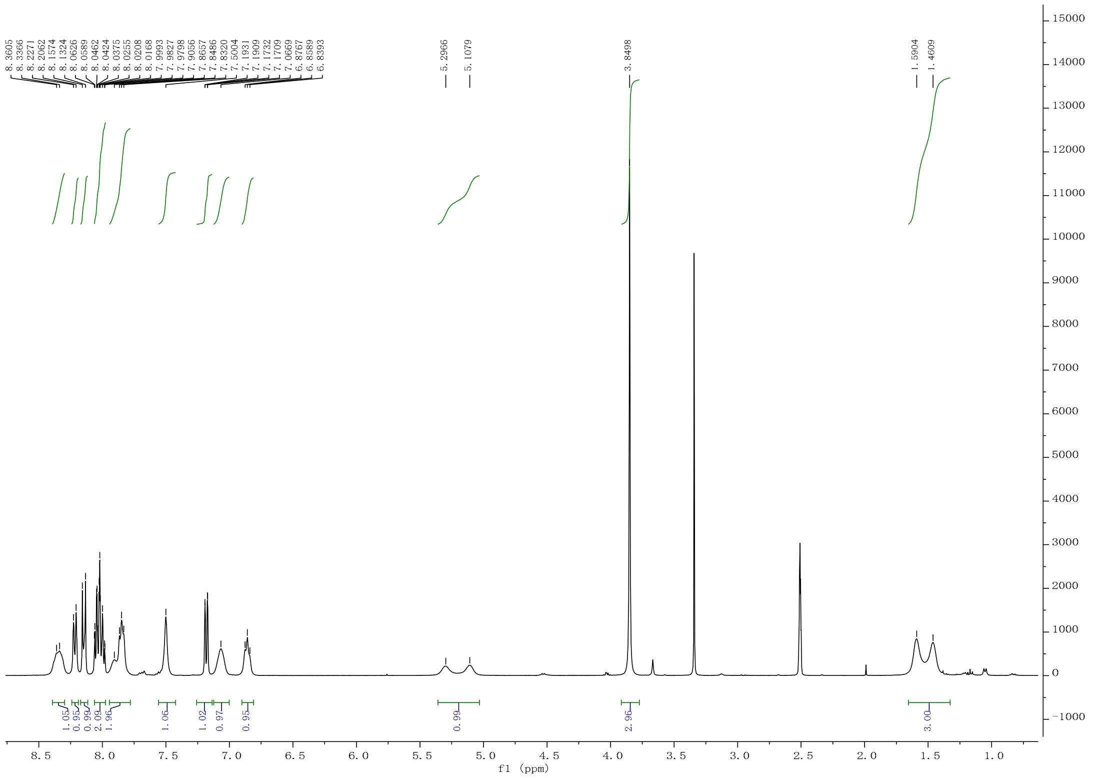

The ^{1}H NMR spectrogram of compound **E8**

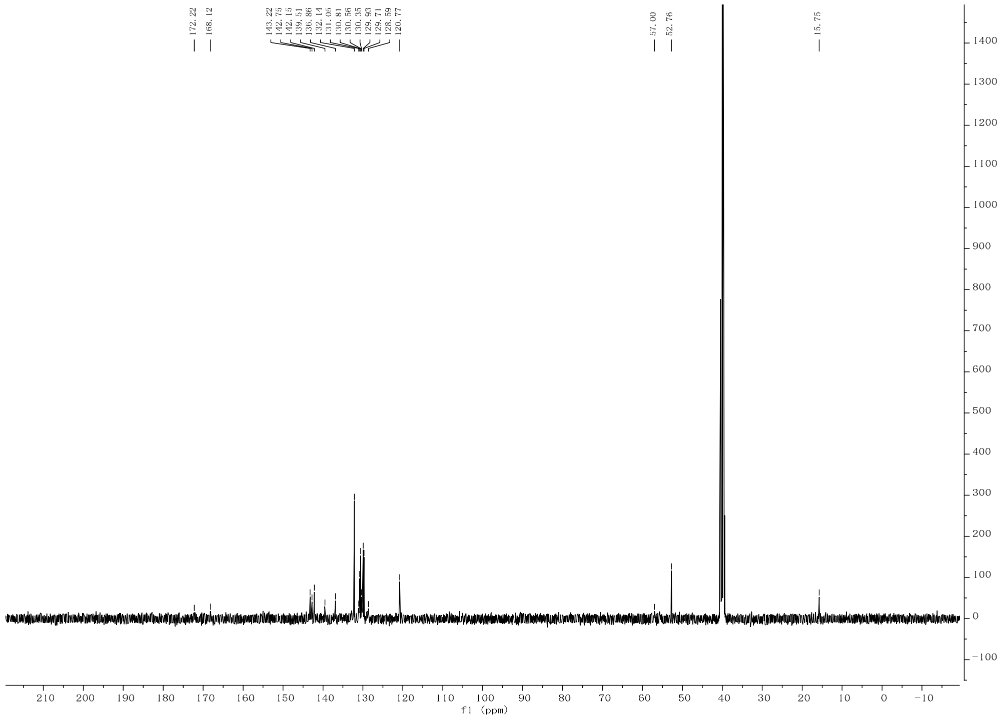

The ^{13}C NMR spectrogram of compound **E8**

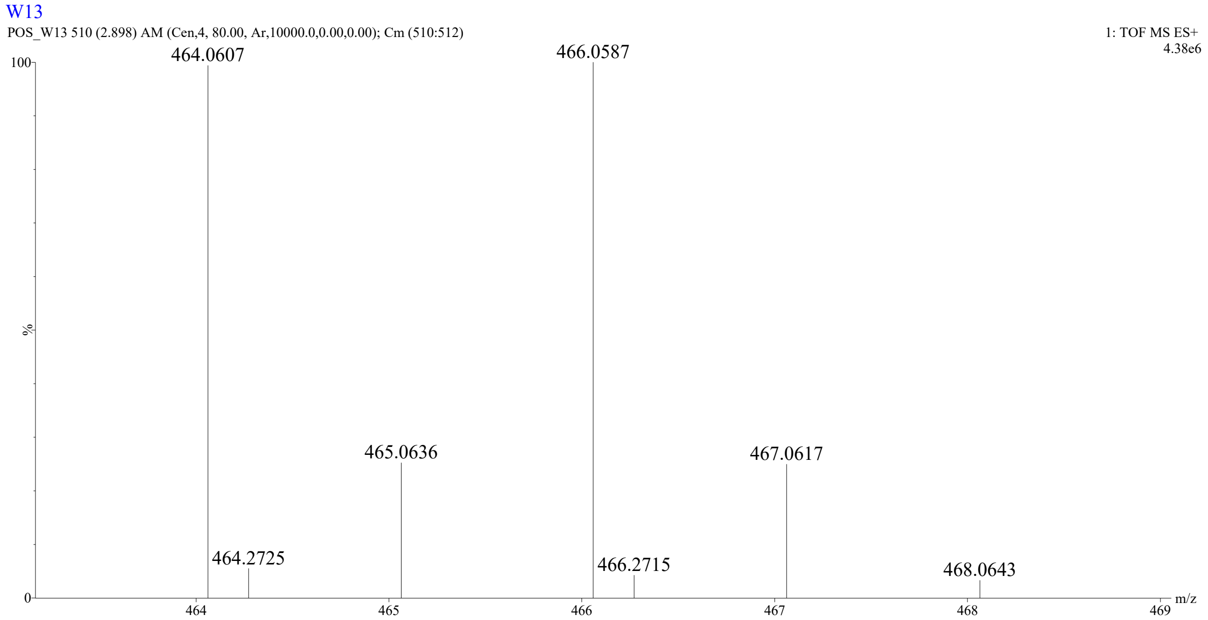

The HRMS spectrogram of compound **E8**

Compound **E9**,
methyl *N*-(4-bromophenyl)-*N*-(phenazine-1-carbonyl)alaninate

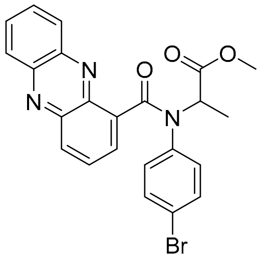

Yellow solid, yield 80.4%, m.p. 1172.0-173.8°C; ^{1}H NMR (400 MHz, DMSO-d_6) δ 8.32 (s, 1H), 8.22 (d, J = 8.0 Hz, 1H), 8.14 (d, J = 10.1 Hz, 1H), 8.06 – 7.98 (m, 2H), 7.89 – 7.75 (m, 2H), 7.16 (q, J = 8.7 Hz, 4H), 5.21 (1H, two isomers), 3.84 (s, 3H), 1.51 (3H, two isomers). ^{13}C NMR (101 MHz, DMSO-d_6) δ 167.93, 166.12, 143.29, 142.85, 142.21, 139.72, 136.93, 135.18, 132.12 （2C）, 131.76, 131.37 (2C), 130.66 (2C), 130.56, 129.99, 129.75 (2C), 85.78, 52.74, 15.70. HRMS (ESI): calcd for $C_{23}H_{18}BrN_3O_3$ {[M+H]$^+$}, 464.0604; found, 464.0612.

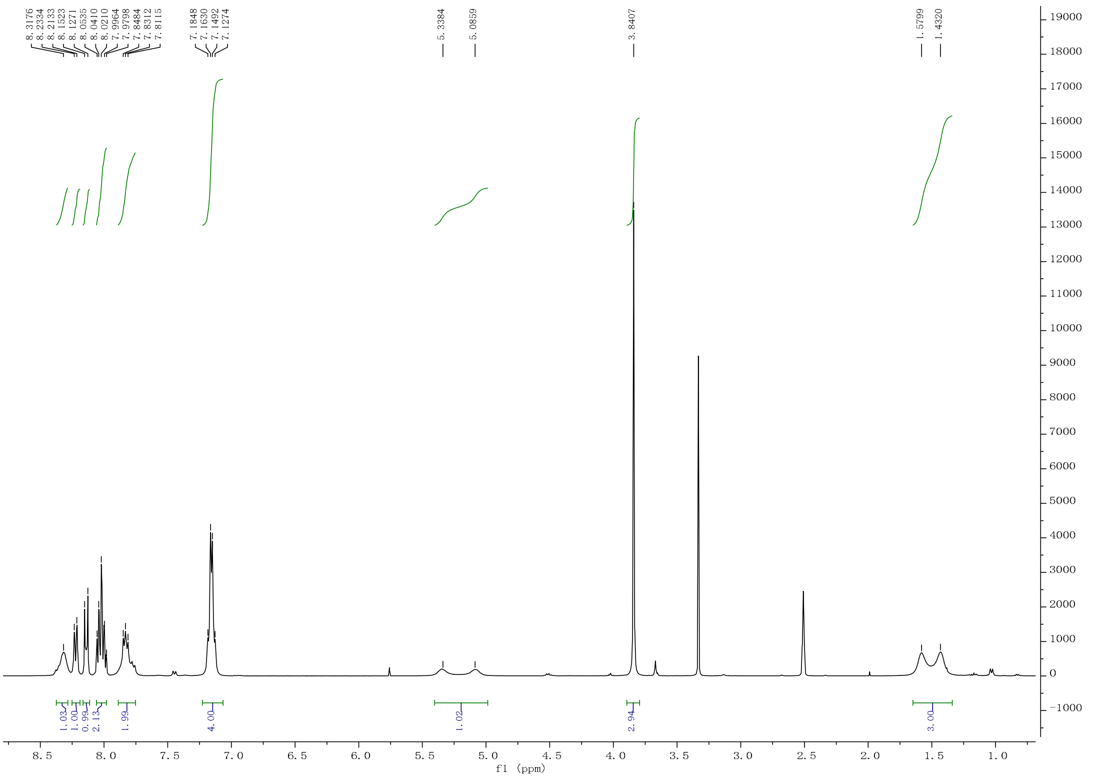

The ^{1}H NMR spectrogram of compound **E9**

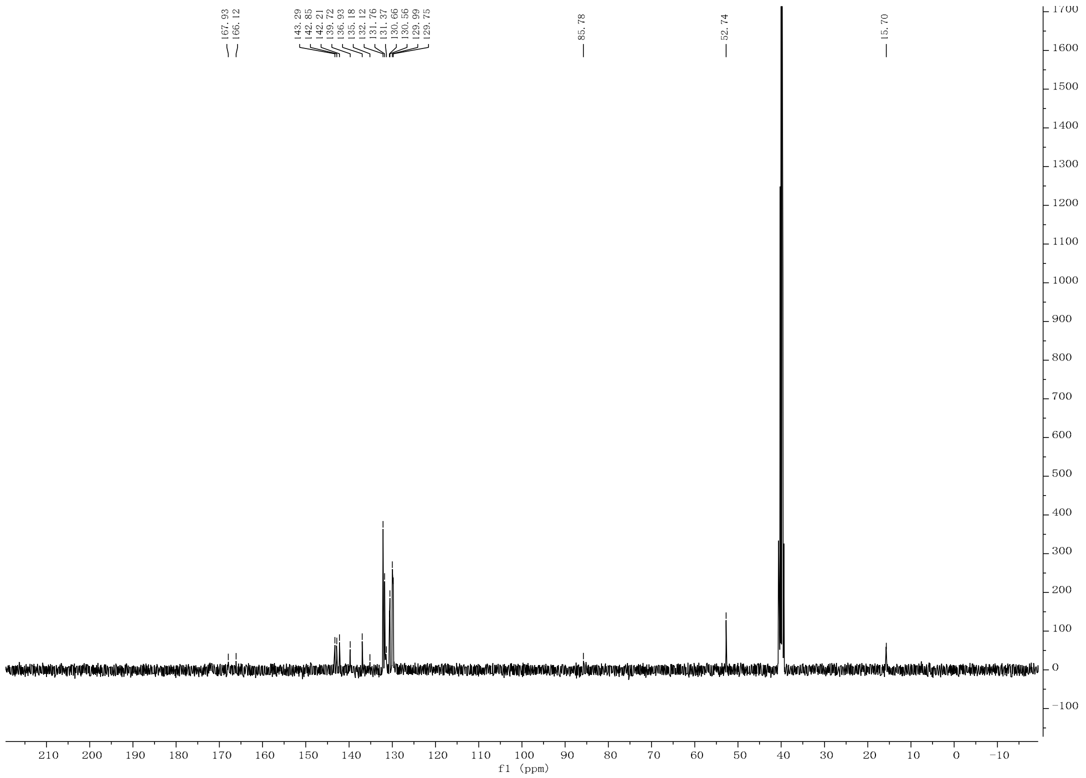

The ^{13}C NMR spectrogram of compound **E9**

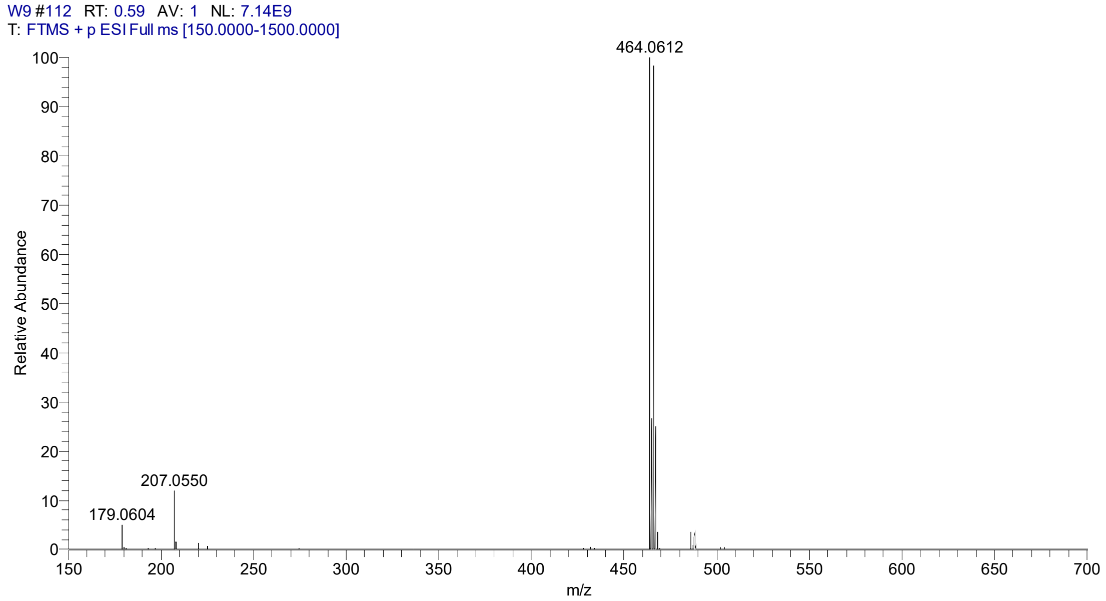

The HRMS spectrogram of compound **E9**

Compound **E10**,

methyl *N*-(phenazine-1-carbonyl)-*N*-(m-tolyl)alaninate

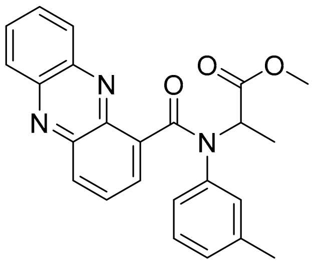

Brown solid, yield 81.1%, m.p. 168.3-170.1°C; ^{1}H NMR (400 MHz, DMSO-d_6) δ 8.34 (d, J = 7.0 Hz, 1H), 8.20 (d, J = 8.0 Hz, 1H), 8.13 – 8.06 (m, 1H), 8.05 – 7.96 (m, 2H), 7.81 (s, 2H), 7.10 (s, 1H), 6.93 (d, J = 7.9 Hz, 1H), 6.74 (t, J = 7.6 Hz, 1H), 6.67 (s, 1H), 5.17 (1H, two isomers), 3.84 (s, 3H), 1.81 (s, 3H), 1.52 (3H, two isomers). ^{13}C NMR (101 MHz, DMSO-d_6) δ: 172.02, 168.19, 143.14, 142.73, 142.20, 139.80, 137.94, 137.39, 131.97, 130.50 (2C), 130.41 (2C), 129.89, 129.71 (2C), 128.30, 126.81, 120.84, 120.51, 52.64, 20.78, 15.76. HRMS (ESI): calcd for $C_{24}H_{21}N_3O_3$ {[M+H]$^+$}, 400.1656; found, 400.1657.

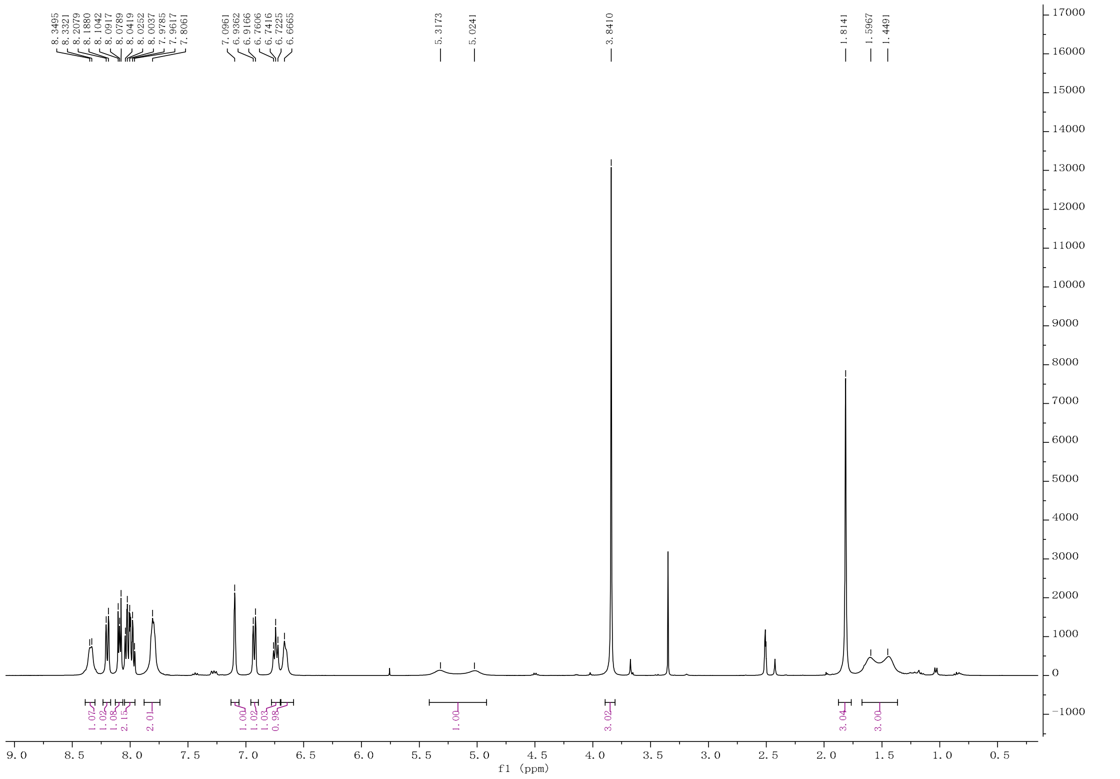

The ^{1}H NMR spectrogram of compound **E10**

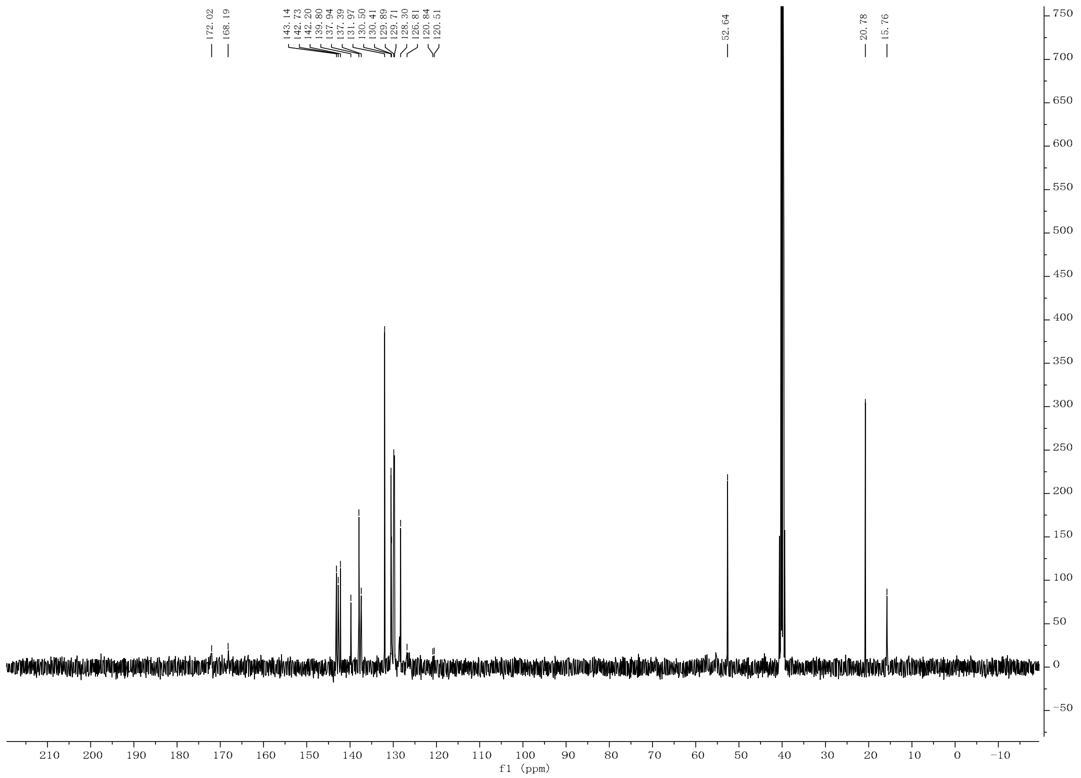

The ^{13}C NMR spectrogram of compound **E10**

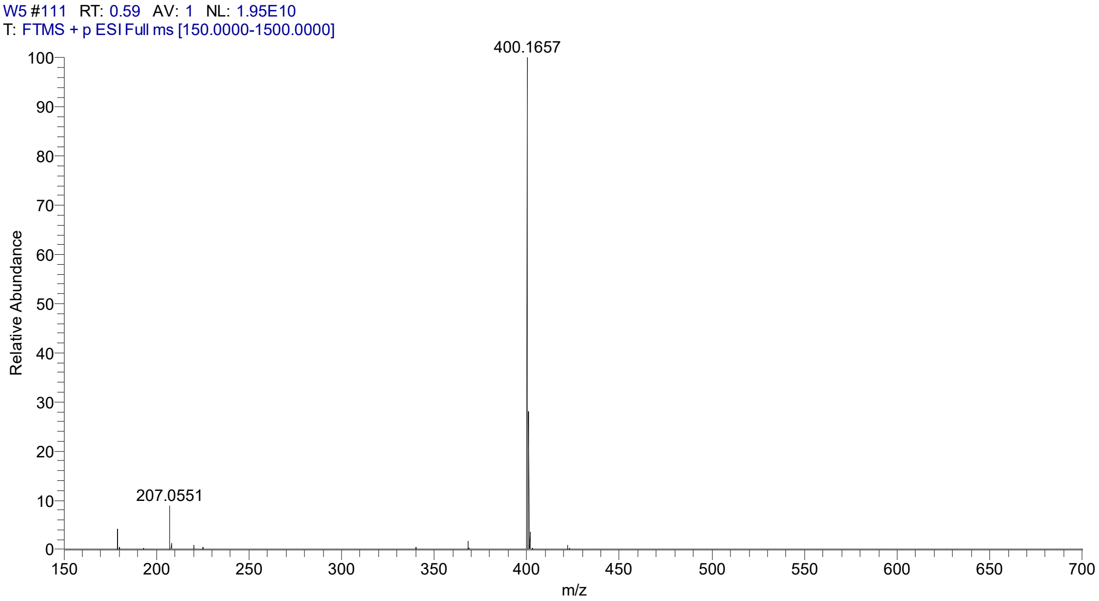

The HRMS spectrogram of compound **E10**

Compound **E11**,

methyl N-(3-ethylphenyl)-N-(phenazine-1-carbonyl)alaninate

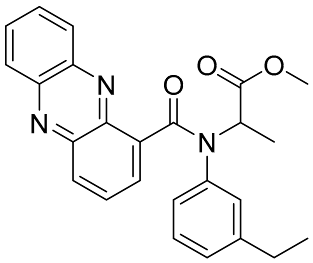

Yellow solid, yield 79.6%, m.p. 111.9-112.9°C; ^{1}H NMR (400 MHz, DMSO-d_6) δ 8.34 (s, 1H), 8.19 (d, J = 8.4 Hz, 1H), 8.10 – 7.95 (m, 3H), 7.87 – 7.70 (m, 2H), 7.00 (d, J = 8.5 Hz, 2H), 6.83 (s, 1H), 6.66 (s, 1H), 5.37 (1H, two isomers), 3.84 (s, 3H), 2.06 (q, J = 7.5 Hz, 2H), 1.52 (3H, two isomers), 0.38 (s, 3H).^{13}C NMR (101 MHz, DMSO-d_6) δ 171.99, 168.21, 144.17, 143.17, 142.78, 142.20, 139.83, 137.47, 131.94, 130.47, 130.33, 129.90, 129.68, 129.35, 129.15, 128.47 (2C), 127.58, 126.84, 126.56, 55.36, 52.63, 27.84, 15.79, 15.11.HRMS (ESI): calcd for $C_{25}H_{23}N_3O_3$ {[M+H]$^+$}, 414.1812; found, 414.1814.

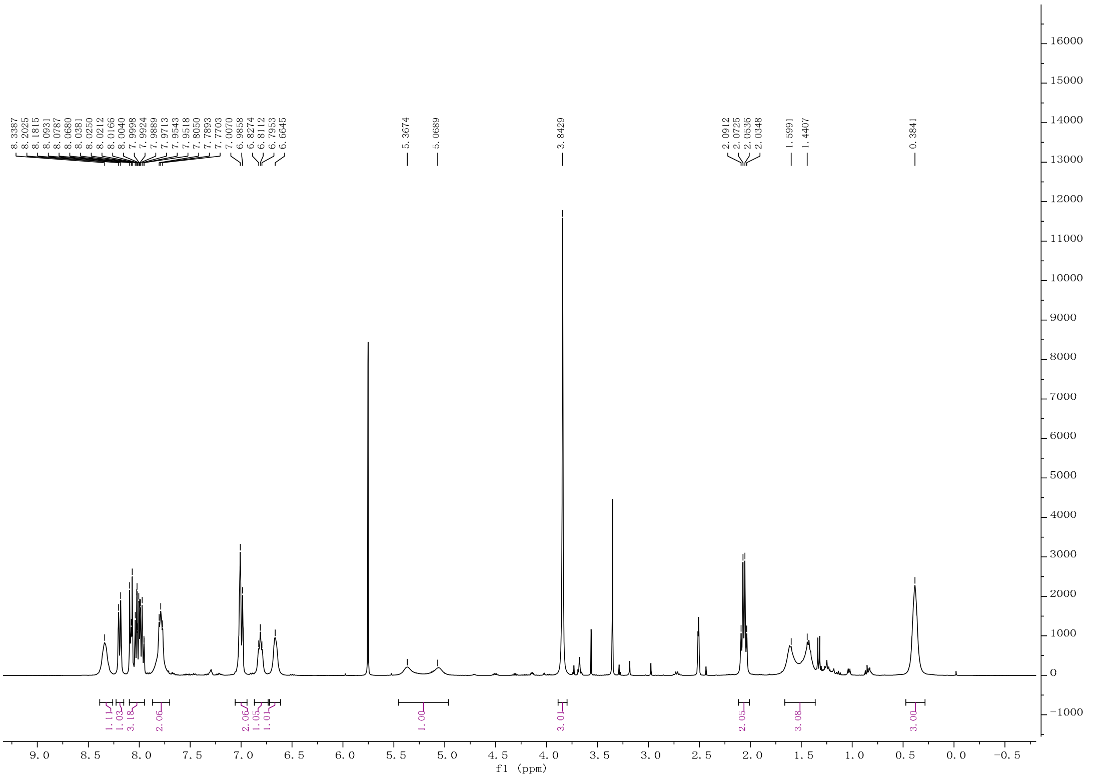

The ^{1}H NMR spectrogram of compound **E11**

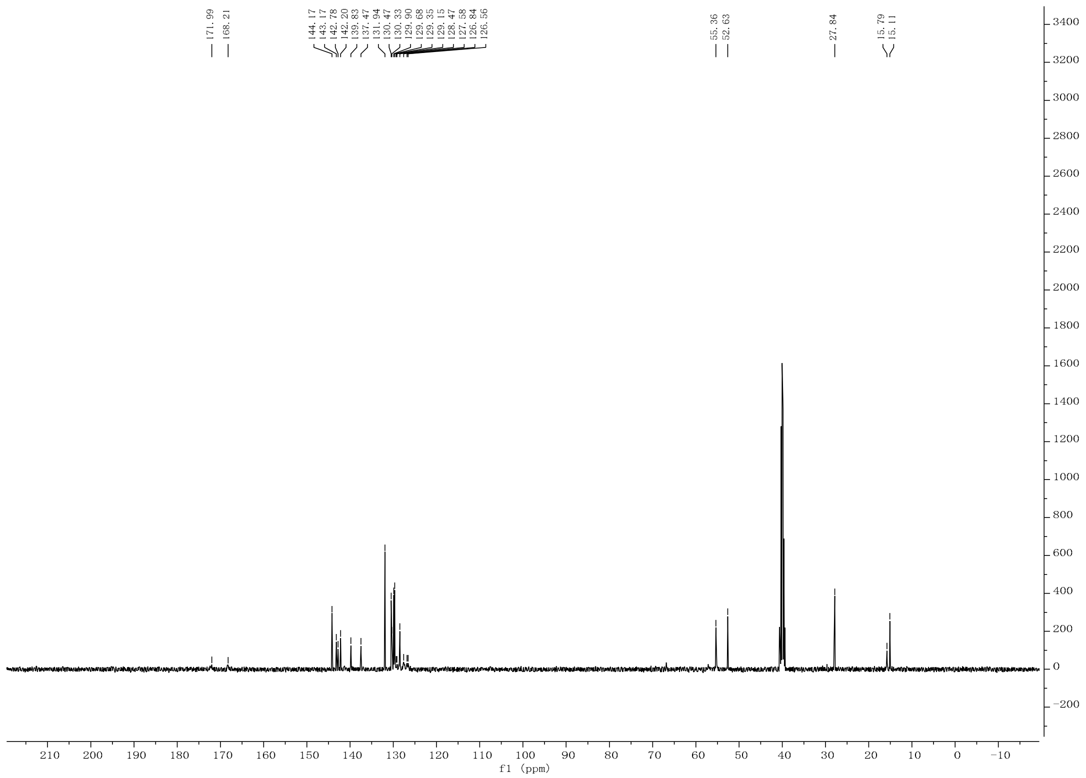

The ^{13}C NMR spectrogram of compound **E11**

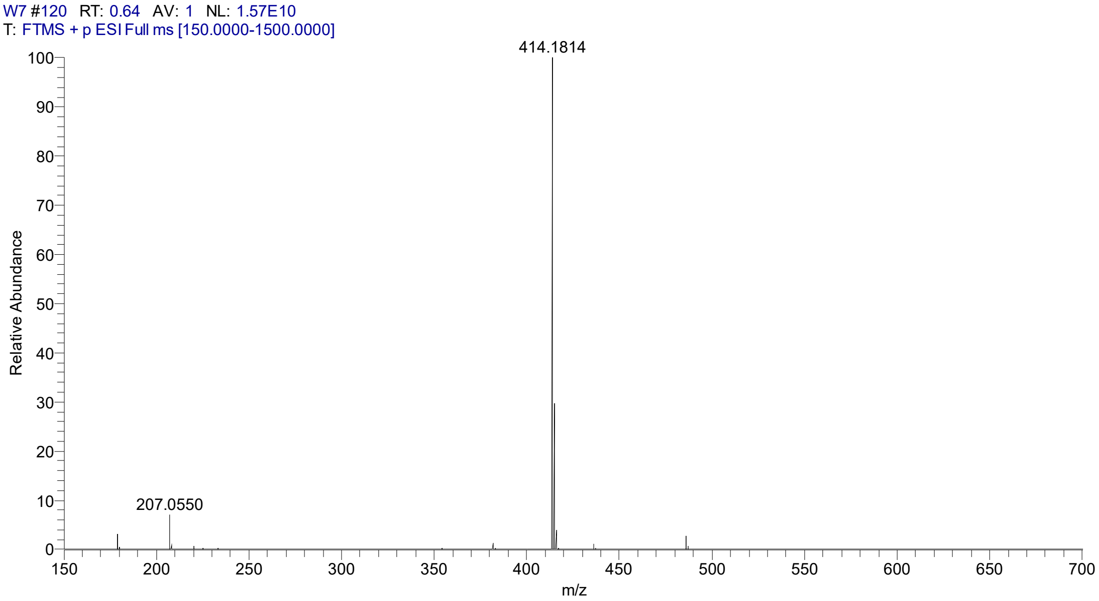

The HRMS spectrogram of compound **E11**

Compound **E12**,

methyl *N*-(4-ethylphenyl)-*N*-(phenazine-1-carbonyl)alaninate

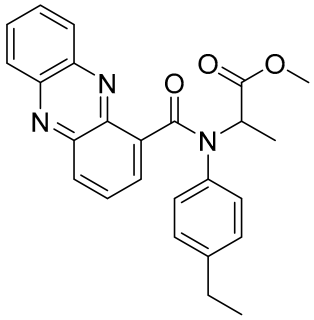

Yellow solid, yield 83.4%, m.p. 136.5-138.3°C; ^{1}H NMR (400 MHz, DMSO-*d*$_6$) δ 8.32 (s, 1H), 8.22 (d, J = 7.8 Hz, 1H), 8.10 (d, J = 10.0 Hz, 1H), 8.06 – 7.97 (m, 2H), 7.83 – 7.69 (m, 2H), 7.10 (d, J = 8.2 Hz, 2H), 6.75 (d, J = 7.9 Hz, 2H), 5.19 (1H, two isomers), 3.84 (s, 3H), 2.24 (q, J = 7.0 Hz, 2H), 1.50 (3H, two isomers), 0.85 (t, J = 7.5 Hz, 3H). ^{13}C NMR (101 MHz, DMSO-*d*$_6$) δ 168.25, 165.85, 143.21, 142.83, 142.24, 139.93, 138.77, 137.41, 132.03, 130.56, 130.30, 130.00 (2C), 129.72 (2C), 129.61, 128.00 (2C), 116.61, 57.34, 52.65, 27.71, 15.75, 15.38. HRMS (ESI): calcd for $C_{25}H_{23}N_3O_3$ {[M+H]$^+$}, 414.1813; found, 414.1818.

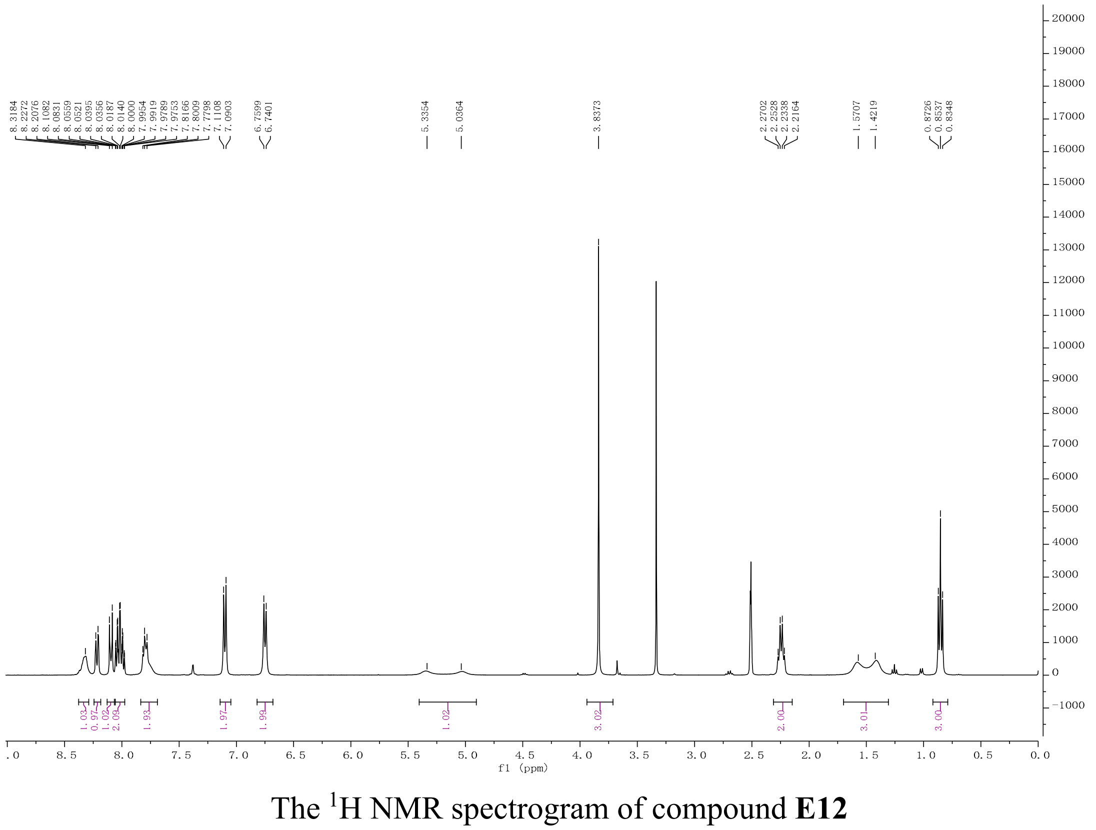

The ^{1}H NMR spectrogram of compound **E12**

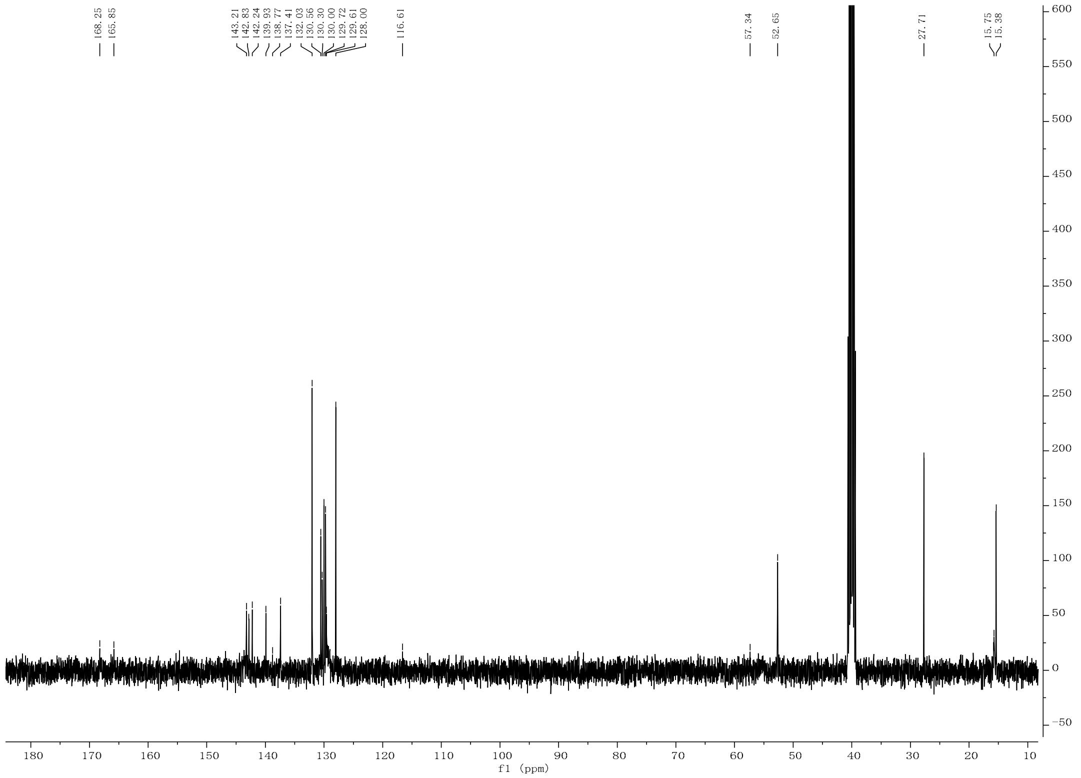

The ^{13}C NMR spectrogram of compound **E12**

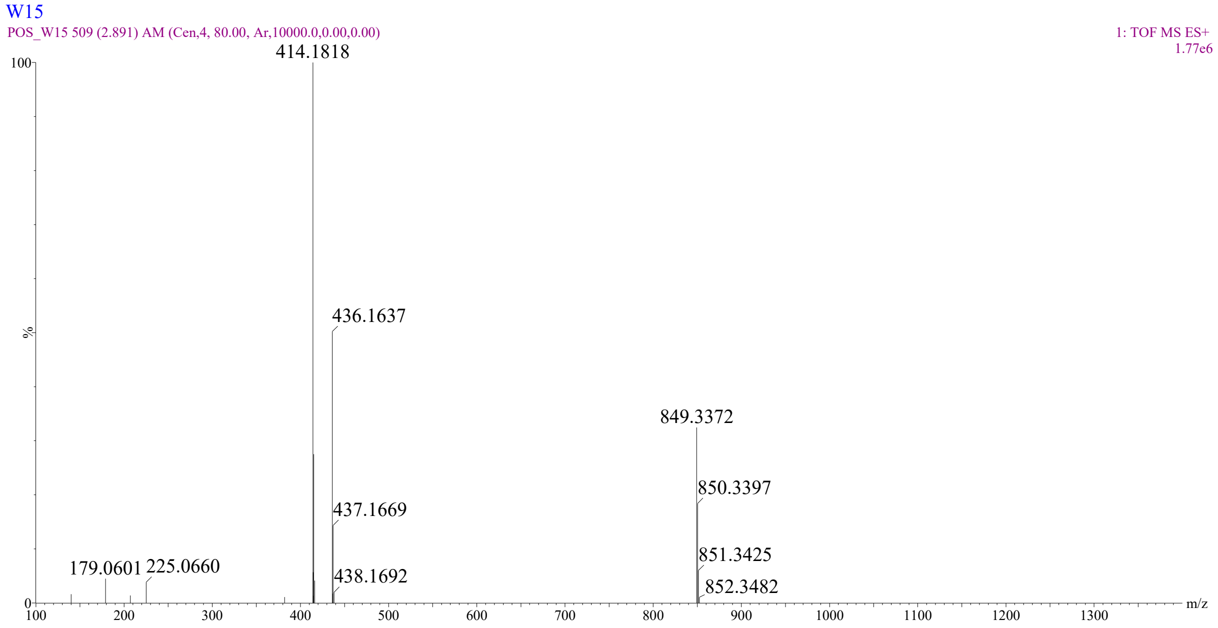

The HRMS spectrogram of compound **E12**

Compound **E13**,
methyl *N*-(3-isopropylphenyl)-*N*-(phenazine-1-carbonyl)alaninate

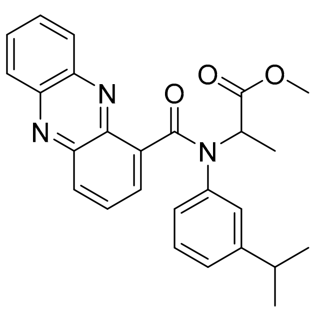

Yellow fluid, yield 83.4%; ^{1}H NMR (400 MHz, DMSO-d_6) δ 8.35 (s, 1H), 8.21 (d, J = 8.4 Hz, 1H), 8.10 – 7.97 (m, 3H), 7.85 – 7.72 (m, 2H), 7.05 – 6.93 (m, 2H), 6.87 (t, J = 6.5 Hz, 1H), 6.71 (s, 1H), 5.25 (1H, two isomers), 3.84 (s, 3H), 2.34 (p, J = 7.7, 7.3 Hz, 1H), 1.51 (3H, two isomers), 0.56 (s, 3H), 0.37 (s, 3H). ^{13}C NMR (101 MHz, DMSO-d_6) δ 172.04, 168.29, 148.69, 143.20, 142.84, 142.19, 139.85, 137.50, 132.00, 130.50, 130.26, 129.92 (2C), 129.68 (2C), 128.57, 127.70, 126.80, 126.40, 126.17, 54.96, 52.65, 32.96, 23.37, 15.85, 14.56. HRMS (ESI): calcd for $C_{26}H_{25}N_3O_3$ {[M+H]$^+$}, 428.1969; found, 428.1972.

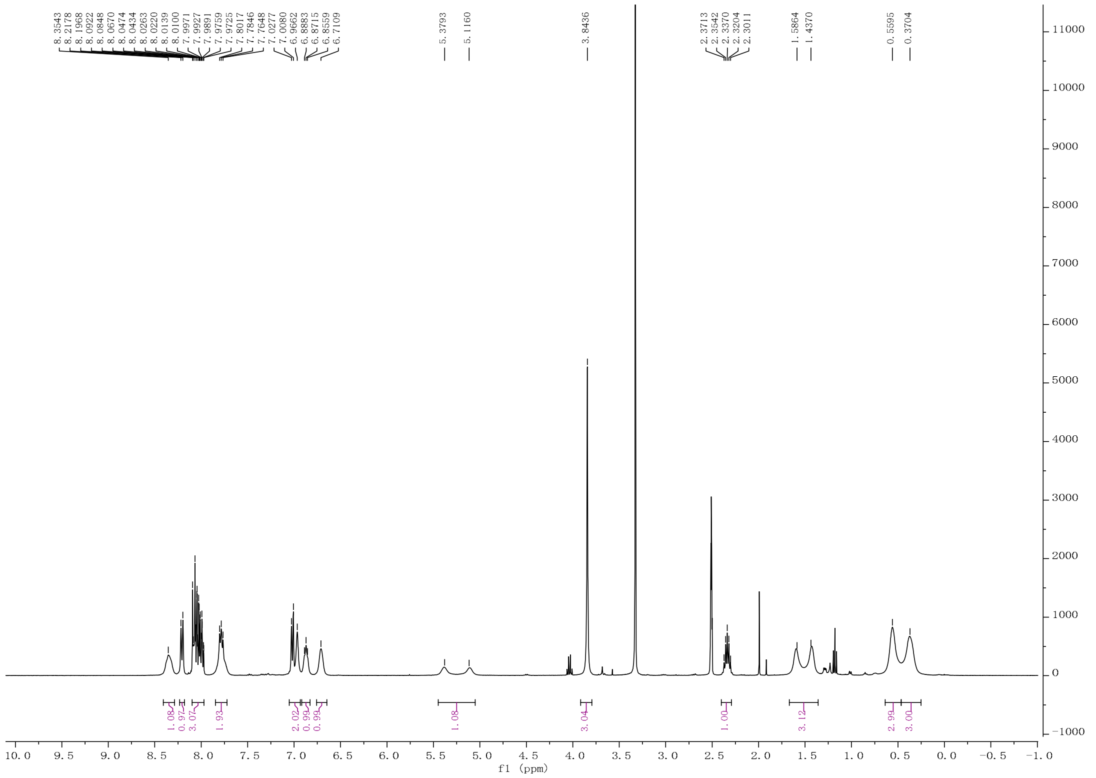

The ^{1}H NMR spectrogram of compound **E13**

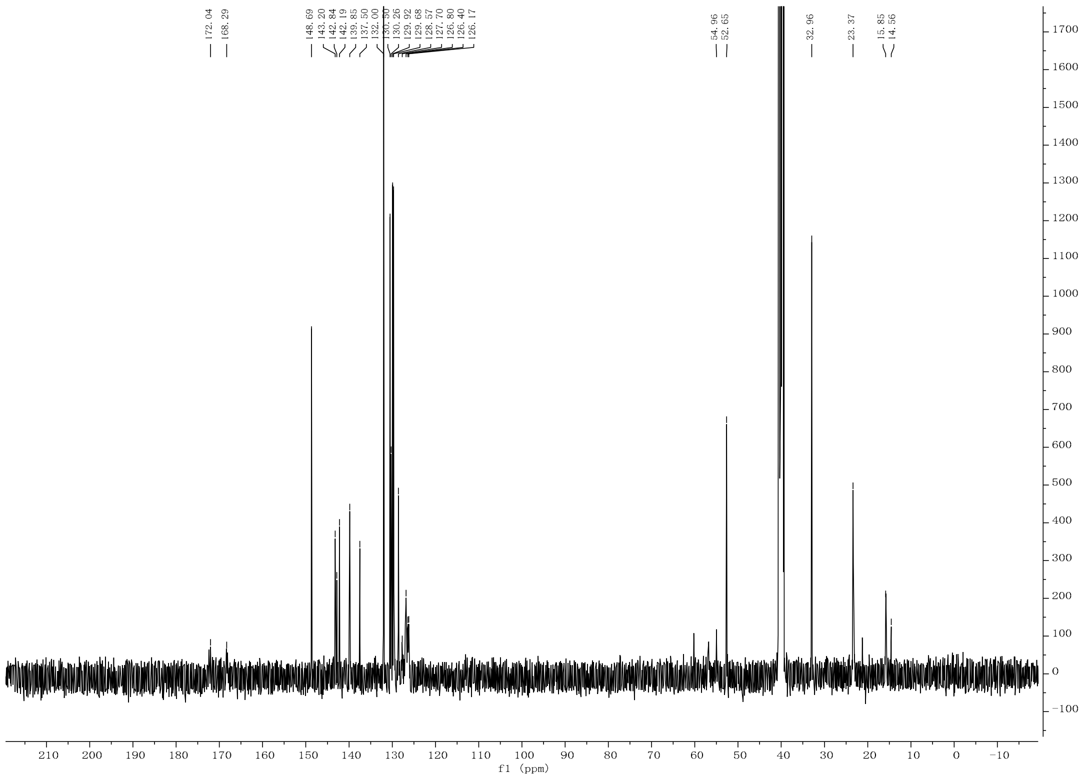

The ^{13}C NMR spectrogram of compound **E13**

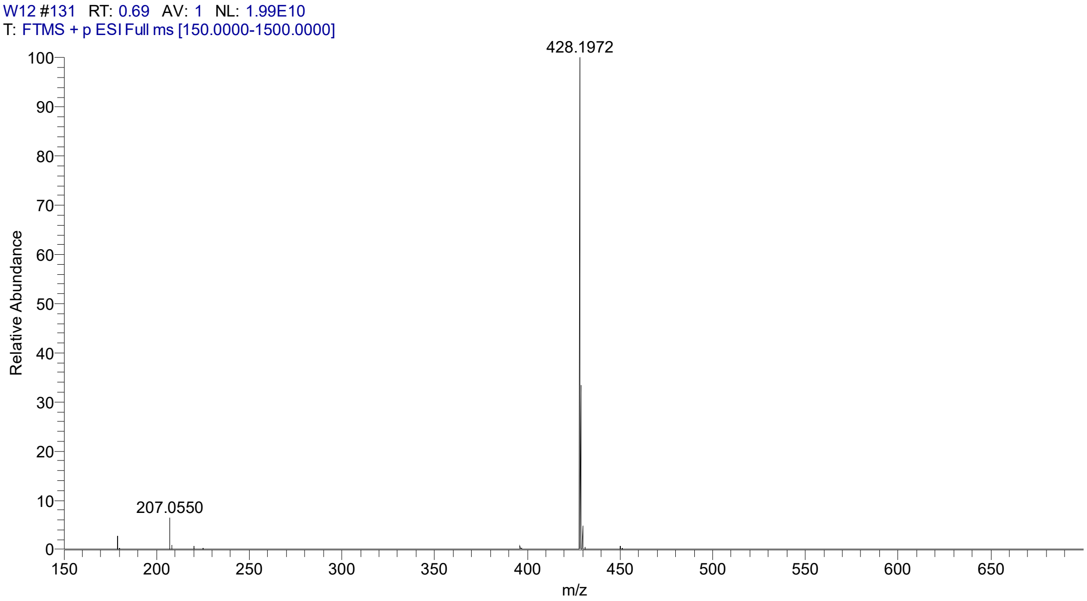

The HRMS spectrogram of compound **E13**

Compound **E14**,
methyl *N*-(4-isopropylphenyl)-*N*-(phenazine-1-carbonyl)alaninate

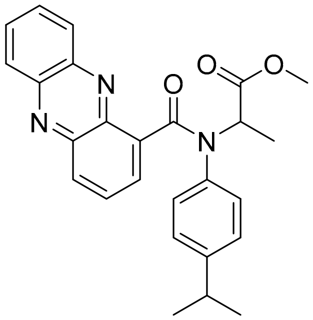

Yellow fluid, yield 82.7%; ^{1}H NMR (400 MHz, DMSO-d_6) δ 8.36 – 8.28 (m, 1H), 8.21 (d, *J* = 8.2 Hz, 1H), 8.12 – 8.06 (m, 1H), 8.01 (dddd, *J* = 16.3, 8.0, 6.6, 1.5 Hz, 2H), 7.86 – 7.66 (m, 2H), 7.09 (d, *J* = 8.4 Hz, 2H), 6.77 (d, *J* = 8.1 Hz, 2H), 5.17 (1H, two isomers), 3.84 (s, 3H), 2.54 (s, 1H), 1.51 (3H, two isomers), 0.85 (d, *J* = 6.9 Hz, 6H). ^{13}C NMR (101 MHz, DMSO-d_6) *δ* 172.26, 168.18, 148.11, 143.17, 142.79, 142.23, 139.93, 137.36, 131.98, 130.51 (2C), 130.30 (3C), 129.98 (2C), 129.68 (2C), 126.44 (2C), 55.34, 52.64, 32.97, 24.31, 23.77, 15.74. HRMS (ESI): calcd for $C_{26}H_{25}N_3O_3$ {[M+H]$^+$}, 428.1969; found, 428.1971.

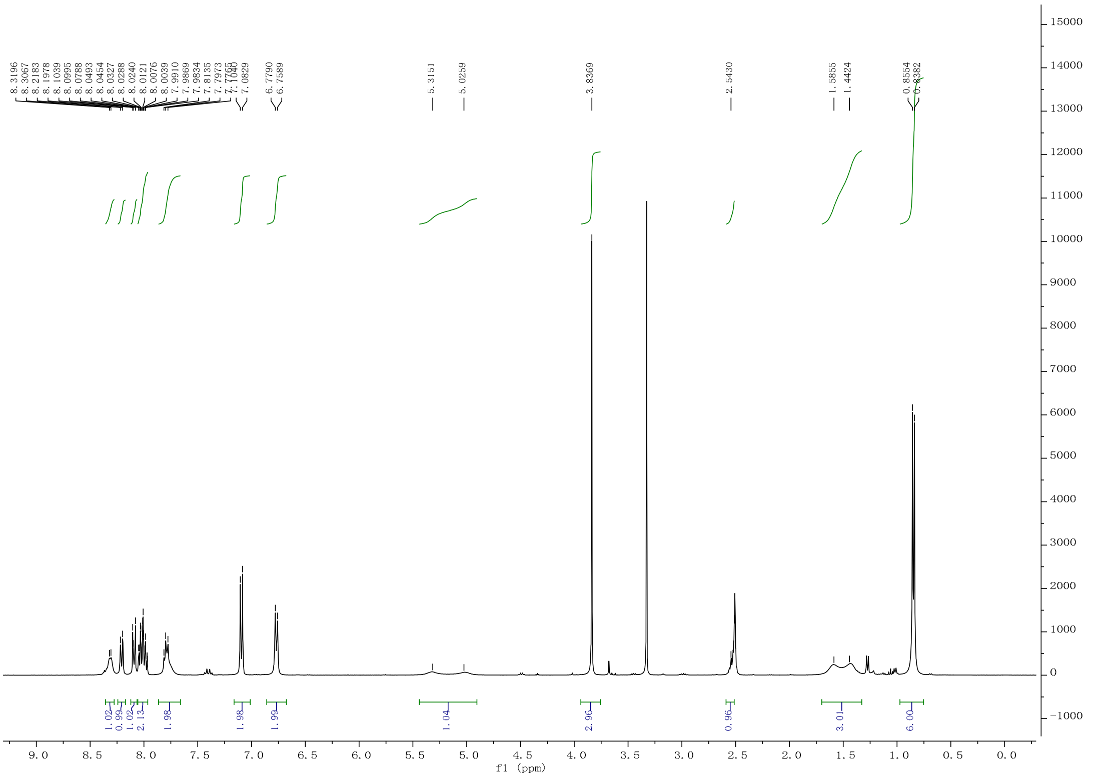

The ^{1}H NMR spectrogram of compound **E14**

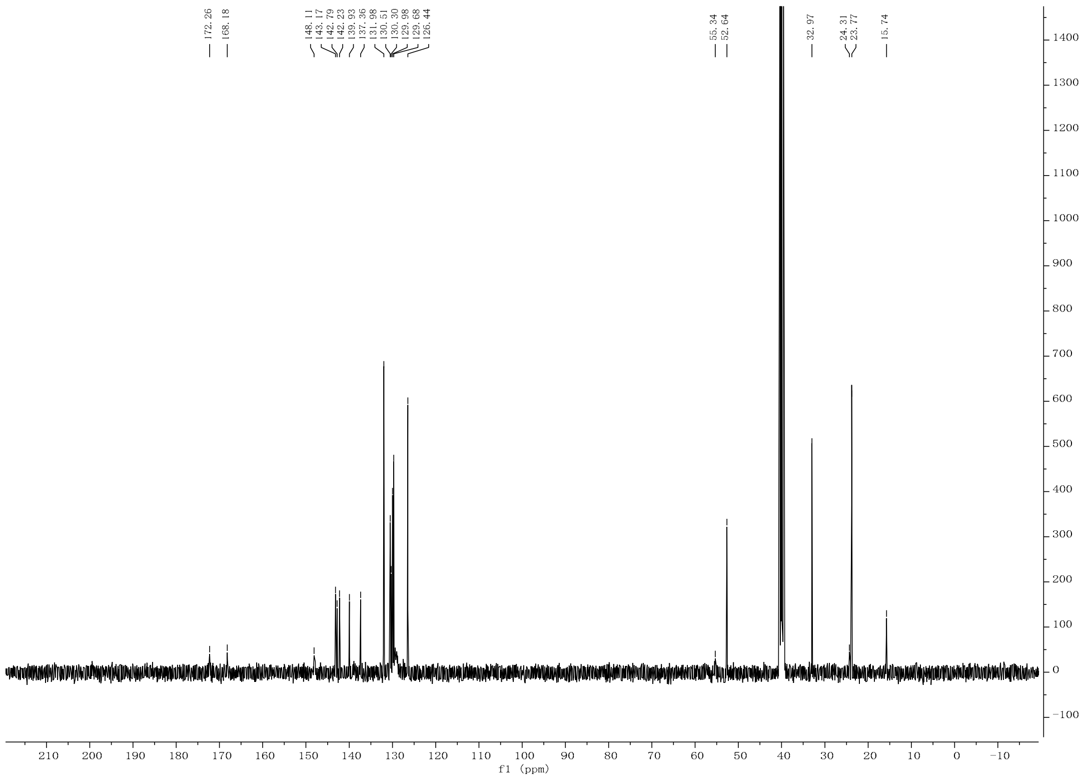

The ^{13}C NMR spectrogram of compound **E14**

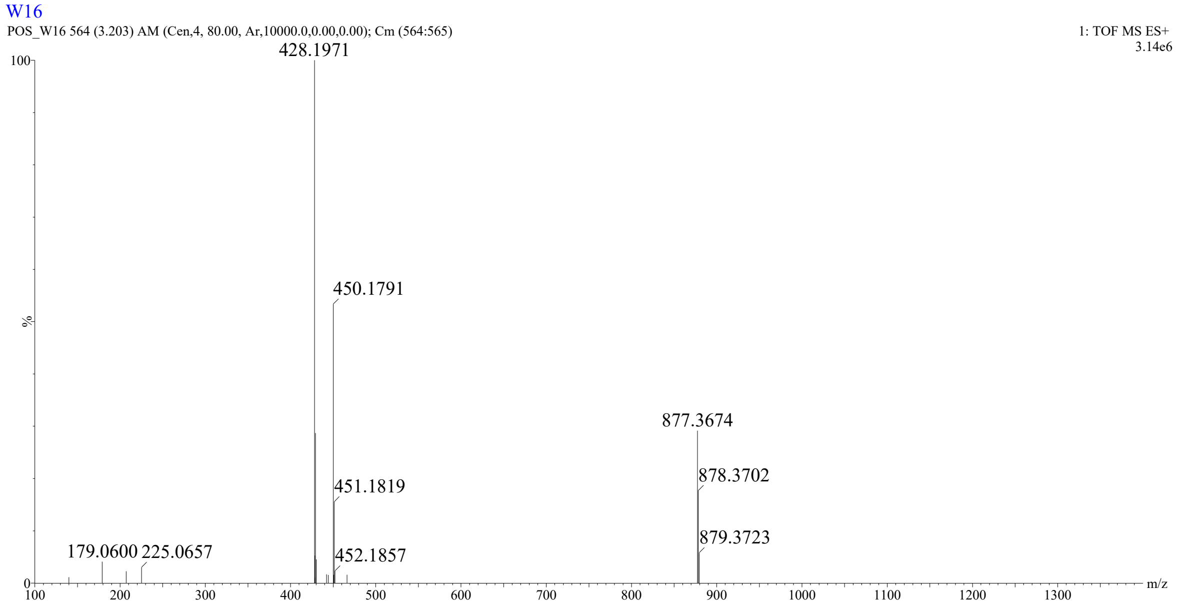

The HRMS spectrogram of compound **E14**

Compound **E15**,

methyl *N*-(2,6-dimethylphenyl)-*N*-(phenazine-1-carbonyl)alaninate

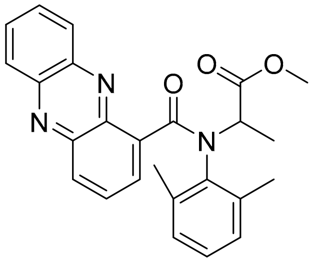

Yellow solid, yield 78.4%, m.p. 150.0-152.0 °C; ^{1}H NMR (400 MHz, DMSO-*d*6) δ 8.28 – 8.18 (m, 2H), 8.13 – 8.07 (m, 1H), 8.05 – 7.95 (m, 2H), 7.80 (d, J = 5.1 Hz, 2H), 6.75 (s, 3H), 4.75 (1H, two isomers), 3.82 (s, 3H), 2.49 (d, J = 9.5 Hz, 6H), 1.14 (3H, two isomers). ^{13}C NMR (101 MHz, DMSO-*d*6) δ 172.72, 168.97, 143.11, 142.35, 142.26, 140.13, 138.27, 136.67 (2C), 132.11, 132.00, 130.59, 130.24, 129.80 (2C), 129.51 (2C), 128.78, 128.50, 128.40, 56.30, 52.39, 19.44, 19.29, 15.49. HRMS (ESI): calcd for $C_{25}H_{23}N_3O_3$ {[M+H]$^+$}, 414.1812; found, 414.1812.

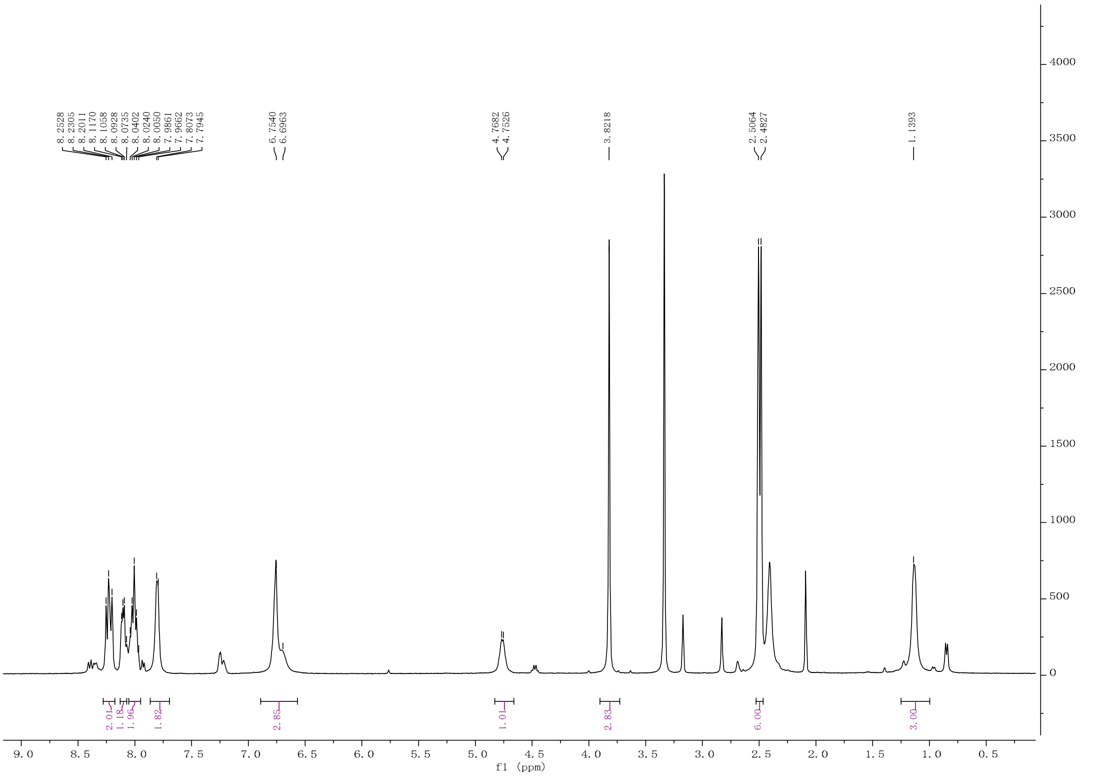

The ^{1}H NMR spectrogram of compound **E15**

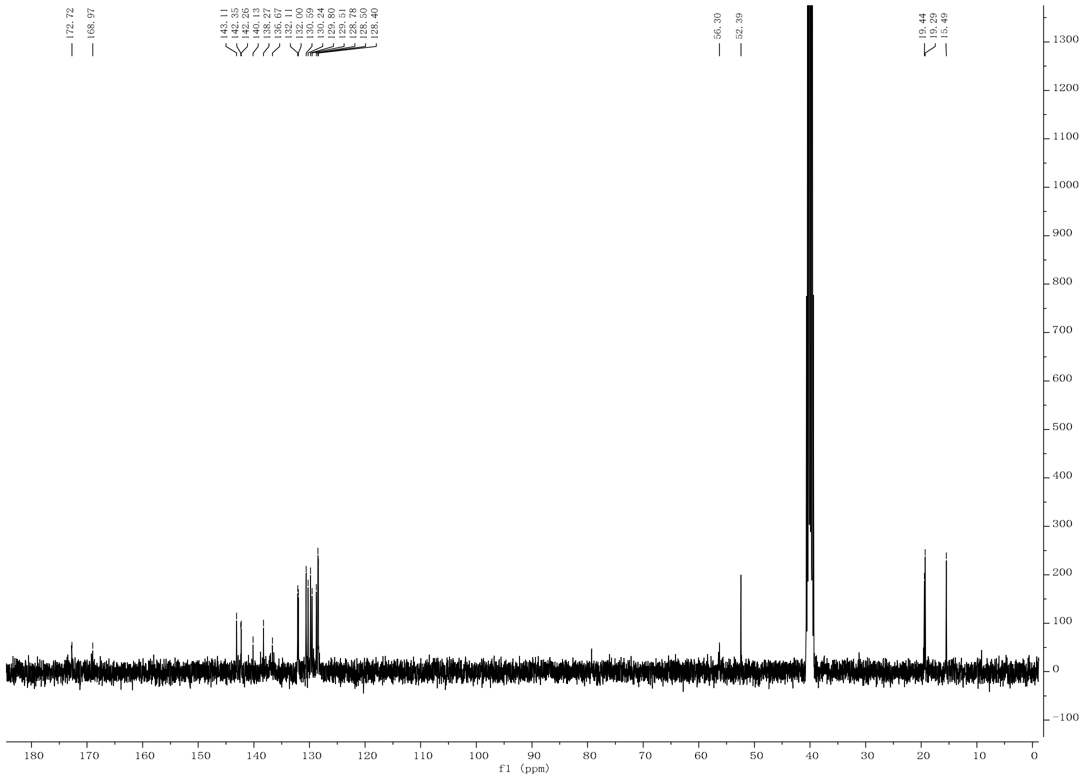

The ^{13}C NMR spectrogram of compound **E15**

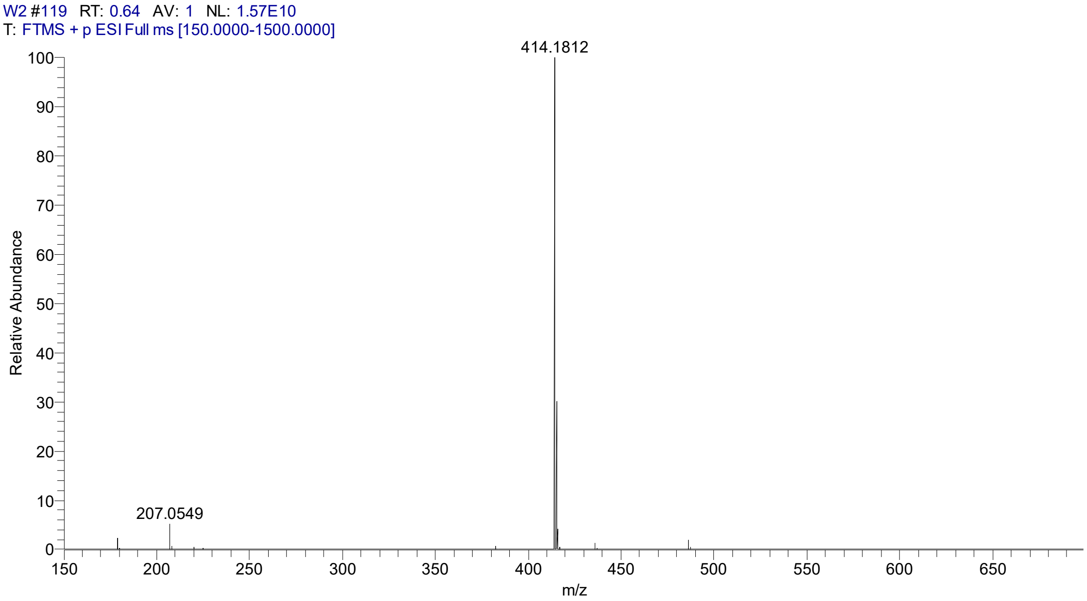

The HRMS spectrogram of compound **E15**

Compound **E16**,
methyl *N*-(3-nitrophenyl)-*N*-(phenazine-1-carbonyl)alaninate

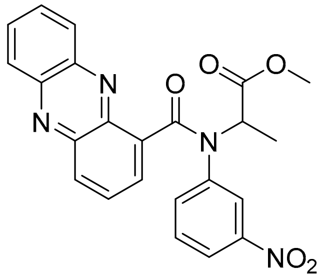

Yellow solid, yield 76.9%, m.p. 166.7-168.4 °C; ^{1}H NMR (400 MHz, DMSO-*d*6) δ 8.33 (s, 1H), 8.16 (dd, J = 18.2, 8.5 Hz, 3H), 8.01 (dt, J = 19.7, 6.2 Hz, 3H), 7.85 (s, 1H), 7.78 – 7.61 (m, 2H), 7.21 (t, J = 7.9 Hz, 1H), 5.30 (1H, two isomers), 3.87 (s, 3H), 1.55 (3H, two isomers). ^{13}C NMR (101 MHz, DMSO-*d*6) δ 172.00, 168.07, 147.24, 143.23, 142.76, 142.10, 139.28, 136.43, 132.18, 131.11, 130.59, 130.03 (2C), 129.86, 129.67, 124.73, 122.79, 112.90 (2C), 108.98, 55.95, 52.85, 15.79. HRMS (ESI): calcd for $C_{23}H_{18}N_4O_5$ {$[M+H]^+$}, 431.135; found, 431.1354.

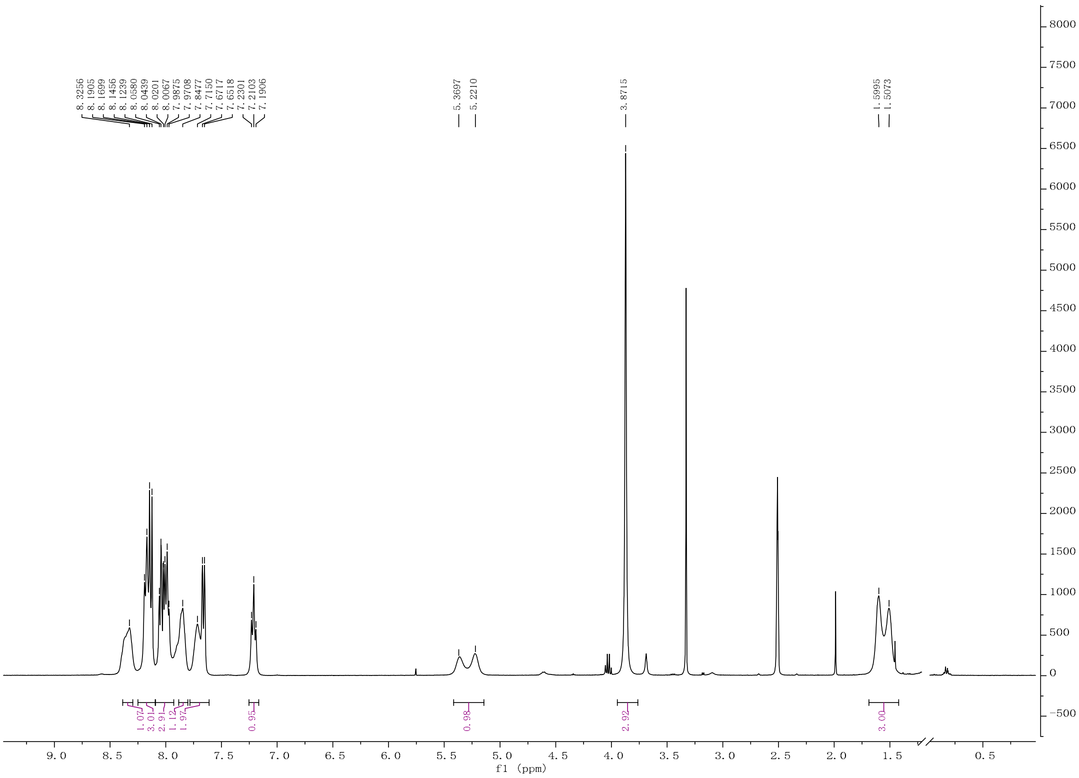

The ^{1}H NMR spectrogram of compound **E16**

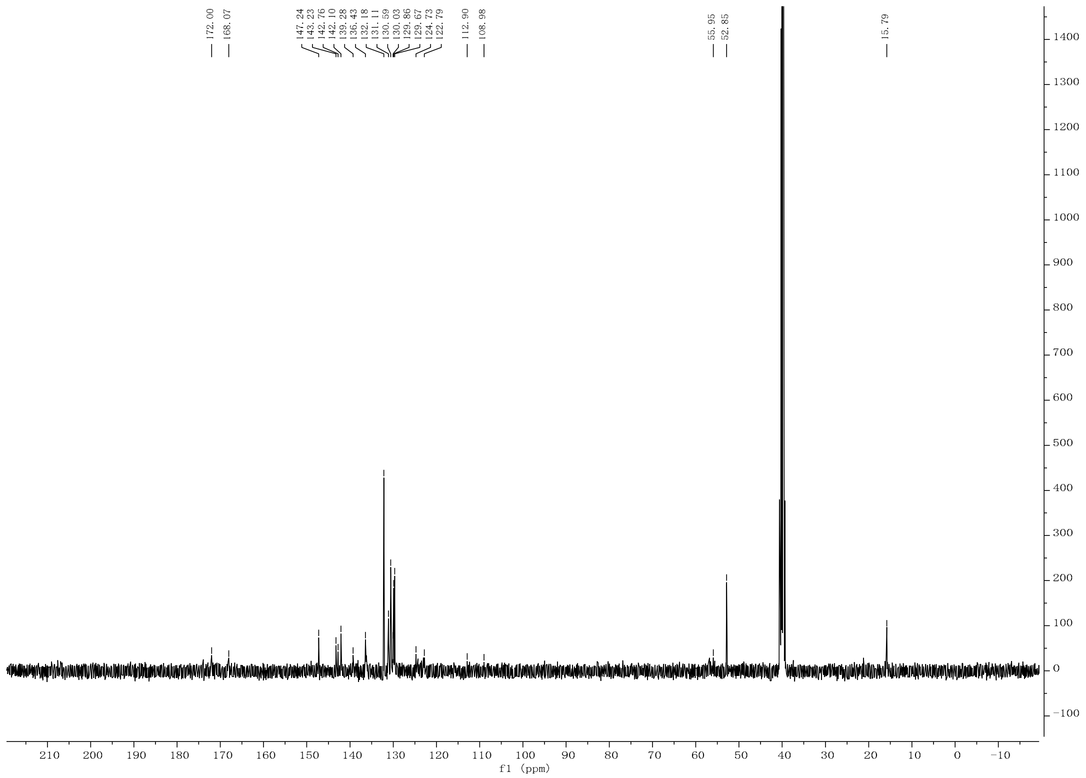

The ^{13}C NMR spectrogram of compound **E16**

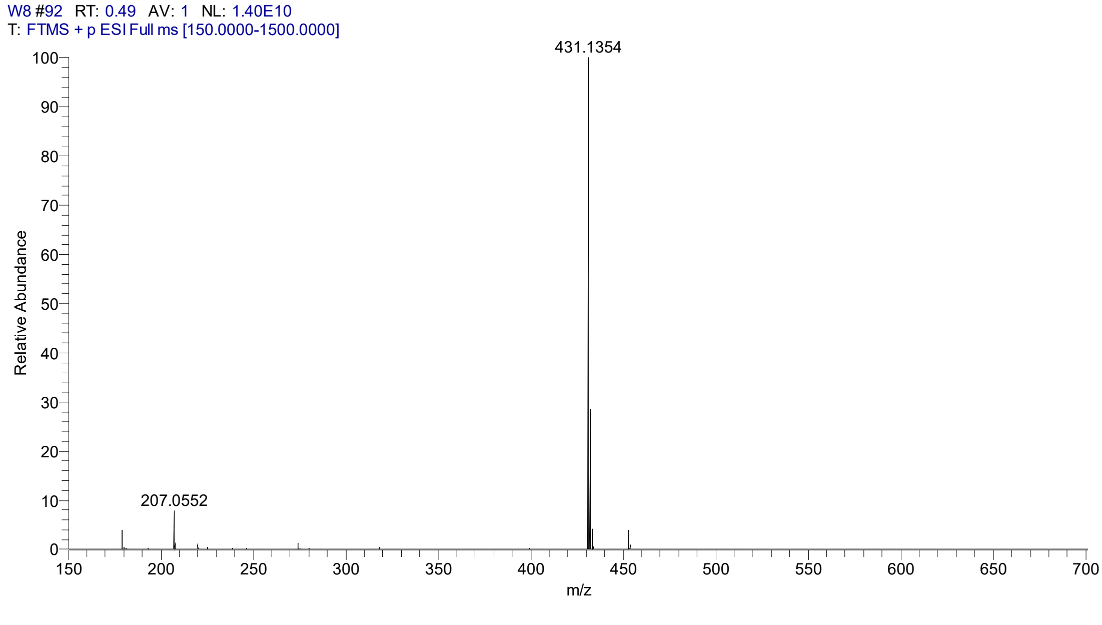

The HRMS spectrogram of compound **E16**
